# Nramp: Deprive and conquer?

**DOI:** 10.3389/fcell.2022.988866

**Published:** 2022-10-13

**Authors:** M. F. M. Cellier

**Affiliations:** INRS-Centre Armand-Frappier Santé Biotechnologie, Laval, Quebec, Canada

**Keywords:** divalent metal-ions, protonmotive force, molecular phylogeny, evolutionary rate-shift, nutritional immunity, horizontal gene transfer, Nramp (natural resistance-associated macrophage protein), MntH (proton-dependent Mn transporter)

## Abstract

Solute carriers 11 (Slc11) evolved from bacterial permease (MntH) to eukaryotic antibacterial defense (Nramp) while continuously mediating proton (H^+^)-dependent manganese (Mn^2+^) import. Also, *Nramp* horizontal gene transfer (HGT) toward bacteria led to *mntH* polyphyly. Prior demonstration that evolutionary rate-shifts distinguishing Slc11 from outgroup carriers dictate catalytic specificity suggested that resolving Slc11 family tree may provide a function-aware phylogenetic framework. Hence, MntH C (MC) subgroups resulted from HGTs of prototype *Nramp* (*pNs*) parologs while archetype Nramp (aNs) correlated with phagocytosis. PHI-Blast based taxonomic profiling confirmed MntH B phylogroup is confined to anaerobic bacteria vs. MntH A (MA)’s broad distribution; suggested niche-related spread of MC subgroups; established that MA-variant MH, which carries ‘eukaryotic signature’ marks, predominates in archaea. Slc11 phylogeny shows MH is sister to Nramp. Site-specific analysis of Slc11 charge network known to interact with the protonmotive force demonstrates sequential rate-shifts that recapitulate Slc11 evolution. 3D mapping of similarly coevolved sites across Slc11 hydrophobic core revealed successive targeting of discrete areas. The data imply that *pN* HGT could advantage recipient bacteria for H^+^-dependent Mn^2+^ acquisition and Alphafold 3D models suggest conformational divergence among MC subgroups. It is proposed that Slc11 originated as a bacterial stress resistance function allowing Mn^2+^-dependent persistence in conditions adverse for growth, and that archaeal MH could contribute to eukaryogenesis as a Mn^2+^ sequestering defense perhaps favoring intracellular growth-competent bacteria.

## Introduction

Detailed phylogenetic analysis allowed deciphering key structure-function relationships in the Slc11 family that comprises the vertebrate Natural resistance-associated macrophage protein (Nramp1) and Divalent metal transporter (DMT1). Slc11 3D architecture fits the “LeuT-fold,” a structural paradigm for the Amino acid-Polyamine-organoCation (APC) superfamily of chemiosmotic transporters or carriers (Pfam Clan CL0062). This Pfam Clan includes the family PF01566, which comprises both the Slc11 family and its phylogenetic outgroup that shows less than 30% sequence identity as well as distinct catalytic specificities (e.g., Nramp-related Mg^2+^ transporters, NRMT) (Cellier, 2012b; Ehrnstorfer et al., 2014; Ponzoni et al., 2018; Ramanadane et al., 2022; Shin et al., 2014; Vastermark et al., 2014; Zallot et al., 2019). Slc11-specific residues in transmembrane segments (TMS) 1&6 are key to H^+^-dependent import of divalent transition metals (e.g., Mn(II), Fe(II), Co(II)), protons co-transport providing the motive force necessary to import Mn(II) against its electro-chemical gradient. In contrast, the corresponding NRMT-specific residues facilitate instead H^+^-independent uptake of Mg(II), thereby indicating type II coevolutionary rate-shifted sites that defined Slc11 specificity (Courville et al., 2008; Ehrnstorfer et al., 2014; Ramanadane et al., 2022).

The APC conserved architecture includes a 10 transmembrane segments (TMS) hydrophobic core formed by juxtaposed copies of a constitutive protomer, the inverted topology repeat LeuT-like, which display little sequence similarity and fold intertwined. It is thought that metal-ion (Me^2+^) displacement of the H^+^ initially bound to outward open Slc11 carrier triggers rocking of a helical bundle (TMS1/2/6/7) over a relatively inert “hash” module (TMS3/4/8/9) that anchors more mobile elements acting as coordinated gates (TMS10, external, TMS5, internal). While Me^2+^ is transported between TMS1, 3, 6 and 8, (symported) protons could travel along a network of ionizable charges distributed across TMS3, 4, 6 and 9 (Bozzi et al., 2019b; Bozzi et al., 2019a; Pujol-Gimenez et al., 2017; Bozzi et al., 2020).

Bacterial Slc11 homologs (H^+^-dependent Mn^2+^ transporters, MntH) are polyphyletic (Cellier et al., 2001). They comprise groups of bacterial origin such as MntH B (MB, present before aerobiosis) and MntH A (MA, characterized in aerobic bacteria such as Firmicutes, Actinobacteria, Deinococcus and Enterobacteria), as well as a group of seemingly eukaryotic descent designated MntH C (MC, eukaryotic cell-derived, subdivided in MCa, MCb, MCg and MCaU) (Cellier, 2012a). Of 2 types of eukaryotic Slc11 only one was apparently transmitted to bacteria: prototype Nramp (pN), as opposed to archetype Nramp (aN, e.g., Nramp and DMT). 3D mapping of Nramp type-specific sites (pN vs. aN) showed potential networks of interacting residues that may influence discrete steps in carrier cycling, which sustained aN divergence in response perhaps to pathogen-driven selective pressure (Cellier, 2016).

3D structures representative of MA and MCb were solved; their detailed comparison revealed many differences such as root mean square deviation of Cα backbone in the areas of helices 9&10 and 4&5, rearranged charge network used for H^+^ translocation, altered geometry of the Me^2+^ binding site (Bozzi and Gaudet, 2021; Antelo et al., 2020). This suggested that evolutionary site-specific variations (Kapli et al., 2020) contributed to fine tune metal uptake through allosteric coupling and/or proton leak regulation for example (Henderson et al., 2019; LeVine et al., 2016; Nelson et al., 2002; Zhang et al., 2020). *MA* gene replacement by *MCb* in select bacterial taxa supports this view. Exploring the evolutionary steps that link MA to MCb, and more generally which enabled Slc11 carrier evolution from bacterial permease (MB, MA) toward eukaryotic nutritional immunity function (Nramp) thus has the potential to reveal the molecular logic of H^+^-dependent Me^2+^ import catalyzed by Slc11 carriers and to indicate functional innovations serving host cell defense (Antelo et al., 2020).

MA-variant group initially uncovered in hyperthermophilic TACK archaea (MntH H) exhibits pseudo-symmetric “eukaryotic signature” marks in TMS4&9 (Cellier, 2012a). By analogy with “eukaryotic signature proteins” which are predominantly found in Asgard archaea (Liu et al., 2021), this suggests that site-specific evolution of MH in archaea relates directly to Nramp emergence in eukaryotes (Antelo et al., 2020; Cellier, 2012a). Resolving Slc11 phylogeny through understanding its natural history, from earth transition from anaerobiosis to aerobiosis, to eukaryogenesis and horizontal gene transfers (HGT) of eukaryotic *pNs* towards bacteria could therefore provide contextualization to interpret structural and functional variations in a family of metal-ion transporters important for nutrition and host-microbe interactions.

## Results and discussion

### Independent HGTs of eukaryotic pNs produced a novel and diverse MntH phylogroup in bacteria

Eukaryotic HGT towards bacteria are rare but several examples have previously been reported (e.g., Supplementary Table S1). As molecular databases expanded, so did diversity of MntH C subgroups and notably that of an initially minor subset closely related to eukaryotic pN (Cellier, 2012a), herein named MCaU as it is related to albeit distinct from MCa (12 TMS vs. 11TMS, and with frequent C-terminally fused universal stress protein (USP)-like peptide). Updated phylogenetic analysis suggested that MCa and MCaU may derive from separate sources of *pN* donor.

**FIGURE 1:**
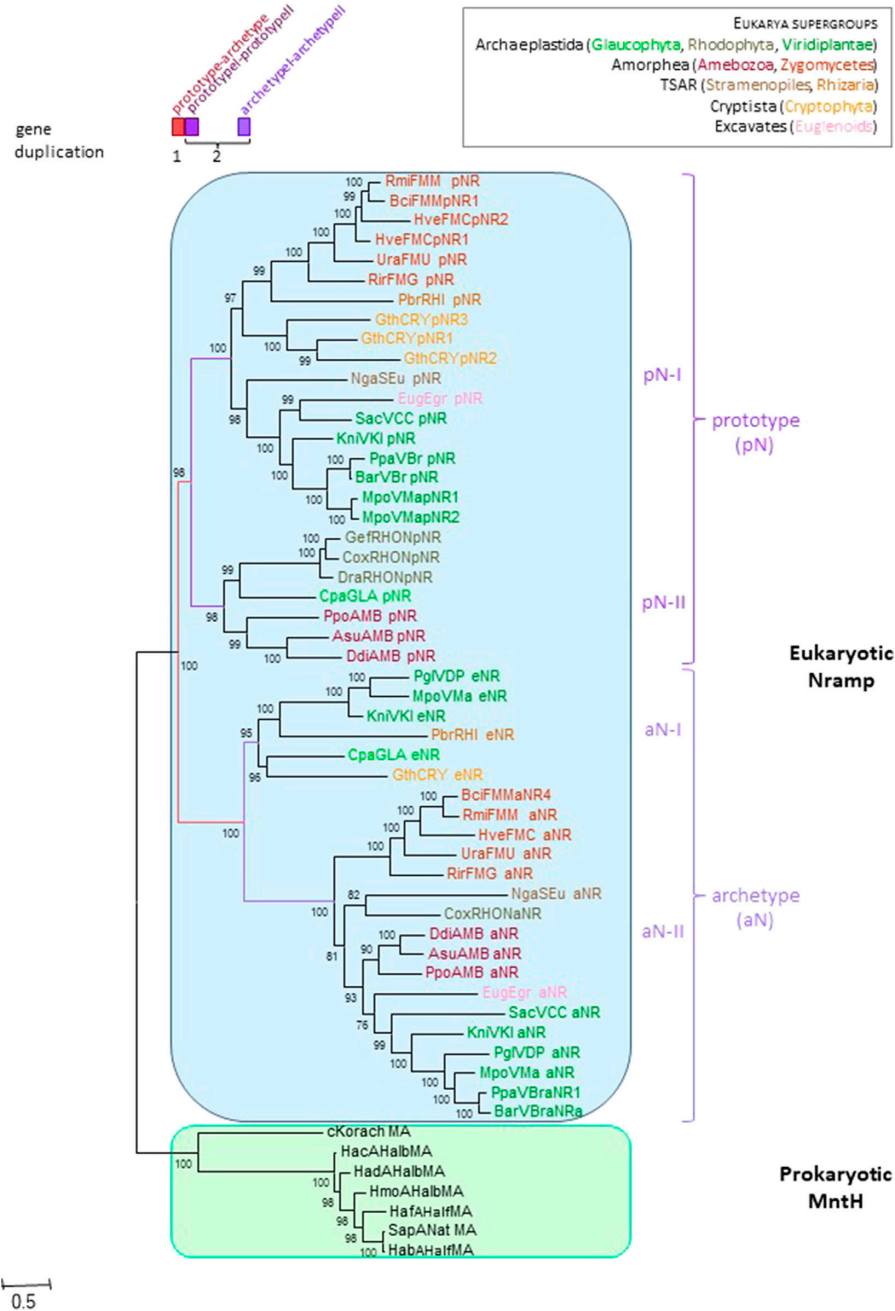
*Nramp* diversification early in eukaryote evolution. The IQ-tree presented used 278 parsimony informative (PI) sites, the substitution model EX3, ML estimate of a.a. state frequency, free rate model of variation among sites with 13 categories (Le et al., 2008; Trifinopoulos et al., 2016). Sequences distribution (*eukaryotic supergroups*): *Amorphea*, Fungi (FMM, MUC, FMC, FMG: pN-I & aN-II) and Amoeba (AMB: pN-II & aN-II); *TSAR*, Stramenopiles (SEu: pN-I & aN-II) and Rhizaria (RHI: pN-I & aN-I); *Archaeplastida*, Green algae (VCC: pN-I & aN-II), Land plants (VKl, VMa: pN-I, aN-I & aN-II; VBr: pN-I & aN-II; VDP: aN-I & aN-II), Red algae (RHO: pN-II & aN-II) and Glaucophyta (GLA: pN-II & aN-I); Cryptophyta (CRY: pN-I & aN-I); Euglenozoa (Eug: pN-I & aN-II). Details are provided in the Supplementary Appendix (p. 11–14).

To examine this possibility, *Nramp* genes from widely diverse eukaryotic supergroups (Burki et al., 2019; Keeling and Burki, 2019) were collected to establish the likely *Nramp* gene complement of the last eukaryotic common ancestor (LECA, Figure 1). A robust phylogeny indicated that successive rounds of gene duplication occurred before LECA, producing *pN* and *aN* parologs initially, and then *pN-I*&*pN-II* as well as *aN-I*&*aN-II* replicates. This result is in line with rapid genome evolution and size increase inherent to eukaryogenesis (Lopez-Garcia and Moreira, 2020; Vosseberg et al., 2021). Notably, pN-Is are made of 11TMS vs. 12 TMS for pN-IIs and aNs. Different combinations of pN and aN isoforms were noted across various phyla, implying gene loss was frequent; for instance, animals conserved only aN-II type. These results identify two possible sources for inter-kingdom HGT (*pN-I* and *pN-II*).

Increased branch length leading to aNs and aN-IIs shows asymmetric evolution (Panchin et al., 2010; Pegueroles et al., 2013) relative to pNs (Figure 1), suggesting sustained aN divergence. Prior electrophysiological studies of pN from ameoba and yeast showed different properties from those of aN (Buracco et al., 2015; Nelson et al., 2002), implying functional diversification. To visualize this in 3D, sequence conservation between pNs and aNs was mapped onto models of the amoebal aN-II (Buracco et al., 2015) threaded in outward open and inward facing conformations based on MCb structures 5M87 (EcoDMT, (Ehrnstorfer et al., 2017)) and 5M94 (ScaDMT, (Ehrnstorfer et al., 2017)), wherein simulated solvent-accessible inner pores (Newport et al., 2019) were displayed (Supplementary Figure S1). MntH Cb structures were used as templates because they belong to the same phylogenetic supercluster (Nramp & MC). Curiously, MCb outward cavity may extend through the membrane along TMS4&9 toward the cell interior while the inward cavity is strictly limited by the Me^2+^ binding site. In contrast, MA corresponding structures (*Deinococcus radiodurans* (Dra) Nramp 6D91 and 6D9W) (Bozzi et al., 2019b) display shallow cavities in both orientations (Supplementary Appendix S1). Importantly, this feature of MCb outward open structure may relate to site-specific DMT1 voltage-dependence and H^+^ uniport activity (Nevo and Nelson, 2004) (cf Supplementary Figure S2, logo #3 site 5), (Pujol-Gimenez et al., 2017) (cf Supplementary Figure S2, logo #6, sites 2,3 and Figure 3B bottom panel).

The 3D pattern of pN-aN sequence conservation highlights Slc11 inner pore in both orientations consistent with critical importance. To investigate aN functional divergence, site-specific evolutionary rate shifts were mapped onto *D. discoideum* Nramp1 3D models (Supplementary Figure S1). All predicted type II (8/8), the majority of type I (4/6) and half of type I/II rate shifts locate to the inner core of the carrier structure and demonstrate possible interactions with residues from neighbor TMS, in either one or both conformations studied. Several candidate divergent sites map to the thick gate area of the inward facing carrier, while another group clusters around the internal gate. Thus, aN divergence, which correlates with phagotrophic way of life in simple eukaryotes (Supplementary Appendix S2) (Buracco et al., 2015; Gawryluk et al., 2019), may reflect site-specific evolution of Slc11 inner core and transport activity (Slodkowicz and Goldman, 2020).

To relate eukaryotic pN isotypes and bacterial MC subgroups non-overlapping sets of sequences (seqs) representing MCa, MCb, MCg and MCaU subgroups were assembled, filtered at given pairwise identity levels, and combined with pN seqs for phylogenetic analyses using MA as prokaryotic outgroup. Sequence patterns iteratively deduced for each MC subgroup (Supplementary Figure S2) generated PHI-Blast hits with distinct taxonomic profiles that indicated subgroup specificity (Supplementary Table S2). Yet, because these patterns were assembled using logos that included all substitutions observed at any site selected for analysis, PHI-Blast outputs were not exempt of cross-contamination, especially for MCa that is the most diverse and prevalent MC subgroup.

Curation of PHI-Blast results using MMseqs2 classification and IQ-Tree phylogenetics (plus the number of TMS (11 vs. 12) to segregate MCa from MCaU, respectively) yielded group-specific, non-overlapping sets of non-redundant seqs (<95% identical, full-length seqs) that establish MCa as the most prevalent subgroup (2368 seqs), followed by MCaU (1161 seqs), MCb (821 seqs) and MCg (398 seqs). Variations in subgroup prevalence may owe to database sequence sampling bias; it could as well indicate distinct ecological niches, more or less conducive for secondary bacterial HGT, or reflect on specific origins.

Reduced sets representative of subgroup diversity (filtered at 60% identity (60% id): MCa, 34 seqs and MCaU, 56 seqs or filtered at 70% id: MCb, 37 seqs, and MCg, 33 seqs) were selected to establish their relationships with pN-I and pN-II while limiting the effect of microbe sampling bias. Phylogenetic trees show MCa seqs are closer to green plant pN-I than MCb seqs (Supplementary Figures S3, S4), and MCg is even more distantly related (Supplementary Figure S5), suggesting that MCa, MCb and MCg could all derive from pN-I isoform(s). In contrast, MCaU appears sister to pN IIs (Supplementary Figures S6A,B). These results indicate a minimum of two inter-kingdom HGT events involving both *pN-I* and *pN-II* types, and leading to evolutionary novel phylogroups of bacterial MntH with MCa, MCb and MCg on one hand and MCaU on the other hand.

### Similarities in the evolutionary pattern of MC subgroups

MC subgroups display commonalities in their phyletic structure that suggest plausible recipient taxa of *pN* transgenes. MCa is markedly present in Alphaproteobacteria (APB, e.g., Rhizobiales (Hyphomicrobiales), Rhodospirillales, Rhodobacterales, Supplementary Table S3) as well as Cyanobacteria, including lineages associated with protistan plankton (endo) symbiosis (Foster and Zehr, 2019). MCa frequency in these APB classes is twice that in Cyanobacteria phylum yet MCa diversity (<70% id) in Cyanobacteria is twice that of APB’s MCa, making *pN-I* HGT toward Cyanobacteria perhaps more likely. Supplementing MCa subset with cyanobacterial seqs (initially represented in 5/34 clusters (60% id) and now filtered at 80% id) showed them as “crown species” of sister clusters that segregate all cyanobacterial MCa on the one hand from a divergent (second) Synechococcal clade on the other hand (Supplementary Figure S3). Seqs diverging basally from each cluster appear diverse and belong to various bacterial phyla (e.g., Proteobacteria and Terrabacteria). Accordingly, HGT of *pN-I* in Cyanobacteria, from a protist source for instance, could lead to MCa lateral spreading across diverse bacteria, including APB (e.g., *Bradyrhizobium* (Hohle and O'Brian, 2012) and *Brucella* (Pitzer et al., 2018) spp. that both rely on MCa to sustain metabolism and virulence). Examples of complete genomes carrying an extra copy of *MCa* were found in diverse bacterial classes typically harboring either *MCg* or *MCaU*, such as Burkholderiales, Sphingomonadales, Xanthomonades and Flavobacteriales, respectively, implying pervasive HGT of *MCa*.

MCb is highly prevalent in Mn-centric Lactobacillales (Bosma et al., 2021), and to lesser extent in Bacillales, suggesting this subgroup originated in one of these taxa (Supplementary Figure S4). Direct *pN-I* HGT seems less likely as MCb are more distantly related to pN-I while *MCa* HGT appears plausible. Some *Lactobacilli* spp. possess up to 3 *MCb* genes per genome (Groot et al., 2005). This suggests positive selection imposed by a competitive ecological niche, such as the gut of insects feeding on flowering plants and/or food processing environment (Duar et al., 2017; Khan et al., 2020). This selective pressure could favor HGT of *MCb* among bacteria sharing the same niche. Among these, the enterobacterium *Sodalis praecaptivus* possesses an apparently functional *MCb* in addition to *MA* while several related spp. (e.g., some *Sodalis*-allied symbionts; obligate endosymbionts such as *Baumannia* and *Wigglesworthia*) maintained solely *MCb* copy, similarly to few Orbales and Moraxellales spp. also found in the gut of insects (*Gilliamella* and *Acinetobacter*, respectively) (Supplementary Appendix S3). Besides, ∼20% of Bifidobacteria (Actinomycetia), mostly species associated with pollinating insects (Khan et al., 2020), carry a *MCb* gene instead of *MA*. Notably, *Lactobacillus spp.* can outcompete fungal growth by scavenging available Mn (Bosma et al., 2021), and along similar lines *Staphylococcus aureus MCb* (Horsburgh et al., 2002), *Streptococcus agalactiae MCb* (Shabayek et al., 2016) and *Enterococcus faecalis MCb1*&*2* genes (Colomer-Winter et al., 2018) all contribute to Mn^2+^ acquisition and bacterial virulence. MCb subgroup structure may thus reflect adaptation to a narrow food/gut related niche, where competition for metal acquisition may sustain MCb diversity.

MCg is mostly found in Proteobacteria (GPB, Pseudomonadales and BPB, Burkholderiales). Its distance from pN-I cluster suggests that MCg, like MCb, could derive from secondary *MCa* HGT. A more ancient *pN-I* HGT seems less likely given MCg relatively reduced diversity and taxonomic distribution. While MCg abundance in Pseudomonadales owes to sequencing bias (*Pseudomonas* spp.) Burkholderiales from several families found in the rhizosphere (Ling et al., 2022) (e.g., Comamonadaceae, 69 spp., Oxalobacteriaceae, 102 spp. and Burkholderiaceae, 511 spp.) carry distinct *MCg* genes (1-2/genome, and in various combinations with *MAV* genes -nested relatives of *MA*- (Cellier et al., 2001), mainly in Burkholderiaceae, BPB, cf Supplementary Table S2). *MCg* genes were also found in APB and GPB classes known to populate the rhizosphere (e.g., Sphingomonadales and Xanthomonadales) (Ling et al., 2022), implying a possible role for MCg in adaptation to this niche. Burkholderiaceae spp. are metabolically versatile and can associate with various hosts, such as fungal endobacteria (Okrasinska et al., 2021), legume root nodule symbionts (Hassen et al., 2020) and opportunistic pathogens (Schaefers, 2020). One of *B. pseudomallei MCg* genes (from the most divergent cluster, Supplementary Figure S5) is induced during macrophage infection and likely influences bacterial virulence (Shalom et al., 2007), which depends on Mn^2+^ acquisition (DeShazer, 2019). The data suggest that *MCg* genes may contribute to bacterial adaptation to various hosts and point at plant rhizosphere as an ecological niche conducive to *MCg* HGT.

MCaU subgroup, uniquely derived from pN-II, is found principally in Bacteroidetes (Flavobacteriales, Sphingobacteriales, Chitinophagales and Cytophagales) and it is abundantly represented in environmental metagenome samples affiliated to this order (Supplementary Figure S6A). Known example of Flavobacteria endosymbiont of amoeba (Corsaro et al., 2017) suggests a possible setting for *pN-II* HGT from eukaryote to prokaryote. Phylogenetic analysis shows MCaUs from Bacteroidetes classes forming a “crown cluster” while seqs from other taxa lie between Bacteroidetes and pN-II, in a succession of separate branches (Supplementary Figure S6A) that suggests their position may owe to long branch attraction (LBA) artefact (Kapli et al., 2020). Also, substantial divergence observed for *Elizabethkingia sp.* MCaU, which seems consistent with decaying copies of both *MCa* and *MCaU* on *E. meningoseptica* (KC1913) chromosome (not shown), provides a reminder that horizontally acquired genes require suitable conditions to remain functional. The data presented suggest some Bacteroidetes ancestor as recipient of *pN-II* HGT and *MCaU* propagation among diverse taxa sharing the same environmental niche.

Several examples of *MC* HGT come from human pathogens. Replacement of *MA* by *MCb* was noted in *Bifidobacteria* and other obligate anaerobes (*Actinomyces*, *Cutibacteria*), some facultative anaerobes (Orbales: *Morganella*) and obligate aerobe (*Corynebacterium falsenii*). In contrast, in Listeriaceae (Orsi and Wiedmann, 2016) MCb is found in non-pathogenic *Listeria* spp. while *L. monocytogenes* and its close relatives carry *MCa* genes, implying that *MCa* HGT preceded *L. monocytogenes* pathogenic adaptation. These observations suggest adaptating MC carrier activity is common and may support various host-microbe interactions.

Horizontal dissemination of integral membrane proteins implies evolutionary success (Arnold et al., 2022) and MC activities may confer to recipient bacteria some functional advantage inherited from eukaryotic ancestry. For instance, Nramp may differ from bacterial MA in their use of the protonmotive force to drive metal-ion import because membrane potential values vary between bacteria and eukaryotic cells depending on the site and type of respiratory processes (Benarroch and Asally, 2020; Kashket, 1985; Tran and Unden, 1998). Adapting functional innovation from eukaryotic Nramp through MC diversification may advantage recipient bacteria to acquire metal-ions from selective niches.

### MntH H: Archaeal link between bacterial MA and eukaryotic Nramp

As Nramp closest bacterial homologs (MCs) were in fact derived from pNs, and given the distance separating pNs and MA (e.g., Supplementary Figures S3–S6), an evolutionary intermediate linking MA to Nramp ancestor is theoretically plausible. This “missing link” could inform on Slc11 carrier evolution in the context of eukaryogenesis and host cell resistance to infection. Candidate seqs, which resemble MA but display some “eukaryotic-signature” functional marks, were identified in hyper-thermophilic archaea and designated MntH H (MH) (Cellier, 2012a).

To characterize MH PHI-Blast analyses were conducted to survey Slc11 groups of prokaryotic origin (MB, MA, MAV and MH) and compare their taxonomic profiles (Supplementary Table S2). Both MB and MA display relatively broad distribution, similar to MCa and MCaU while MAV is principally found in MCg bearing spp., e.g., Burkholderiales (Supplementary Table S2). Notably, MB is confined to anaerobic bacteria, including *Dictyoglomus* and *Caldisericum* spp. previously proposed to have originated before onset of earth aerobiosis (Betts et al., 2018) as well as *Chlorobium* and *Geobacter* spp. that together catalyze light-driven anaerobic oxidation of Mn^2+^ (Daye et al., 2019) (Supplementary Table S4). MA on the other hand constitutes one of MntH most prevalent groups in Bacteria and also includes few archaeal homologs (in Halobacteriales and Haloferacales). In stark contrast, MH was found mainly in TACK, DPANN and Asgard archaea, phyla that comprise the currently closest known prokaryotic relatives of the first eukaryotic common ancestor (FECA).

**FIGURE 2:**
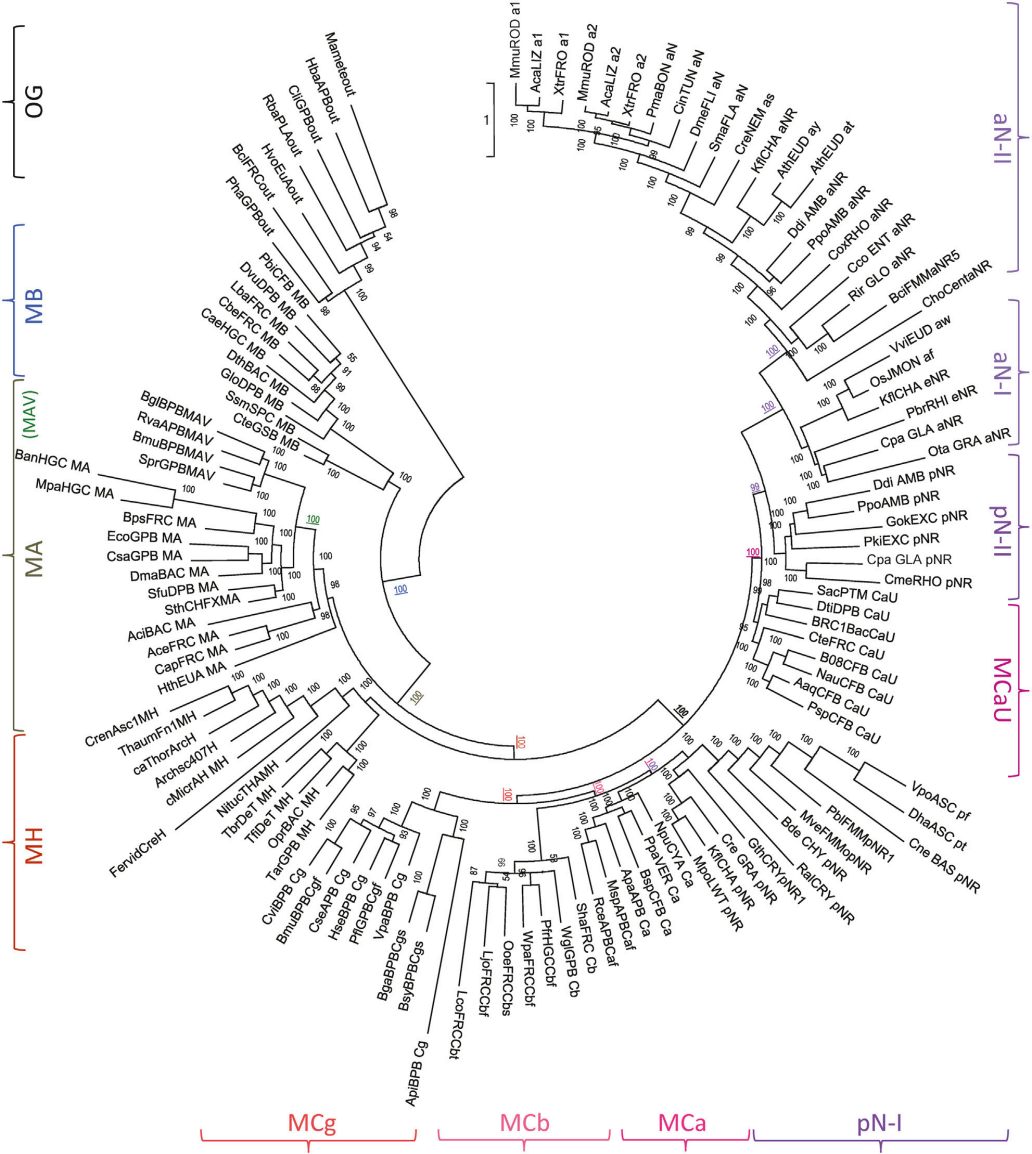
MntH H: prokaryotic precursor of Nramp. Phylogenetic analyses using 379 Slc11 PI sites identify MH as an evolutionary intermediate between MntH homologs of bacterial origin (MB, MA) and eukaryotic Nramp (aN and pN types, including MCs). The IQ-tree presented was inferred using the substitution model UL3, ML estimate of a.a. state frequency, free rate model of variation among sites with 19 categories (Le et al., 2008; Trifinopoulos et al., 2016). Symbols indicating Slc11 phylogroups are placed at the periphery of the tree and the confidence values for key nodes (underlined) are color-coded accordingly (OG, outgroup; MB, MntH B; (MAV, MntH AV), MA, MntH A; MH, MntH H; MCg, MntH Cg, MCb, MntH Cb, MCa, MntH Ca, pN-I, prototype Nramp I; MCaU, MntH CaU; pN-II, prototype Nramp II; aN-I, archetype Nramp I, aN-II, archetype Nramp II). Sequence details in the Supplementary Appendix (p. 15–23).

To establish MH position in Slc11 phylogeny analyses encompassing both prokaryotic and eukaryotic sequence diversity were performed (Figure 2). The results indicate that MB diverged first after emergence of the Slc11 family, supporting the possibility MB represents a remnant of an early form of Slc11 that fulfilled H^+^-dependent Mn^2+^ import before aerobiosis. MA group evolved after and appears more diverse, including MAV subgroup. Strikingly, MH stands sister to the Nramp-MC supercluster, as would be expected for a close relative of Nramp ancestor. The position of MC subgroups relative to each other confirms that MCb and MCg may derive from MCa, which is the closest relative of pN-I. The absence of clustering between pN-II and MCaU can be imputed to MCaU intrinsic diversity (Supplementary Figure S6A). Only aNs form a monophyletic cluster (vs. pNs), as expected from pNs diversity (cf Figure 1). Slc11 family tree therefore places MH on the evolutionary path linking bacterial MA to eukaryotic Nramp.

Yet MH was found in both archaea (AH) and bacteria (BH). Since AH and BH segregated as sister species in Slc11 phylogeny, specific patterns were derived from each subgroup for additional PHI-Blast analyses. AH is present in at least 70 microorganisms, all from TACK (53), DPANN (7), Asgard (1) and unclassified archaea (6), except 2 candidate CPR bacteria: *Peregrinibacterium* and *Kaiserbacterium*. Detection of AH seqs in CPR bacteria suggests recent HGT of *AH* into closely associated bacteria.

At least 42 microorganisms encode bacterial MH (BH), mainly (D-T) Thermales (16), (GPB) Thiotrichales (10), (APB) Rhizobiales (7), (CFB) Ignavibacteria (3), 1 Planctomycetes and 1 Chloroflexi bacteria. Filtering PHI-Blast outputs at 95% identity gave 100 AH seqs and 31 BH seqs, indicating higher prevalence in archaea. Filtering at 70% identity yielded 32 (AH) and 10 (BH) curated seqs, demonstrating diversity in both subgroups. Higher prevalence of AH and evidence of recent HGT toward bacteria both suggest AH predated BH.

MH seqs (filtered at 70% id) were used to detail AH and BH relationship to pNs (using MB as outgroup, Supplementary Figure S7). MH is sister to pN group, implying MH predated MC subgroups. BH conserved haplotype suggests a common origin for BH seqs (Supplementary Figure S7B) although contrarily to AH, they do not appear monophyletic (Supplementary Figure S7A). This may result from LBA artefact caused by the most divergent sequences. In contrast, *Thorarchaeota* AH sampled on 3 continents form a clade suggesting vertical inheritance of *MH* in Asgard (Supplementary Figure S7A, inset). Considering AH dominance over BH together with evidence of recent HGT of *BH* genes among Thermales (e.g., plasmid pAA3-7b, Supplementary Figure S7A) and Thiotrichales, which may both share with hyperthermophilic TACK archaea such ecological niche as hydrothermal vents (Copeland et al., 2012; Dombrowski et al., 2017; Scott et al., 2019; Zaremba-Niedzwiedzka et al., 2017), the data suggest BH basal position in MH clade could indicate *AH* HGT toward bacteria.

Based on currently favored model for eukaryogenesis (Liu et al., 2021), AH position in Slc11 phylogeny may reflect the evolutionary transition from prokaryote to eukaryote. MH 3D model structure displays adjacent “eukaryotic-signature” marks located in pseudo-symmetric TMS (4, Asp and 9, Arg + 3 residues) with both charges previously shown to influence DMT1-dependent metal uptake (Courville et al., 2006) and expanding MA network of ionisable sites implicated in voltage sensing and directional H^+^ transport (Bozzi et al., 2019a; Bozzi et al., 2019b; Bozzi et al., 2020; Ehrnstorfer et al., 2017; Pujol-Gimenez et al., 2017). Accordingly, AH “eukaryotic-signature” marks could improve H^+^-dependent Mn import activity in pre-eukaryotic cells and pave the way to Nramp role in nutritional immunity.

### Stepwise evolution of the use of the protonmotive force in the Slc11 family

Slc11 H^+^-network regulates Me^2+^ uptake in response to variations in the concentration of protons on both sides of the membrane (delta-pH) and/or variations in the electrochemical potential of the membrane (delta-psi), which together generate the protonmotive force. It was established that *S. cerevisiae* pN-I (Smf1p), rat aN-II (DCT1/DMT1) and *D. radiodurans* MA (Dra Nramp) all exhibit variable stoichiometry in H^+^/Me^2+^ symport resulting from uncoupled co-substrate uptake. Uncoupled uptake of Na^+^ (Smf1p) or H^+^ (DMT1) was proposed to regulate toxic accumulation of metal-ions in certain conditions, such as respectively, alkaline pH or acidic pH and negative membrane potential (Bozzi and Gaudet, 2021; Nelson et al., 2002).

**FIGURE 3:**
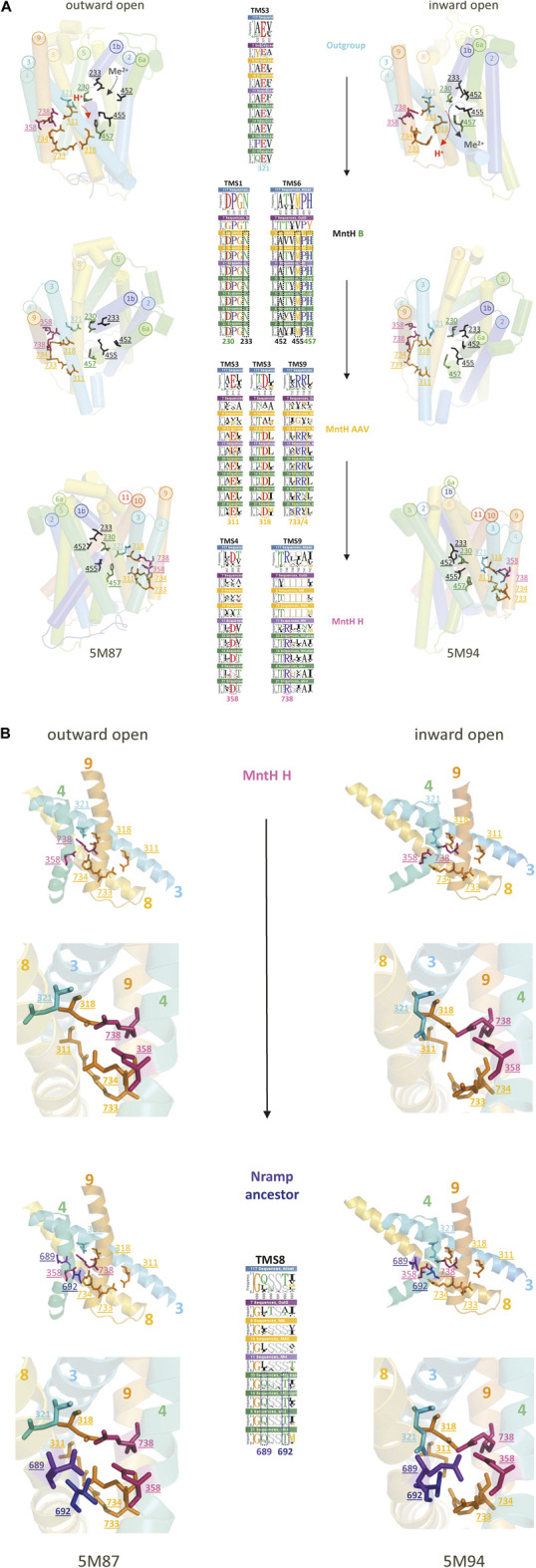
(Continued). Stepwise evolution of Slc11 proton translocation pathway. **(A,B)** MCb 3D structures from *E. coleocola* (outward open, 5M87) and *S. capitis* (inward open, 5M94) were used to display with a color code stepwise fixation of the residues forming Nramp proton translocation pathway, demonstrated by logos (insets) of Slc11 phylogroups and outgroup (Figure 2, 117 seqs in total): outgroup (7 seqs); MB (9 seqs); MAAV (16 seqs); MH (11 seqs); pN-I, MCg, MCb, MCa (33 seqs); pN-II, MCaU (14 seqs); aN-I (6 seqs); aN-II (21 seqs). The position in Slc11 family msa of each evolutionary rate-shift studied is highlighted with colors that indicate whether Nramp residue was already present in the outgroup (cyan) or predated MB (black, substrate binding site, green, H^+^ pathway), MA (orange), MH (pink) or Nramp (violet, blue) emergence. The corresponding MCb residues are shown in the 3D cartoons as sticks colored accordingly and identified by their position in Slc11 family msa. **(A)** *In prokaryotes*. Three views picturing alternate conformations display residue motion during cations transport cycle. **(B)** *In eukaryotes*. Detail of the H^+^ translocation pathway shows two mutations introducing novel polar side chains in Nramp TMS8. Their potential interaction with MH inherited charge network, at the interface of helices forming the “hash” module are presented in general and close-up views.

To examine how Slc11 H^+^-network evolved, sequence logos representing Slc11 phylogroups and outgroup (Figure 2) were compared at target sites (TMS1, 3, 4, 6 and 9; Figure 3). The outgroup exhibits only 1 conserved charge part of Slc11 H^+^-network, in TMS3 (ancestral Glu), which assumes distinct orientations in NRMT and MntH (Supplementary Figure S8) and rearranges upon Mn^2+^ binding by MntH (Figure 3A). Prior to MB emergence, 5 co-evolved sites in TMS1&6 (Asp, Asn and Ala, Met, His, respectively) enabled both counter-selecting Mg^2+^, most likely due to steric constraint preventing the binding a hydrated Mg^2+^ ion, and allowing instead Mn^2+^ import coupled to H^+^ uptake (Chaloupka et al., 2005; Ehrnstorfer et al., 2014; Makui et al., 2000; Ramanadane et al., 2022). Toward MA, 4 novel charges were selected in TMS3&9 (Glu, Asp and Arg, Arg, respectively) to create a divergent H^+^ translocation pathway linked to TMS3 ancestral charge (Bozzi et al., 2019a). Transition to MH expanded this H^+^-network with adjacent charges in TMS4&9 (Asp & Arg) while altering the inner and outer gate topology (based on MCb, Supplementary Figure S8). Lastly, 2 polar sites were introduced in TMS8 of Nramp ancestor, which line the extended outer cavity (Supplementary Figure S1) and regulate DMT1 voltage-dependence and H^+^ influx (Pujol-Gimenez et al., 2017).

These molecular data demonstrate stepwise evolution of Slc11 H^+^-network that recapitulates the successive emergence of phylogroups, from the family origin to Nramp ancestor, and therefore indicate the phylogeny inferred is correct. First there was selection for coupled uptake of Mn^2+^ and H^+^; with aerobiosis onset and increased respiratory rate an alternative H^+^ uptake pathway was accommodated (uncoupling), then modified in archaeal pre-eukaryotic cells and completed in FECA. This suggests that stepwise evolution of Slc11 phylogroups reflects functional adaptations to environmental changes (Sassa et al., 2021), including the chemical (ΔpH) and electrical (ΔΨ) components of the H^+^ electrochemical gradient of their respective membrane.

To determine which step was critical, coevolution networks (Fonseca et al., 2021) were deduced from Slc11 family sequence alignment. The results associate the transition from outgroup to Slc11 ancestor with the bulk of rate-shifted sites that were detected as a single collection, and which comprises sites that either remained conserved across the family or later coevolved. Small collections of coevolved rate-shifts corresponding to MA, MH or MC and Nramp phylogroups were also noted. Mapping these collections on models of MB 3D structure suggested each evolutionary wave occurred in select 3D locations, implying that evolution of Slc11 carrier activity also relied on allosteric networks.

### Toward understanding of the molecular logic of Slc11 carriers

**FIGURE 4:**
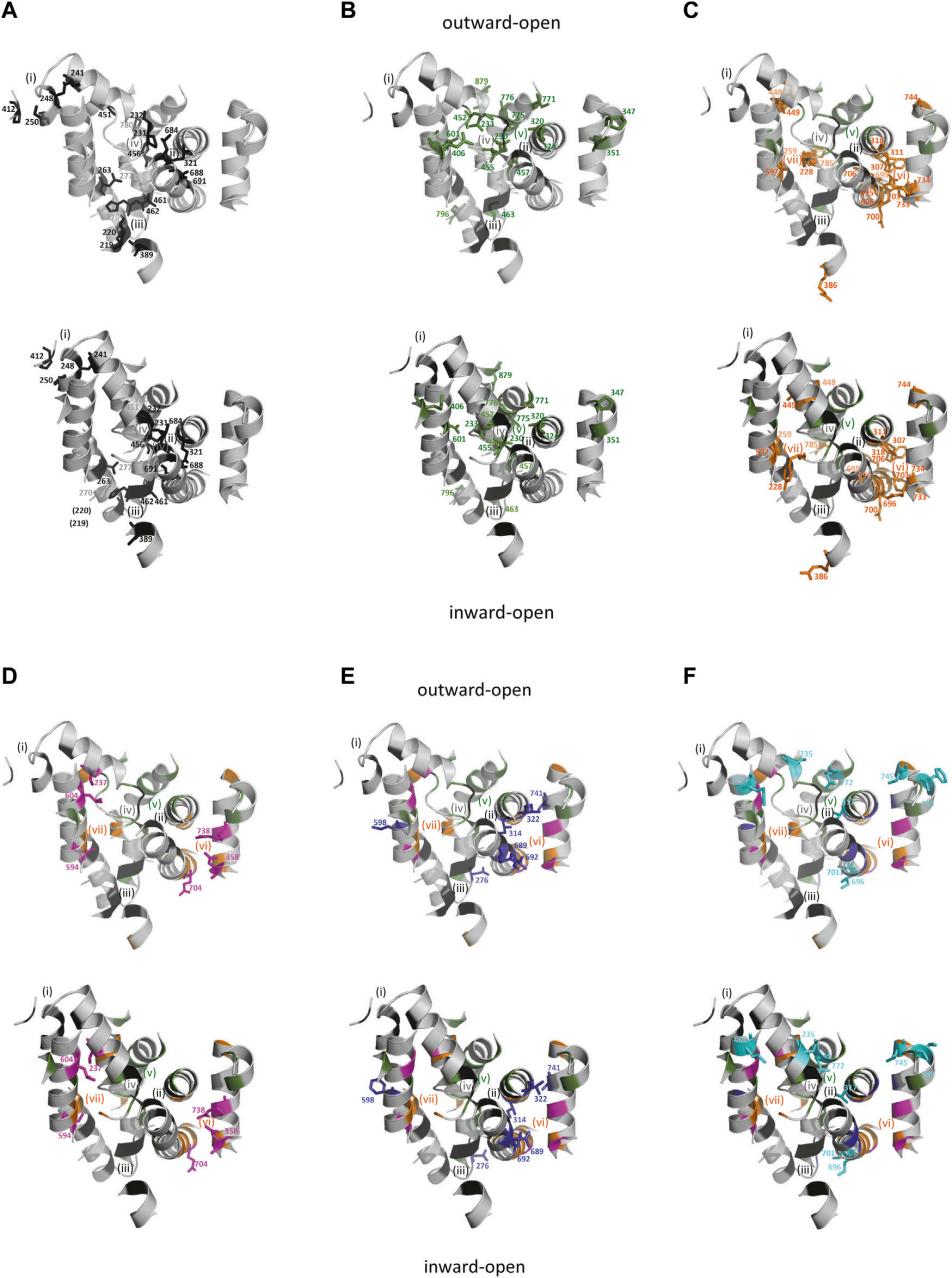
(Continued). Successive waves of co-evolved sites affected distinct areas of Slc11 carriers. Sites are illustrated by the corresponding residues from EcoDMT (top panel) and ScaDMT (bottom panel), shown as colored sticks to distinguish evolutionary classes and numbered according to their position in Slc11 family msa marked by matching color dots (Supplementary Figure S9). **(A)** Sites conserved between the outgroup and Slc11 families are located in areas (i) C-end of TMS1b/TMS2 N-end/loop 5/6, (ii) mid-TMS1 extended segment/TMS3 C-terminal third/TMS8 N-half, (iii) TMS1a N-end/mid TMS6b segment/TMS2 C-end/TMS5 N-end, plus the “loose” cluster (iv) formed by sites located at TMS6a C-end/TMS6b N-end/TMS10 C-terminal third and loop 2/3; **(B–F)**, Evolutionary rate-shifts that predated successive phylogroups occupy distinct locations: MB **(B)** in area (v) including TMS1 and TMS6 central extended segments/TMS7 C-third/TMS3 C-third/TMS10 central portion/mid-TMS11 plus two sites at TMS5 C-end and TMS4 N-end; MA **(C)** in areas (vi) TMS3 N-half/TMS8 C-third/TMS9 N-end and (vii) involving TMS1a C-end/mid-TMS2/TMS7 C-third plus sites in TMS6b, TMS5 N-end and TMS10 C-end; MH **(D)**, sites in areas (i) at mid-TMS1b/TMS7 C-end, (vii) mid-TMS7 and (vi) at mid-TMS4/mid-TMS9/TMS8 carboxy-end; ancestral Nramp **(E)**, sites extending area (vi) TMS3 C-half/TMS8 N-half; aNs (**F**, cyan), including polar sites in TMS1b/mid-TMS3/TMS10 N-third.

Serial evolutionary changes can improve allosteric coupling of cations import (Zhang et al., 2020) while modulations of voltage-dependency and driving cation leak may regulate carrier stoichiometry (Bozzi et al., 2019a; Bozzi et al., 2019b; Nelson et al., 2002). To investigate sequential steps in Slc11 evolution, sites from the multiple sequence alignment (msa) that behaved similarly to those forming Slc11 H^+^-network (conserved rate-shift correlated to phylogroup emergence, Supplementary Figure S9; Supplementary Table S5) were mapped onto MCb structures in outward-open and inward-open conformations (Figure 4).

Ancestral sites shared by outgroup and Slc11 families indicate conservation of structural aspects of the carrier cycle, such as rocking of the TMS1/2/6/7 helical bundle, including 3D local networks of potentially interacting residues in areas i-iv (Figure 4A). All these networks rearrange spatially during bundle rocking upon transition from outward- to inward-open conformations. Thus Outgroup/Slc11 conserved sites may primarily contribute to structural reorganization upon substrate binding and occlusion.

Slc11-specific sites that remained conserved since MB emergence define (v) a novel, large area sitting in the middle of the membrane in the plane that accommodates the co-substrate binding sites. This broad network connects areas (ii) and (iv) through specific binding of Mn^2+^ and H^+^ co-substrates and mediates closure of the external gate (TMS6b-TMS10). Other isolated sites shift spatially upon opening of Slc11 internal gate. Slc11-specific sites thus likely contribute to couple co-substrate binding and carrier gating.

Slc11-conserved sites that fixed before MA divergence populate various locations surrounding the co-substrate binding cavity (v). These include a second large area (vi), situated within the “hash” module below the plane accommodating the co-substrate binding sites, which opens a H^+^ translocation pathway toward the cytoplasm, and another local network (vii) that is rearranged during carrier cycling. Additional sites fixed in MA extend previous networks (i), (iii) and (iv). Accordingly, coevolutionary rate-shifts dating back to MA ancestor allowed reengineering of Slc11 carrier by opening a divergent intracytoplasmic H^+^ release pathway and fine-tuning of local rearrangements that accompany carrier cycling.

Nramp residues inherited from MA transition to MH appear to consolidate pre-existing networks (i) and (vii), with sites located in mid-TMS1b/TMS7 carboxy-end and mid-TMS7, respectively, and (vi) with 3 potential charges added. These evolutionary changes could tweak structural rearrangements within the helical bundle and extend the H^+^ translocation pathway toward the cytoplasm on one hand and toward TMS1 Me^2+^ binding site one the other hand (Figure 3) thus likely altering Slc11 catalytic activity.

FECA’s Slc11 (i.e., ancestral Nramp) had consequently amended network (vi), now extending upward along TMS3&8 and vis a vis TMS4&9 by adding polar residues presumably adjusting paths for H^+^ translocation and membrane potential sensing (Pujol-Gimenez et al., 2017). Rearrangement of TMS8 sites upon carrier cycle (Figure 3B) suggests their coevolution altered Slc11 carrier activity and could favor functional diversification of pNs and aNs during progression from FECA to LECA. While aN Me^2+^ import efficiency was shaped by evolving at host-bacteria interface, opportunistic bacterial recipients of *pN* HGT could benefit from using neo Slc11 carriers functionally distinct from MB, MA and MH.

aNs represent nutritional immune effectors that exert direct antibacterial activity by competing for import of extracellular Me^2+^ and they sustain growth of organisms from different eukaryotic supergroups that graze on bacteria (Buracco et al., 2015; Gawryluk et al., 2019). aN-shifted sites correspond in majority to aliphatic residues that may optimize carrier stability plus 3 polar side chains that line above the outer co-substrate binding cavity (v), of which rat aN-II (DCT1 aka DMT1) E122 in TMS1b was involved in metal ion binding and specificity (Courville et al., 2006). Improving Slc11 catalytic performance thus represents a plausible selective pressure that drove aN divergence.

Strong directional selection can enhance functional evolvability, by maintaining protein foldability and stability (Zheng et al., 2020). As patterns of sites that coevolved during key phylogenetic transitions identify discrete 3D networks of residues that progressively amended Slc11 local structure-function relationships some molecular logic may be viewed in the elaboration of an ever more efficacious H^+^-driven Mn^2+^ pump, which ultimately capitalized on the protonmotive force generated by the eukaryotic H^+^-ATPase.

### Alphafold models support MntH polyphyly and structural diversification of MCs

**FIGURE 5:**
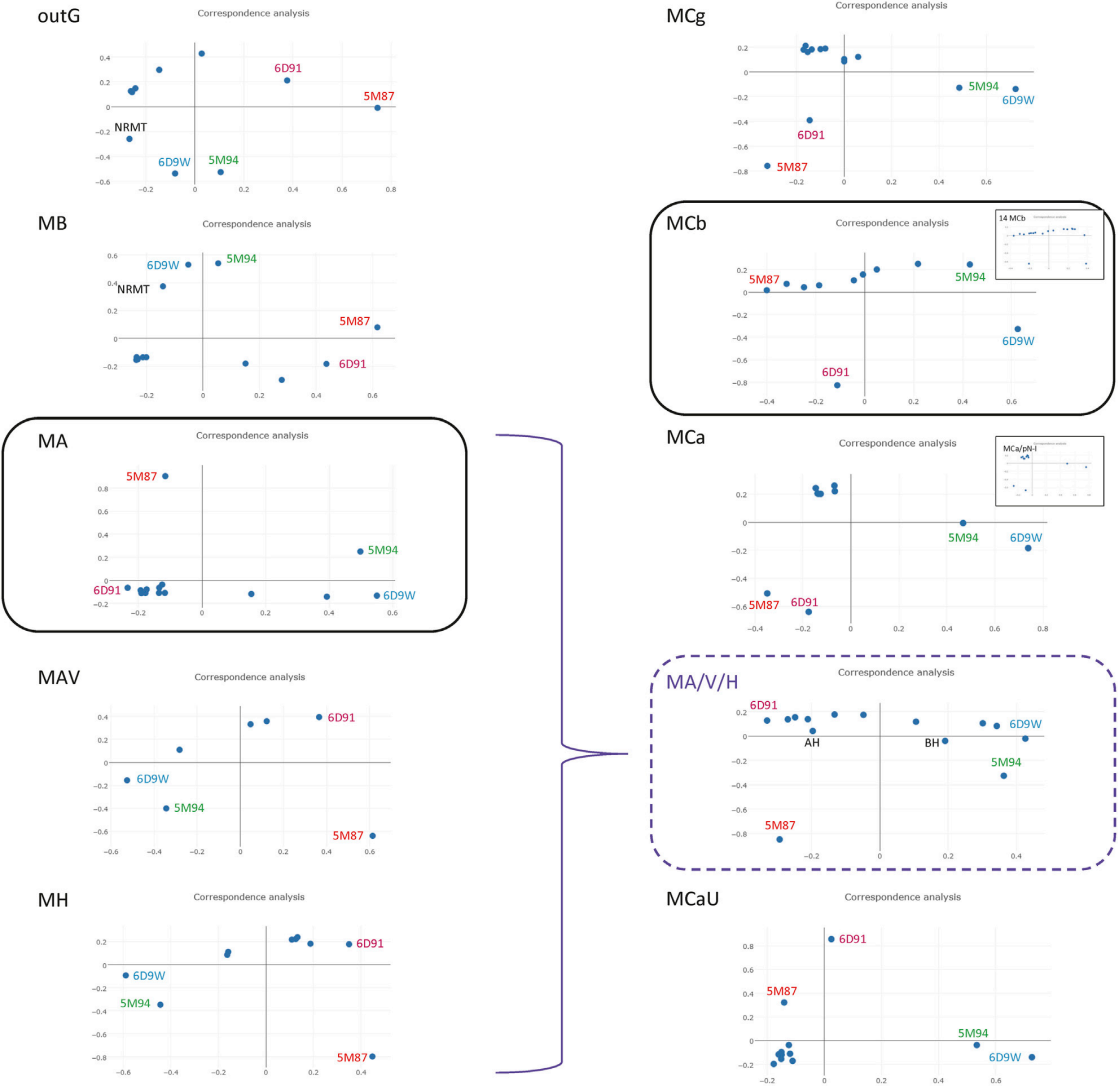
Correspondence analysis of AF2 conformers predicted in individual Slc11 phylogroups. A multidimensional scaling method was used to position AF2 models with the most similar structural neighborhoods near each other (Holm, 2022). The known structures representing outward open and inward open conformations from phylogroups MntH A and MntH Cb served as references (6D91 and 5M87, and 6D9W and 5M94, respectively) as well as NRMT inward open template (7QIA) as indicated. Selected AF2 models from MA, MAV and MH were pooled to evaluate structural similarities across these phylogroups (highlighted in purple). *Insets*. Pooled MCa and plant pN-I (3) models; pool of 14 MCb predicted conformers.

To investigate functional correlates of MntH phylogroups the conformational structure of Alphafold (AF) models predicted for seqs of bacterial descent (MB, MA, MH) or eukaryotic origin (MCs) were compared. The Alphafold (AF) database provides the opportunity to appreciate the conformational space of virtually any protein family (Jumper et al., 2021). Regarding the Slc11 family, 3D templates representing outward open and inward open states for both MA and MCb homologs (6D91&6D9W and 5M87&5M94, respectively) were available for AF learning steps. To compare conformers predicted per MntH phylogroup the 3D model generated for each sequence (or their Uniprot proxy) used in Slc11 family tree (Figure 2) were retrieved. All against all Dali comparisons (Holm, 2022) included the MA and MCb pairs of opposite conformations as reference points (Figure 5).

AF2 models for Slc11 outgroup resemble more NRMT inward open structure than MntH templates and suggest candidate conformational intermediates toward a hypothetical NRMT outward open state. MB predicted structures appear more distant from NRMT and closer to MntH A templates, in agreement with Slc11 family tree. MA conformers roughly sit along the path from 6D91 to 6D9W with a large majority near to outward open state. Both MAV and MH conformers behave likewise. A pooled selection of MA-MAV-MH models distributes along the 6D91 to 6D9W path, placing MH models on the margin. These 3D data support close relationship of MA and MAV with MH that is consistent with Slc11 phylogeny.

In contrast, AF2 models for MCg, MCa and MCaU seqs appear as similar clusters of remote outliers while MCb conformers populate a hypothetical path linking 5M87 to 5M94 rather evenly. Plant pN-Is (Figure 2) behave similarly to MCa seqs (inset). 14 MCb seqs demonstrate an array of 3D models related to either 5M87 or 5M94 (inset). That MCb, MCg and MCa seqs yield different AF2 models despite their common origin (Supplementary Figure S6) suggests that divergent mutations among subgroups may frustrate default AF2 modelling: as a result, MCg and MCa seqs would lack MCb modelling fluidity and produce instead stereotypical models.

AF2 3D modelling of MntH seqs shows striking differences depending on their phylogenetic origin -either bacterial, such as MA, MAV and MH, or eukaryotic (MC subgroups)- which are consistent with *mntH* polyphyly (Cellier et al., 2001) and Slc11 family tree. The current AF2 models support functional diversification of MC subgroups (Cellier, 2012a) and suggest that conformational flexibility may contribute to this process, and perhaps modulate H^+^-driven Mn^2+^ pumping efficacy.

### Possible origins and consequences of Slc11 function

The reason why bacteria would select H^+^-driven Mn^2+^ uptake before onset of earth aerobiosis remains however obscure. In an anaerobic world, Mn requirements seem negligible for metabolic purposes as well as the need for antioxidant defenses. Yet transient Mn accumulation at the expense of Mg entry may be beneficial to resist adverse conditions for growth.

Recovery from severe stress (e.g., heat shock, hydrostatic pressure, osmotic stress) involves the functional switch of the Mg/Mn-dependent AMPylator (YdiU), which regulates redox homeostasis in normal conditions, into a Mn-dependent UMPylator. As recovery from stressful conditions depletes ATP stores YdiU-dependent UMPylation turns off chaperone activity to conserve cellular energy and favor cell persistence (Yang et al., 2020). Magnesium efflux may be driven by reduction of ATP levels induced in conditions that concomitantly increase Mn uptake (Yousuf et al., 2020) or by osmotic stress (Wendel et al., 2022). Transient accumulation of Mn may displace enzymatic Mg-cofactors (Foster et al., 2022; Hohle and O'Brian, 2014) and increase bacterial resistance to severe stress, which would confer a survival advantage.

Selective Mn import may also favor host cell survival upon viral infection. Several examples of bacterial antiphage systems demonstrate signaling or effector activities that are specifically Mn-dependent (Rostol et al., 2021) and some have homologs in eukaryotic cells (Tal et al., 2021). Examples of human viruses that encode intrinsic Mn specificity in enzymatic activities that are key for their replicative cycle (Vogel et al., 2019; Wang et al., 2021) further suggest that intracellular Mn is a cue during viral infection. Because bacteriophages infect anaerobic bacteria as well, resistance to viral infection represents another possible reason for selecting Slc11 specific function.

As increased intracellular Mn/Mg ratio allows functioning of Mn-dependent activities that sustain bacterial resistance to severe stress even in anaerobiosis (e.g., heat shock, osmotic stress, viral infection), it is speculated that (Slc11) MB emerged to meet the need for a selective Mn import pathway enabling rapid (Yousuf et al., 2020), transient (Ouyang et al., 2022) accumulation of Mn, perhaps coordinated with Mg export, in response to severe stress conditions.

**FIGURE 6:**
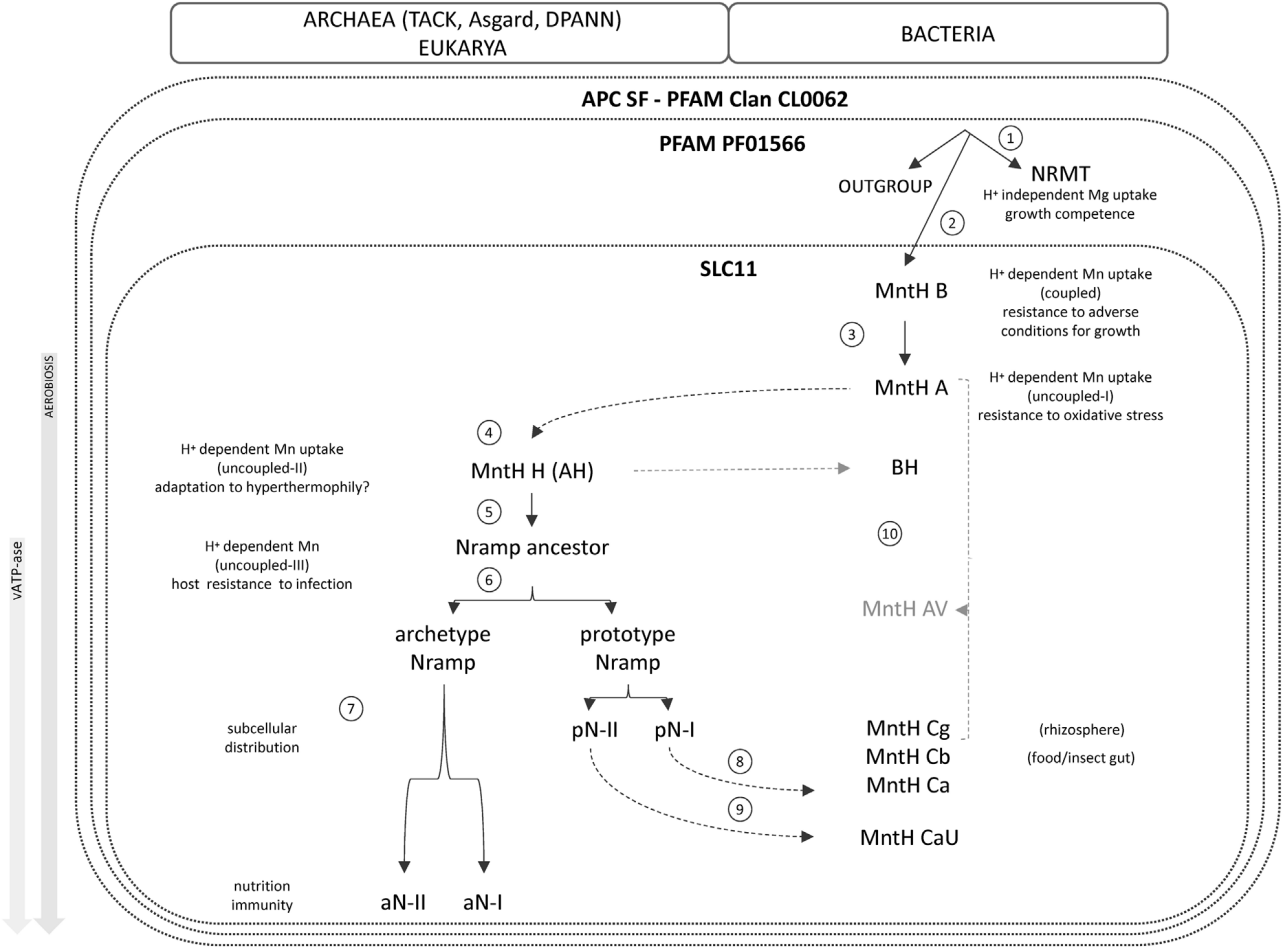
Proposed evolution of the Slc11 family.

The molecular logic of Slc11 carrier may thus be viewed as a stress resistance function sustaining cell persistence (Figure 6). H^+^-dependent Mn^2+^ import catalyzed by MB could serve some “elemental chaperone” purpose in anaerobiosis, because Mn can substitute for both Mg^2+^ and ATP cofactors to prolong cell stress resistance when cellular energy is depleted. Mn accumulation might as well help anaerobes coping with episodic exposure to O_2_, which prohibits photoautotrophic growth (Kanao et al., 2001; Li et al., 2009). This stress resistance concept is analogous to Mn replacement of non-redox Fe-cofactor in enzymes, which helps aerobic bacteria sustaining oxidative stress conditions (Imlay, 2014). Slc11 stress resistance function was further compounded by aerobic metabolism, as MA dependent Mn import contributes to extreme radiation resistance (Sharma et al., 2017) and nitrosative stress resistance (Yousuf et al., 2020).

MA-variant MH may have resulted from adaptation to hyperthermophily in archaea. It was subsequently co-opted to mediate antibacterial immunity in eukaryotes wherein phagocytic vacuole depletion of Mn via aNs such as Nramp1 may weaken stress resistance of bacterial invaders (Buracco et al., 2015; Peracino et al., 2006). Eukaryotic Nramp were built on stepwise evolution of the use of the protonmotive force, by edification of a complex H^+^-network in which the transition from MA to MH represents a key pre-eukaryotic step. As MH is mainly present in extant archaea related to the currently closest known relatives of FECA, it seems logical that MH activity could contribute directly to eukaryogenesis processes such as endosymbiotic events.

Though Nramp1 prevents death from fulminant infection with virulent *Salmonella enterica* serovar Typhimurium (*S.* Typhimurium) bacteria remain carried chronically in the reticuloendothelial system (RES) for as long as 1 year after infection (Monack et al., 2004). Paradoxically, limiting stress resistance of ingested bacteria by depriving the phagocytic vacuole of Mn may lead to survival of a few, competent for growth related processes requiring Mg as a co-factor (DNA and RNA synthesis, energy production and intracellular signaling), a situation analogous to reversion of starvation-induced persister formation (Xu et al., 2020) and reminiscent of the role of Mg in regrowth of quiescent cells (Metaane et al., 2022). In turn, Mg could appear rate-limiting for intravacuolar survival, which may explain why virulent *S.* Typhimurium feel starved for Mg, relying on the MgtB/MgtU importer system to survive within *Slc11a1* ^*+*^ macrophages (Groisman and Chan, 2021). Along these lines, it is proposed that archaeal MH could contribute to eukaryogenesis by favoring growth-competent bacterial endosymbionts.

## Conclusion

This work establishes a function-aware phylogenetic framework useful to decipher the molecular logic of Slc11 carriers and its evolution, from anaerobic bacteria to crown eukaryotes and backward from eukaryotes to prokaryotes through HGT.

Serial waves touching select locations directed Slc11 evolution and developed the charge network that interacts with the protonmotive force driving Mn^2+^ import. Prokaryotic MntHs populate 4 phylogroups that match discrete evolutionary steps while rounds of gene duplication/loss diversified Nramps in eukaryotes.

The underlying hypothesis, that strong conservation of Slc11 3D architecture fostered site-specific coevolution in response to environmental changes, is testable by asking for instance whether MB is a tightly coupled Mn^2+^/H^+^ importer; MH uncoupling properties are intermediate between MA and MCb; aN evolved to control microbial infection.

Evolution of antagonistic activities is common among protein families with diverse regulatory functions and an important force driving host-microbe interactions. In this context, Slc11 co-importers of Mn^2+^ and H^+^ offer a remarkable example of evolved antagonistic activity, which coincided with the eukaryogenesis process, in the transition from bacterial permease to host antibacterial defense.

Deciphering Slc11 functional evolution has broad implications regarding the tug of war between host cell and microbe. Artificial intelligence approaches (such as AF2) (Jumper et al., 2021; Mirdita et al., 2022), which can predict multiple natively folded conformations to high accuracy, will be instrumental toward this aim.

Mining sites of interaction/flexibility (Zhang et al., 2020) that can be mutated to produce, or induce AF2 modeling of, alternate conformers (Bozzi et al., 2016a; Bozzi et al., 2016b; Del Alamo et al., 2022; Stein and Mchaourab, 2022; Xiao et al., 2022) should be useful to represent the spectrum of conformational diversity in the Slc11 family, and comparative analyses across phylogroups will aid probing the dynamics of Slc11 carrier cycle and its plasticity throughout evolution.

## Methodology

Maximum Likehood (ML) (branch length- and among site variation-aware, and computationally tractable) phylogenetic analyses were performed using IQ-Tree (Trifinopoulos et al., 2016), an approach implementing the FreeRate heterogeneity model to estimate variations of substitution rate among sites, as well as proportions of site categories instead of discrete categories of rate variation imposed by the Gamma model (Kapli et al., 2020). IQ-Tree was implemented to infer phylogenies using mixture models for proteins that use site-specific a.a. frequencies resulting from different local structural constraints to account for compositional variance among sites (Le et al., 2008). Structure-aware mixture matrices increase per site informational content and thus allow better reconstruction of single gene family tree than standard protein substitution models, which are based on a fixed rate a.a. replacement matrix derived from a single large database (Kapli et al., 2021).

Phylogenetic mixture models combine several a.a. replacement matrices in attempt to better model the complex process of site evolution, which depends on factors such as genetic code, solvent exposure, secondary and tertiary structure as well as protein function. These include both unsupervised mixture models (UL2, UL3) that learn site categories empirically and knowledge-based mixture models, such as EX2 (exposed/buried) and EX3 (exposed/intermediate/buried), EHO (extended/helix/other) and EX-EHO. For each analysis the number of sequences used was adjusted to ensure that the number of free parameters (tree branches and model parameters) was inferior to the number of distinct site patterns analysed. Also, 1000 replica of each of two types of branch support calculations (ultrafast bootstrap and SH-aLRT single branch test) were routinely performed to estimate confidence in the tree clades obtained. A scale bar representing the number of substitutions per site is provided with each tree presented.

Site-specific evolutionary conservation was calculated between Nramp types using Consurf (Ashkenazy et al., 2016) and a Bayesian calculation for the rate of evolution at each site in the MSA, based on the standard substitution model that was estimated to best fit the submitted data (WAG). Organisms that retained a copy of both pN and aN were selected, with few exceptions such as Rhodophyta (Cyanidiophyceae, Florideophyceae) aN-I, too divergent (compositionally biased) to be included in the analyses (Burki et al., 2019; Kapli et al., 2020). Results were displayed onto 3D models generated using I-Tasser (Yang and Zhang, 2015), custom alignment of sequence query with target 3D templates, i.e., structures of bacterial MCb in either outward facing (EcoDMT, 5M87) (Ehrnstorfer et al., 2017) or inward open (ScaDMT, 5M94) (Ehrnstorfer et al., 2014) conformations, using DdiAMBaNR as query. Pairwise and multiple flexible structural superimpositions were performed using Fatcat (Ye and Godzik, 2004) and Posa (Li et al., 2014).

To predict sites of potential functional divergence between Slc11 parologs, complementary approaches were tested: Diverge 3.0 (Gu et al., 2013) and a likelihood ratio test for evolutionary rate shifts and functional divergence among proteins (Knudsen and Miyamoto, 2001). Three types of divergent sites were analyzed: type I rate shift sites, where residues are conserved in one group but vary in the other group, reflecting “heterotachy” due to altered structural constraints; type II rate-shift sites, where residues are conserved in each group but differ radically between groups, because of different physicochemical properties for instance, and type I/II sites with mixed properties. Sites displaying significant scores using both approaches were mapped onto DdiAMBaNR 3D models. Prediction of functional sites based on Slc11 phylogeny were implemented using the machine-learning approach Multi-RELIEF, which weights group-specific composition, group-specific conservation and divergence between groups (Brandt et al., 2010) as well as covariation networks analyses that emphasize local coevolutionary constraints (Fonseca et al., 2021) based on MCb 3D structures 5M87 and 5M94.

Taxonomic distribution of MntH groups: Starting with 665 manually curated a.a. sequences representing known MntH groups and their phylogenetic outgroup (Cellier, 2012a), group-specific logos covering six segments of Slc11 sequence (parts of TMS1, 3, 4, 6, 7 and 8) were generated with Weblogo (Crooks et al., 2004). Group-specific logos were combined into a single pattern (PROSITE verbose) that was uploaded to search NCBI Microbial Protein Database using Pattern Hit Initiated (PHI)-Blast (Zhang et al., 1998). Each group-specific output was aligned and used as seed multiple alignment (MSA-I) for HMMer search of database NR90 (NCBI NR clustered at 90% identity), without MSA enrichment through HHBlits, and using e^−60^ as e-value cutoff (Zimmermann et al., 2018). Group-specific HMMer outputs were forwarded to ClustalO to generate MSA-IIs that were curated to eliminate duplicates and outliers and to produce logos of second generation. The resulting logos assembled into patterns were used for new rounds of group-specific PHI-Blast searches to update MntH phylogroup compositions and establish contemporaneous taxonomic distributions.

Survey of eukaryotic Nramp: Prototype (pN-I and pN-II) and archetype Nramp (aN-I and aN-II) were searched using PSI-BlastP (Zhang et al., 1998) and TBlastN approaches applied to NCBI NR databases. TBlastN searches of NCBI Transcriptome Shotgun Assembly (TSA) and (SRA) repositories were performed to seek additional pN and aN-I sequences, in particular. Transcript short fragments identified in SRA were assembled into full-length sequences by walking, ensuring at least 2x coverage from independent fragments. Extra caution was exercised to characterize pN homologs, as they may be confounded with prokaryotic MCs. Candidate pNs were routinely tested using BlastN approach against NR database to eliminate bacterial contaminants. Sequences which appeared extremely divergent (compositionally biased) were excluded from analyses (Burki et al., 2019; Kapli et al., 2020).

## Limitations of the study

The outgroup used in this study comprises the sequences most closely related to Slc11 carriers but lacking the specific features required for Mn and proton transport. This outgroup is related to but distinct from NRMT group. The 3 clades (Slc11 family, this outgroup and sister NRMT) form the PF01566 family. Both Slc11 phylogeny and Slc11-specific collection of evolutionary rate-shifts were resilient to outgroup diversification using NRMT sequences (Supplementary Appendix S4, S5), implying the proposed phylogenetic framework reflects functional properties intrinsic to the Slc11 family. Stochastic errors, inherent to sequence length, were observed in Slc11 family tree (Figure 2) despite using a free-rate ML approach and mixture matrices: tunicate Nramp grouping with vertebrate Nramp2, and paraphyly of pN-I and pN-II. Additional analyses clarified relationships among vertebrate aN-II (not shown) and between pN-I and pN-II (Figure 1; Supplementary Figures S3–S6). Systematic error such as long branch attraction artefact due to compositional bias (incorrect grouping of highly divergent sequences) likely explain the branching pattern of MntH most deviating sequences (cf Supplementary Figures S3–S7).

## Data Availability

The original contributions presented in the study are included in the article/Supplementary Material, further inquiries can be directed to the corresponding
