# Supplementary material for: Nramp: Deprive and conquer?
Source: Front Cell Dev Biol. 2022 Oct 13;10:988866. doi: 10.3389/fcell.2022.988866 (PMC9606685; doi:10.3389/fcell.2022.988866)
Supplement: Supplementary file 1 [file DataSheet2.pdf]

Appendix1: MA (6D91) and MCb (5M87) outward open 3D structures inserted into simulated lipid bilayers

6D91

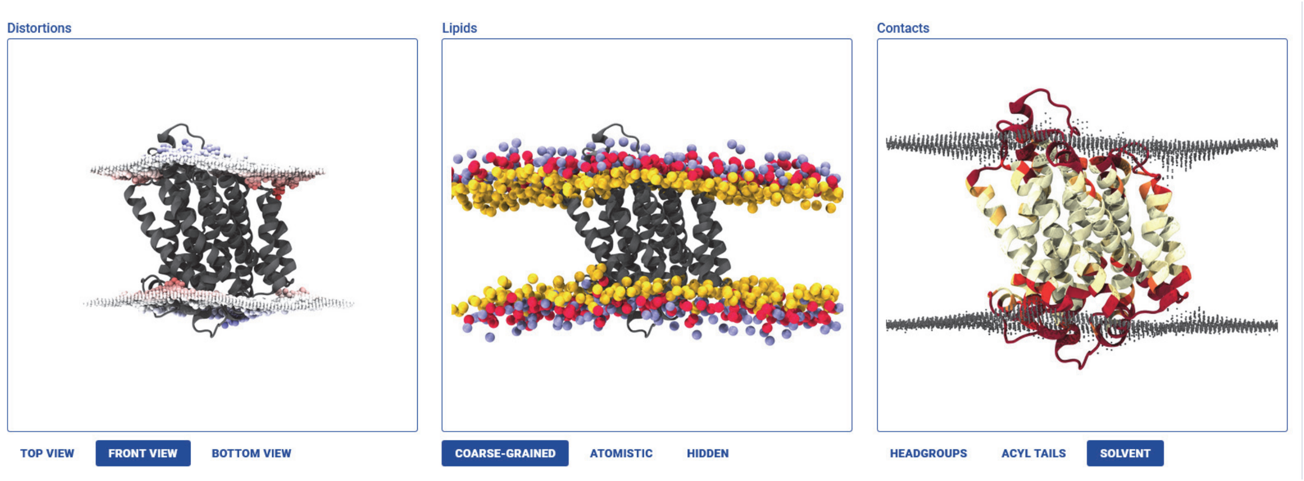

http://memprotmd.bioch.ox.ac.uk/_ref/PDB/6d91/_sim/6d91_default_dppc/

5M87

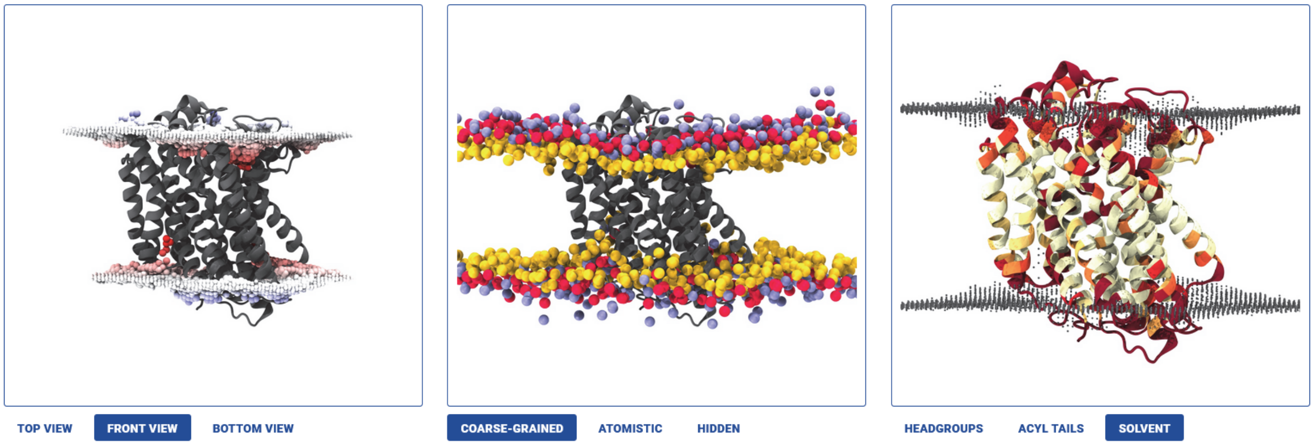

Distortions show the average surface formed by lipid phosphate beads over the final 800 ns of simulation time. Red indicates a thinning of the bilayer (compared to bulk thickness), whilst blue indicates thickening.

Coarse-grained lipids are shown with glycerol beads in yellow, phosphate beads in red and choline beads in blue. Atomistic lipids are coloured according to the CPK standard.

Contacts show the average occupancy of the selected group within 6 Å of the protein over the final 800 ns of simulation time. Use the buttons below to select a representation.

http://memprotmd.bioch.ox.ac.uk/_ref/PDB/5m87/_sim/5m87_default_dppc/

Newport TD, Sansom MSP, Stansfeld PJ. 2019. The MemProtMD database: a resource for membrane-embedded protein structures and their lipid interactions. *Nucleic Acids Res*. 47: D390-D397

Appendix2: Phylotyping *Rhodelphis* aN-I.

Phylogenomic characterization of *Rhodelphis* spp. established the novel eukaryotic phylum, Rhodelphidia, as a sister group to red algae (Rhodophyta) comprising flagellated predators, which maintained phagotrophic feeding as part of the Archaeplastida super group (Viridiplantae, Glaucocystophyceae, Rhodophyta; Gawryluk, Tikhonenkov et al., 2019).

To determine which type(s) of Slc11 are expressed by *R. limneticus*, the strand specified HiSeq transcriptome of manually picked cells (SRX5960833) was queried with various a.a. sequences in TBlastN analyses. The left panel of the accompanying Figure (p. 3) shows the Glaucocystophyceae aN-I sequence (CpaGLA_aN-I) produced more best hits.

A single *R. limneticus* full-length mRNA sequence was obtained by assembling non-ambiguous, overlapping sequence reads (2-3X) and the resulting translated a.a. sequence was used to query SRX5960833. The right panel of the figure p. 3 shows it is the sole Slc11 homolog present in this SRA (top), which was phylo-typed as aN-I (RliRHOPeNR, bottom, and based on 11 aN-I vs 3 aN-II conserved diagnostic sites, not shown).

In addition, searching *R. marinus* transcriptome (SRX5960835) identified the ortholog of *R. limneticus* aN-I (610 bits, 68% sequence id, essentially over the 10 TMS (5+5) hydrophobic core that typifies Slc11 carriers). RmaRHOPeNR is also the sole Slc11 homolog found in this organism. Hence *Rhodelphis* data strengthen the link between archetype Nramp (aN) and phagocytosis.

Note: The 80% identity cutoff used to discard nucleotide sequences from the preys of *R. limneticus* or *R. marinus* from mixed culture data (Gawryluk, Tikhonenkov et al., 2019) imply that *pN* fragments would have remained accessible for Slc11 Blast searches, should *Rhodelphis* spp. possess such genes.

Gawryluk RMR, Tikhonenkov DV, Hehenberger E, Husnik F, Mylnikov AP, Keeling PJ. 2019. Non-photosynthetic predators are sister to red algae. *Nature*. 572:240-243

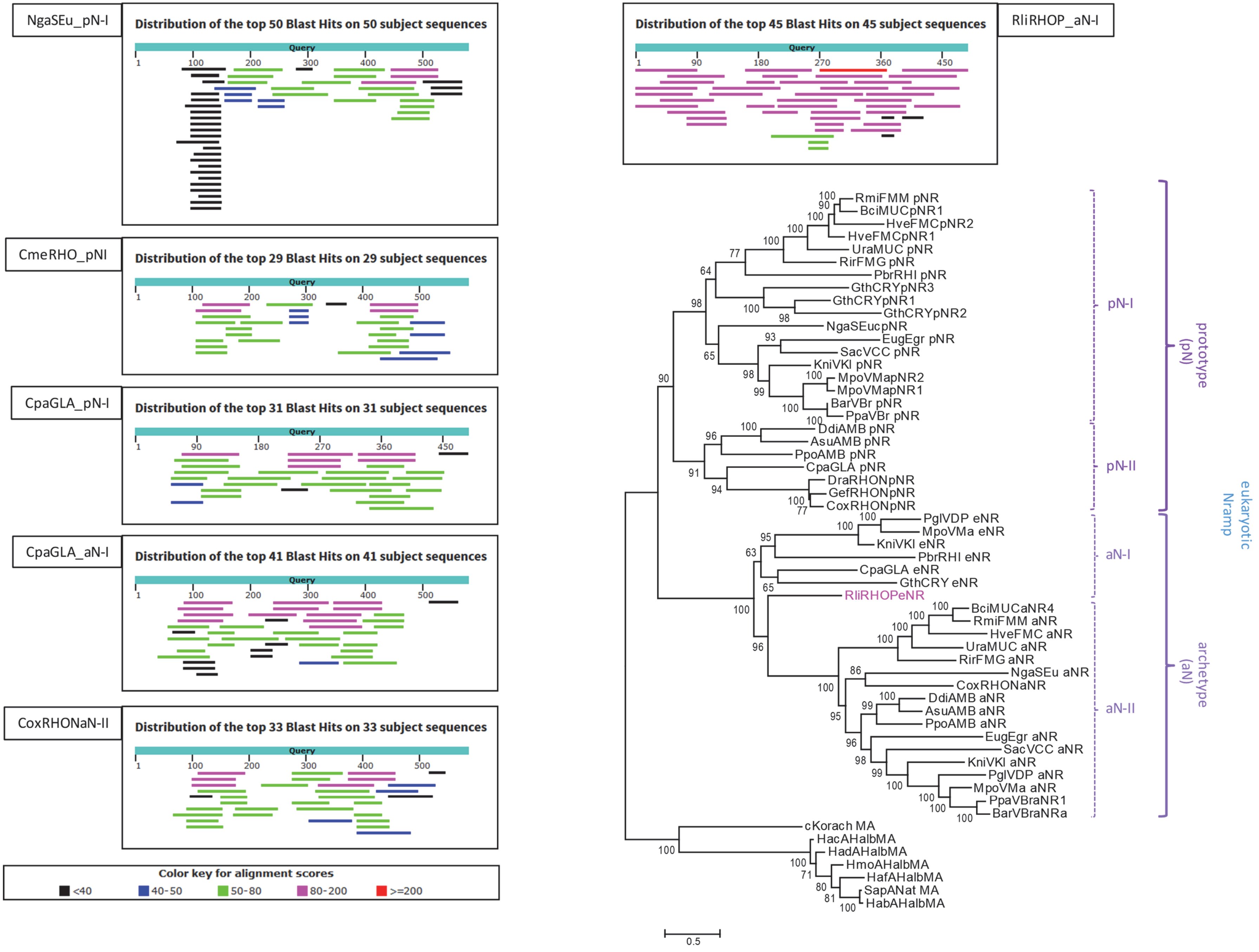

Appendix 2-Figure

Appendix3: *MA* gene displacement by *MCb* xenolog in GPB lineages of animal symbionts

The presence of a *MCb* gene in *Wigglesworthia glossinidia*, a GPB living as obligate endosymbiont, suggested that HGT of *MCb* and endogenous *MA* deletion preceded *W. glossinidia* endosymbiotic process (Akman, Yamashita et al., 2002, Richer, Courville et al., 2003). To examine such processes further, genomes of known GPB symbionts were mined for *mntH* genes and phylogenies of the proteins encoded in symbionts and free-living close relatives were inferred and compared with the taxonomy of the respective organisms (cf accompanying table p. 6 and figure p. 7).

The results show partition of two main groups of symbionts, carrying either a *MA* or a *MCb* gene, plus a few species with both genes on their chromosome; arguably, such species represent an evolutionary snapshot preceding *MA* gene replacement by *MCb* xenolog. In both MA and MCb clades, increased sequence divergence generally correlates with significant reduction in %GC (and/or size) of the encoding genome. The existence of either *MA* or *MCb* in related endosymbionts with drastically reduced genomes (e.g., respectively *Blochmannia* spp. and *Baumannia* spp., *Wigglesworthia* spp.) suggest that selective gene maintenance may depend on host-microbe interactions.

MA positive symbionts apparently derive from several ancestors that belong to the Vibrionales and Enterobacterales orders, including members of the *Enterobacteriaceae*, *Bruguierivoracaceae* and *Erwiniaceae* families for the latter. Within each order MA sequences are highly related to each other. Regarding *Blochmannia* spp., MA relationships may reflect the time elapsed since association with their respective host (Williams and Wernegreen, 2015, Ward, Blaimer et al., 2016), i.e. time spent since the divergence of ants species (about 70 mya for the *Formicini-Camponotini* split, Moreau, Bell et al., 2006).

MCb positive symbionts are taxonomically more diverse, including spp. from the GBP orders Enterobacterales, Orbales, Pasteurellales and Moraxellales, as well as one BPB order (Neisseriales). Proteobacterial MCbs form a monophyletic clade, distantly related to MCbs from Firmicutes (Lactobacillales), and more conserved than MA clade: MCbs from different orders are similarity related than MAs within a given order.

These findings are consistent with vertical transmission of *MA* in GPB and horizontal dissemination of *MCb* in spp. that live in association/symbiosis with select insect hosts. The relative prevalence of *MCb* in genomes of bacteria populating the gut of pollinating insects suggests this milieu constitutes an ecological niche conducive for HGT of Lactobacillales' *MCb*.

Species possessing both *MA* and *MCb* genes are closely related *Sodalis*-allied symbionts (*Enterobacteriaceae* and/or *Bruguierivoracaceae*). The corresponding sequences are highly related to each other within each clade (MA or MCb). *Sodalis*-allied symbionts comprise free-living (Chari, Oakeson et al., 2015), facultative (Meseguer, Manzano-Marín et

al., 2017, Sloan & Moran, 2012) and obligate (Clayton, Jackson et al., 2016) endosymbionts, which suggest current association with their host that is supported by high %GC indicating limited genome decay.

Only free-living and facultative endosymbiont *Sodalis* spp. possess both full-length *MA* and *MCb* genes (Chari, Oakeson et al., 2015, Sloan & Moran, 2012). Maintenance of both genes in *S.* sp. *SoCistrobi* genome, despite 40% reduction, suggest some benefit for facultative association with the white pine aphid (Meseguer, Manzano et al., 2017). Inversely, selective inactivation of both genes in *S. glossinidius* (Toh, Weiss et al., 2006) implies neither is required for facultative association with the tsetse fly – though a sitABCD system may be used to import Mn. These data demonstrate HGT of *MCb* in *Sodalis* preceded endosymbiotic associations.

1-Akman L, Yamashita A, Watanabe H, Oshima K, Shiba T, Hattori M, Aksoy S. 2002. Genome sequence of the endocellular obligate symbiont of tsetse flies, Wigglesworthia glossinidia. *Nat Genet*. 32:402-7

2-Chari A, Oakeson KF, Enomoto S, Jackson DG, Fisher MA, Dale C. 2015. Phenotypic characterization of Sodalis praecaptivus sp. nov., a close non-insect-associated member of the Sodalis-allied lineage of insect endosymbionts. *Int J Syst Evol Microbiol*. 65:1400-1405

3-Clayton AL, Jackson DG, Weiss RB, Dale C. 2016. Adaptation by Deletogenic Replication Slippage in a Nascent Symbiont. *Mol Biol Evol*. 33:1957-66

4-Meseguer AS, Manzano-Marín A, Coeur d'Acier A, Clamens AL, Godefroid M, Jousselin E. 2017. Buchnera has changed flatmate but the repeated replacement of co-obligate symbionts is not associated with the ecological expansions of their aphid hosts. *Mol Ecol*. 26:2363-2378

5-Moreau CS, Bell CD, Vila R, Archibald SB, Pierce NE. 2006. Phylogeny of the ants: diversification in the age of angiosperms. *Science*. 312:101-4

6-Richer E, Courville P, Bergevin I, Cellier MF. Horizontal gene transfer of "prototype" Nramp in bacteria. 2003. *J Mol Evol*. 57:363-76

7-Sloan DB, Moran NA. 2012. Genome reduction and co-evolution between the primary and secondary bacterial symbionts of psyllids. *Mol Biol Evol*. 29:3781-92

8-Toh H, Weiss BL, Perkin SA, Yamashita A, Oshima K, Hattori M, Aksoy S. 2006. Massive genome erosion and functional adaptations provide insights into the symbiotic lifestyle of Sodalis glossinidius in the tsetse host. *Genome Res*. 16:149-56.

9-Ward PS, Blaimer BB, Fisher BL. 2016. A revised phylogenetic classification of the ant subfamily Formicinae (Hymenoptera: Formicidae), with resurrection of the genera Colobopsis and Dinomyrmex. *Zootaxa*. 4072:343-57

10- Williams LE, Wernegreen JJ. 2015. Genome evolution in an ancient bacteria-ant symbiosis: parallel gene loss among Blochmannia spanning the origin of the ant tribe Camponotini. *PeerJ*. 3: e881

Appendix 3-Table: *mntH* gene complement in GPB symbionts and related spp.

Species	Class\Family	Symbiosis*	genome\GC % size Mb	MA$	MCb#
Blochmannia turneri	GPB\Enterobacteriaceae	P endo (*Camponotini*)	0.75\29.1	cBvaGPB MA	
B. floridanus	GPB\Enterobacteriaceae	P endo (*Camponotini*)	0.71\27.4	cBflGPB MA	
B. vafer	GPB\Enterobacteriaceae	P endo (*Camponotini*)	0.72\27.5	cBchGPB MA	
B. turneri	GPB\Enterobacteriaceae	P endo (*Camponotini*)	0.75\29.1	BePtGPB MA	
B. obliquus	GPB\Enterobacteriaceae	P endo (*Colobopsis*)	0.77\27.4	BeCoGPB MA	
Westeberhardia cardiocondylae	GPB\Enterobacteriaceae	P endo (*Formici*)	0.53\23.4	ceCoGPB MA	
secondary endosymbiont of *C.eucalypti*	GPB\Enterobacteriaceae	S endo (*Ctenarytaina eucalypti*)	1.4 \43	seCeGPB MA	
Sodalis praecaptivus	GPB\Bruguierivoracaceae	F-L human wound	5.16\57.1	Spr GPB MA	SprGPB MCb
S. endosymbiont of H. halophilus	GPB\Bruguierivoracaceae	P endo (*Henestaris halophilus*)	1.62\36.8		SeHhGPBMCb
S. pierantonius str. SOPE	GPB\Bruguierivoracaceae	P endo (*Sitophilus oryzae*)	4.51\56.1	cSpiGPB MA	cSpiGPBMCb#
S. sp. SoCistrobi	GPB\Bruguierivoracaceae	S (*Cinara strobi*)	3.08\56.4	SsSoGPB MA	SsSoGPBMCb
S. glossinidius	GPB\Bruguierivoracaceae	S endo (*Glossina brevipalpis*)	4.29\54.4	Sgl GPB MA$	SglGPB MCb#
S.-like symbiont of *P. spumarius*	GPB\Bruguierivoracaceae	P endo (*Philaenus spumarius*)	1.81\53.8		SsPsGPBMCb
Doolittlea endobia	GPB\Enterobacteriaceae	endo (*Maconellicoccus hirsutus*)	0.85\44.2		cDenGPBMCb
Hoaglandella endobia	GPB\Enterobacteriaceae	endo (*Tryonimus perrisii*)	0.64\42.7		cHenGPBMCb
Baumannia cicadellinicola	GPB\ Baumannia	endo (*Graphocephala atropunctata*)	0.67\33		Bci1GPBMCb
B. cicadellinicola	GPB\ Baumannia	endo (*Draeculacephala minerva*)	0.64\31		Bci2GPBMCb
B. cicadellinicola	GPB\ Baumannia	endo (*Homalodisca coagulata*)	0.69\33.1		Bci3GPBMCb
symbiont of *H. cubana*	GPB\Enterobacteriaceae	S endo (*Heteropsylla cubana*)	1.1 \28.5		seHcGPBMCb
Citrobacter freundii	GPB\Enterobacteriaceae	gut (*Bactrocera dorsalis*)	5.26\51.7	Cfr GPB MA	
Salmonella bongori	GPB\Enterobacteriaceae	(reptiles)	4.45\51.3	SbonGPB MA	
A symbiont of *P. stali*	GPB\Enterobacteriaceae	P midgut (*Plautia stali*)	3.85\57.1	sPs GPB MA	
E symbiont of *P. stali*	GPB\Enterobacteriaceae	equivalent to A (*Plautia stali*)	5.39\53.7	EsPsGPB MA	
Tachikawaea gelatinosa	GPB\Enterobacteriaceae	P midgut (*Urostylis westwoodii*)	0.71\25.1	cTgeGPB MA	
Wigglesworthia glossinidia	GPB\Erwiniaceae	P endo (*Glossina morsitans*)	0.70\22.5		Wgl1GPBMCb
W. glossinidia	GPB\Erwiniaceae	P endo (*Glossina morsitans*)	0.72\25.2		Wgl2GPBMCb
Bacterium symbiont	GPB\unclass	endo (*Pedicinus badii*)	0.56\~24		bePbGBPMCb
Pantoea carbekii	GPB\Erwiniaceae	P extracell. (*Halyomorpha halys*)	1.2 \30.5	cPcaGPB MA	
P. ananatis	GPB\Erwiniaceae	broad host range plant path.	4.9 \53.5	Pan GPB MA	
Erwinia amylovora	GPB\Erwiniaceae	causative agent of Fire Blight	3.8 \53.5	Eam GPB MA	
E. toletana	GPB\Erwiniaceae	olive plant epiphyte	5.3 \53.5	Eto GPB MA	
first bacterial symbiont of *F. occidentalis*	GPB\Erwiniaceae	P (*Frankliniella occidentalis*)	~5 \~52	bsBF1GPBMA	
second bacterial symbiont of *F. occidentalis*	unclassified bacterium	P (*Frankliniella occidentalis*)	~3 \~46	bsBF2GPBMA	
Tatumella citrea	GPB\Erwiniaceae	F-L	4.5 \49.5	Tci GPB MA	
Morganella morganii	GPB\Morganellaceae	human GI	3.97\51		MmoGPB MCb
Gilliamella apicola	GPB\Orbaceae	gut (*Apis mellifera*)	2.85\34.4		GaplGPBMCb
G. apis	GPB\Orbaceae	gut (*Apis/Bombus*)	2.49\34.7		GapsGPBMCb
Pasteurella skyensis	GPB\Pasteurellaceae	fish path (*Salmo salar*)	2.22\34.9		PskGPB MCb
Phocoenobacter uteri	GPB\ Pasteurellaceae	uterus (*Phocoena phocoena*)	4.64\38.5		PhuGPB MCb
Vibrio harveyi	GPB\Vibrionaceae	F-L tropical marine environt	5.85\44.9	Vha GPB MA	
Photobacterium leiognathi	GPB\Vibrionaceae	fish bioluminescent symbiont	4.63\41.1	PlemGPB MA	
Enterovibrio escacola	GPB\Vibrionaceae	biolum. (Melanocetus *johnsonii*)	2.73\39.8	bsMjGPB MA	
Acinetobacter apis	GPB\Moraxellaceae	gut flora (*Apis*)	2.41\38.3		AapGPB MCb
A. boissieri	GPB\Moraxellaceae	floral nectar and bees (*Apis*)	2.69\38		AboGPB MCb
A. nectaris	GPB\Moraxellaceae	floral nectar and bees (*Apis*)	2.67\36.7		AneGPB MCb
Snodgrassella alvi	BPB\Neisseriaceae	gut (*Apis mellifera*)	2.5 \41		SalBPB MCb
Lactobacillus reuteri	Bacilli\Lactobacillaceae	human gastro-intestinal mucosa	2.15\38.5		LreFMC MCb
L. plantarum WJL	Bacilli\Lactobacillaceae	various ecological niches	3.23\44.6		LplFMC MCb

***P, primary (obligate); S, secondary (facultative); F-L, free-living** **$,#, pseudogenes**

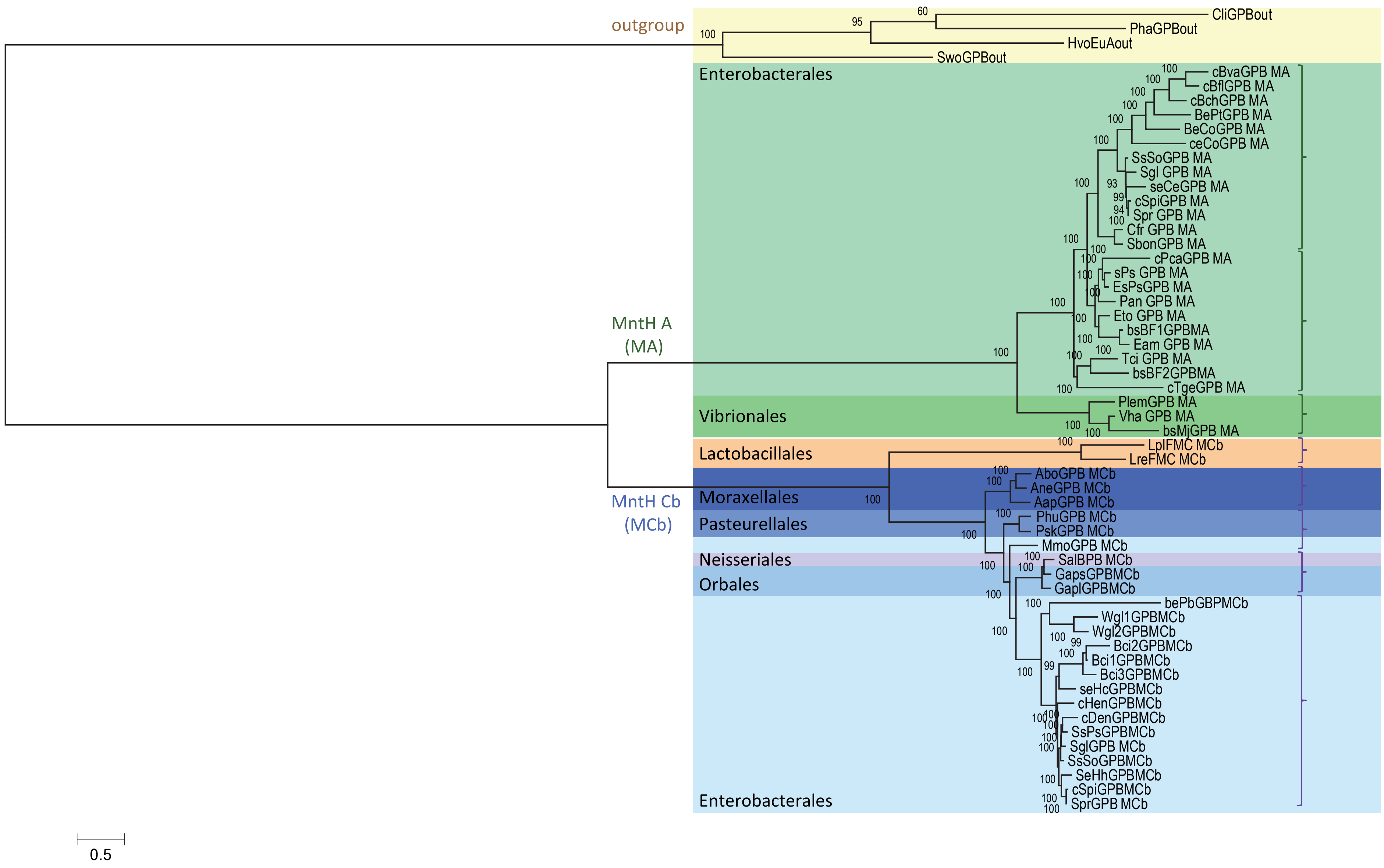

Appendix 3-Figure: Phylogeny of MA and MCb proteins derived from GPG symbionts and related spp.

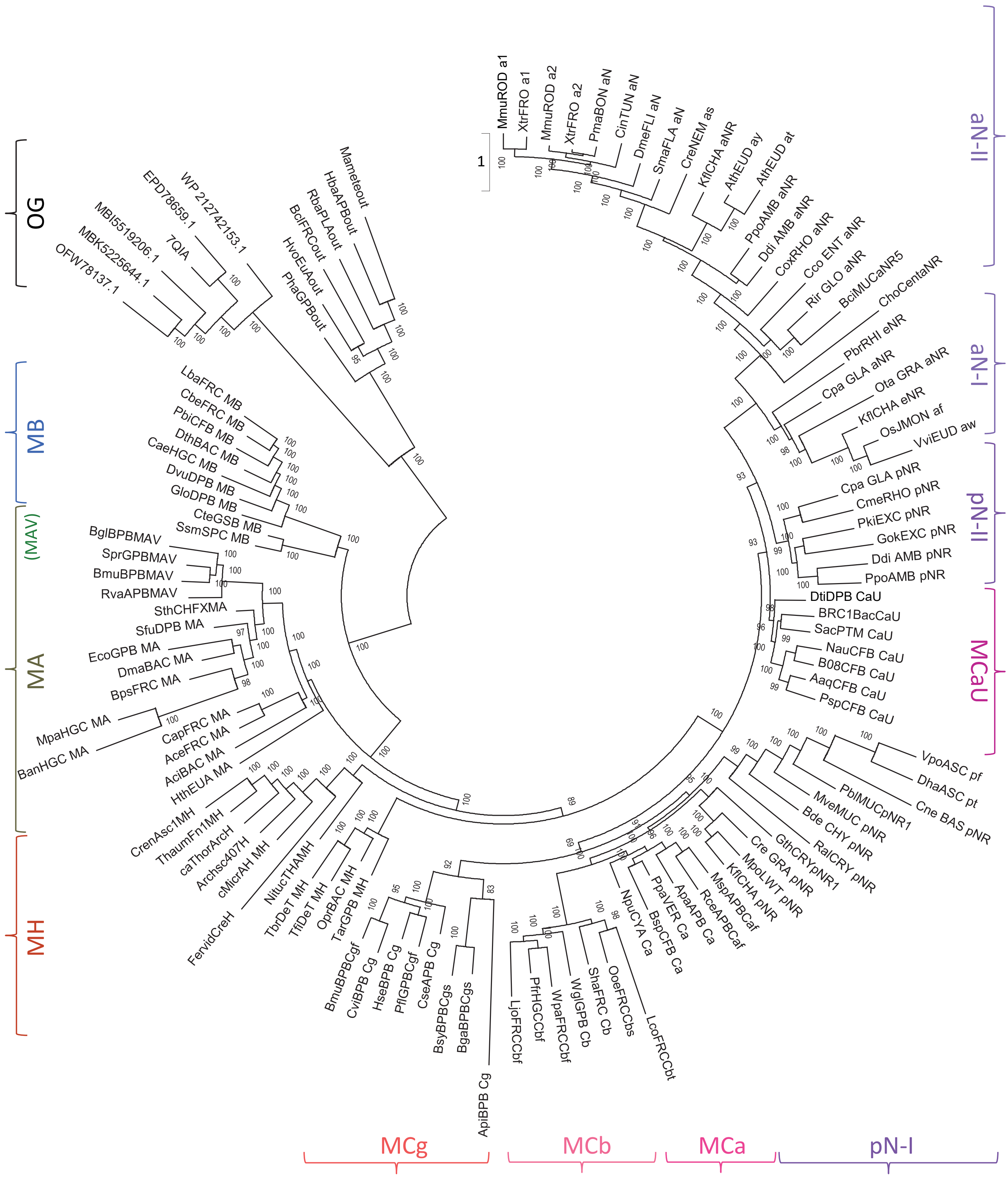

Appendix 4: Slc11 phylogeny using NRMT-extended outgroup (cf Figure 2)

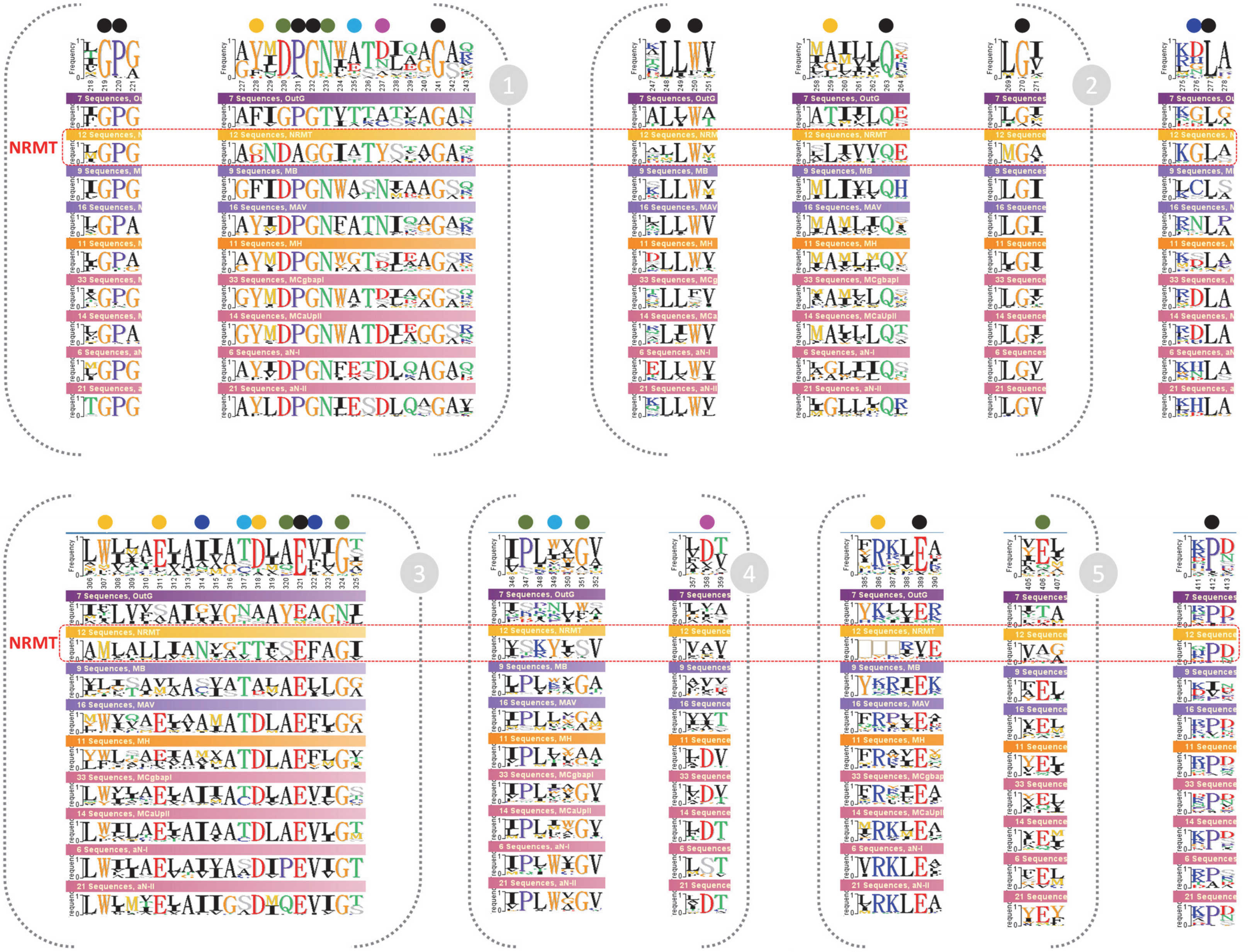

Appendix 5 Sequence logos including NRMT outgroup showing the conserved sites highlighted in Figure 4 (cf Figure S9)

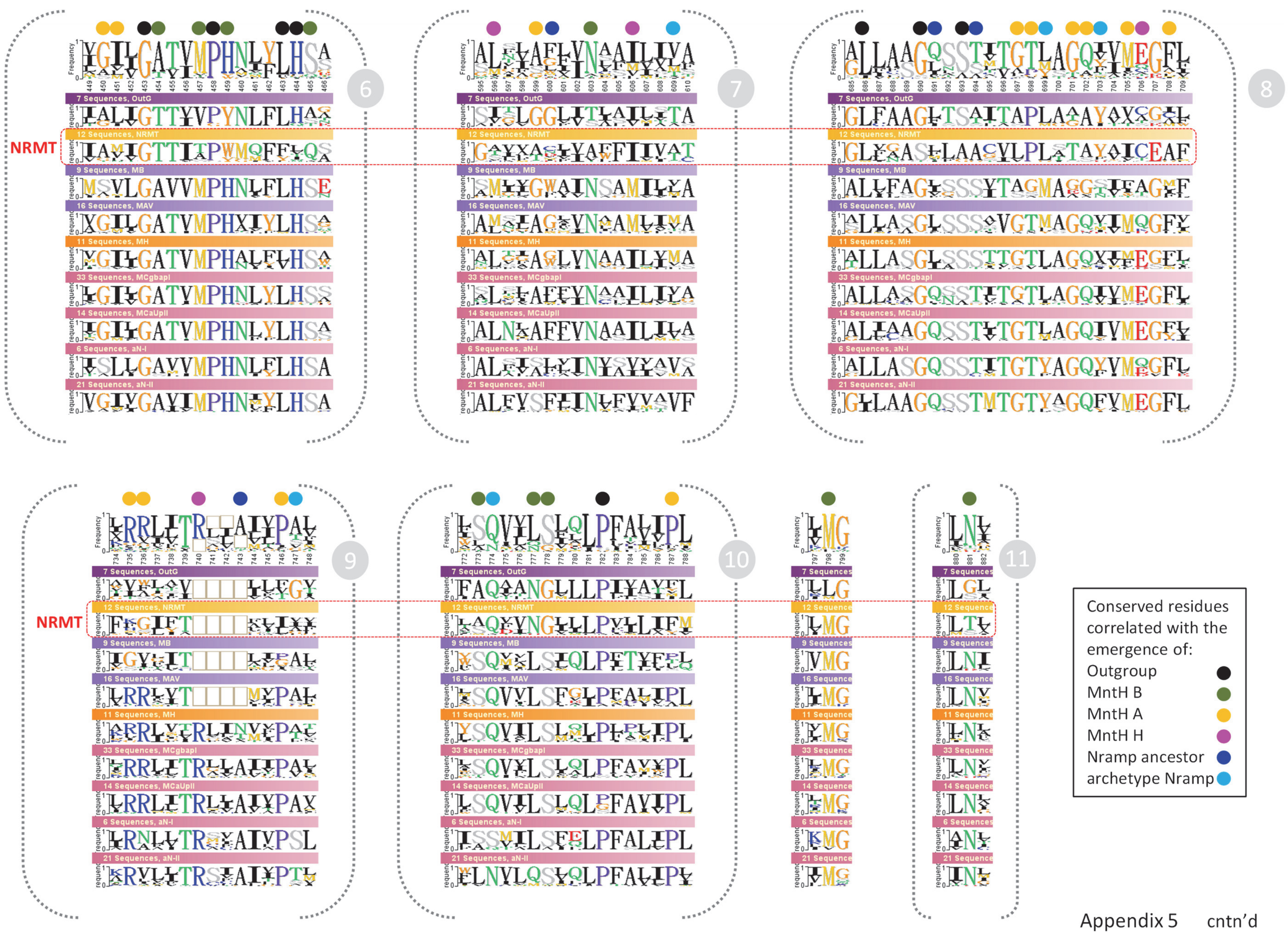

Appendix 5 cntn'd

Appendix 6: Details of sequences used in Figure 1: prototype Nramp (pN) and archetype Nramp (aN)

pN-I:

**HveFMCpNR1**	
natural resistance-associated macrophage protein [Hesseltinella vesiculosa]	Fungi\Mucoromycota\Cunninghamellaceae
ACCESSION ORX48876	
**HveFMCpNR2**	
natural resistance-associated macrophage protein [Hesseltinella vesiculosa]	Fungi\Mucoromycota\Cunninghamellaceae
ACCESSION ORX57495	
**RmiFMM_pNR**	
natural resistance-associated macrophage protein [Rhizopus microsporus ATCC 52813]	Fungi\Mucoromycota\Mucorineae
ACCESSION XP_023468363	
**BciFMMpNR1**	
jgi|Bacci1|237824|estExt_Genewise1Plus C_60434 [Backusella circina FSU 941 v1 0]	Fungi\Mucoromycota\Mucorineae
https://genome.jgi.doe.gov/mycocosm/home	
**UraFMU_pNR**	
jgi|Umbra1|230632|fgenesh1_kg 13_#_154_#_combest_scaffold_13_17261 [Umbelopsis ramanniana AG # v1 0]	Fungi\Mucoromycota\Umbelopsidaceae
https://genome.jgi.doe.gov/mycocosm/home	
**RirFMG_pNR**	
hypothetical protein GLOIN_2v1590441 [Rhizophagus irregularis DAOM 181602=DAOM 197198]	Fungi\Mucoromycota\Glomeraceae
ACCESSION XP_025179937	
**PbrRHI_pNR**	
hypothetical protein PBRA_006660 [Plasmodiophora brassicae]	Eukaryota\Rhizaria
ACCESSION CEO98546	
**GthCRYpNR1**	
hypothetical protein GUITHDRAFT_87626 [Guillardia theta CCMP2712]	Eukaryota\Cryptophyta
ACCESSION XP_005831027	
**GthCRYpNR2**	
hypothetical protein GUITHDRAFT_77099 [Guillardia theta CCMP2712]	Eukaryota\Cryptophyta
ACCESSION XP_005825572	
**GthCRYpNR3**	
hypothetical protein GUITHDRAFT_80183 [Guillardia theta CCMP2712]	Eukaryota\Cryptophyta
ACCESSION XP_005821958	
**NgaSEucpNR**	
manganese transport protein [Nannochloropsis gaditana]	Eukaryota\Stramenopiles\Eustigmatophyceae
ACCESSION EWM26474	
**EugEgr_pNR**	
m.82023 g.82023 ORF g.82023 m.82023 comp103831_c0_seq1:1-1539+ [Euglena gracilis]	Eukaryota\Euglenozoa
https://jicbio.nbi.ac.uk/euglena/blast.html	
**SacVCC_pNR**	
TSA: Scenedesmus acutus c10390_g38_i1 transcribed RNA sequence	Viridiplantae\Chlorophyta\Chlorophyceae
ACCESSION GFUP01004201	
**KniVKl_pNR**	
Mn2+ and Fe2+ transporter [Klebsormidium nitens]	Viridiplantae\Klebsormidiophyceae

ACCESSION GAQ81386
PpaVBr_pNR
uncharacterized protein LOC112290166 [Physcomitrella patens] Viridiplantae\Bryophyta
ACCESSION XP_024391945
BarVBr_pNR
TSA: Bryum argenteum CL2102.Contig1_TBA transcribed RNA sequence Viridiplantae\Bryophyta
ACCESSION GCZP01006763
MpoVMapNR1
hypothetical protein AXG93_2779s1250 [Marchantia polymorpha subsp. ruderalis] Viridiplantae\Marchantiophyta
ACCESSION OAE27577
MpoVMapNR2
hypothetical protein AXG93_138s1000 [Marchantia polymorpha subsp. ruderalis] Viridiplantae\Marchantiophyta
ACCESSION OAE21829
pN-II:
CoxRHONpNR
TSA: Camontagnea oxyclada GWS016449_comp100953_c0_seq1 transcribed RNA sequence Rhodophyta\Nemaliophycidae
ACCESSION GFTM01047643
DraRHONpNR
TSA: Devaleraea ramentacea GWS034080_comp54033_c0_seq1 transcribed RNA sequence Rhodophyta\Nemaliophycidae
ACCESSION GFTF01054303
GefRHONpNR
TSA: Grania efflorescens SLC002_comp19390_c0_seq1 transcribed RNA sequence Rhodophyta\Nemaliophycidae
ACCESSION GFSZ01018233
CpaGLA_pNR
Glaucophyta-Cyanophora_paradoxa_ConsensusfromContig9442 spliced Eukaryota\Glaucocystophyceae
http://cyanophora.rutgers.edu/cyanophora/blast.php
DdiAMB_pNR
natural resistance-associated macrophage protein [Dictyostelium discoideum AX4] Eukaryota\Amoebozoa\Dictyostelids
ACCESSION XP_643409
AsuAMB_pNR
hypothetical protein SAMD00019534_081480 [Acytostelium subglobosum LB1] Eukaryota\Amoebozoa\Dictyostelids
ACCESSION XP_012752062
PpoAMB_pNR
TSA: Physarum polycephalum Phypoly_transcript_04218 transcribed RNA sequence Eukaryota\Amoebozoa\Myxogastria
ACCESSION GDRG01004217
aN-I:
PglVDP_eNR
overlap assembly
Picea glauca clone GQ00610_B16 mRNA sequence, ACCESSION BT100908 Viridiplantae\Pinidae
Picea glauca clone GQ03116_B04 mRNA sequence, ACCESSION BT108027
MpoVMa_eNR
hypothetical protein AXG93_531s1120 [Marchantia polymorpha subsp. ruderalis] Viridiplantae\Marchantiophyta
ACCESSION OAE22630.1
KniVKl_eNR

natural resistance-associated macrophage protein 1 [Klebsormidium nitens]   Viridiplantae\Klebsormidiophyceae
ACCESSION   GAQ80539.1
PbrRHI_eNR
hypothetical protein PBRA_005937 [Plasmodiophora brassicae]   Eukaryota\Rhizaria
ACCESSION   CEO97823
CpaGLA_eNR
Glaucophyta-Cyanophora_paradoxa_ConsensusfromContig6955 spliced   Eukaryota\Glaucocystophyceae
http://cyanophora.rutgers.edu/cyanophora/blast.php
GthCRY_eNR
hypothetical protein GUITHDRAFT_90722 [Guillardia theta CCMP2712]   Eukaryota\Cryptophyta
ACCESSION   XP_005820483
aN-II:
HveFMC_aNR
Nramp-domain-containing protein [Hesseltinella vesiculosa]   Fungi\Mucoromycota\Cunninghamellaceae
ACCESSION   ORX62099
RmiFMM_aNR
hypothetical protein RMATCC62417_04131 [Rhizopus microsporus]   Fungi\Mucoromycota\Mucorineae
ACCESSION   CEG67745
BciFMMaNR4
jgi|Bacci1|230145|estExt_Genewise1.C_2240040 [Backusella circina FSU 941 v1.0]   Fungi\Mucoromycota\Mucorineae
https://genome.jgi.doe.gov/mycocosm/home
UraFMU_aNR
jgi|Umbra1|255393|fgenesh1_kg.59_#_148_#_combest_scaffold_59_115034 [Umbelopsis ramanniana AG # v1.0]   Fungi\Mucoromycota\Umbelopsidaceae
https://genome.jgi.doe.gov/mycocosm/home
RirFMG_aNR
metal transporter nramp1-like [Rhizophagus irregularis DAOM 181602=DAOM 197198]   Fungi\Mucoromycota\Glomeraceae
ACCESSION   XP_025179950
CoxRHONaNR
TSA: Camontagnea oxyclada GWS016449_comp112235_c0_seq1 transcribed RNA sequence   Rhodophyta\Nemaliophycidae
ACCESSION   GFTM01058804
NgaSEu_aNR
2-isopropylmalate synthase? [Nannochloropsis gaditana]   Eukaryota\Stramenopiles\Eustigmatophyceae
ACCESSION   EWM28880
DdiAMB_aNR
solute carrier family 11 member 1 [Dictyostelium discoideum AX4]   Eukaryota\Amoebozoa\Dictyostelids
ACCESSION   XP_642974
AsuAMB_aNR
hypothetical protein SAMD00019534_095240 [Acytostelium subglobosum LB1]   Eukaryota\Amoebozoa\Dictyostelids
ACCESSION   XP_012750903
PpoAMB_aNR
TSA: Physarum polycephalum Phypoly_transcript_06045 transcribed RNA sequence   Eukaryota\Amoebozoa\Myxogastria
ACCESSION   GDRG01006044
EugEgr_aNR
m.72289 g.72289 ORF g.72289 m.72289 comp67242_c0_seq1:528-2183- [Euglena gracilis]   Eukaryota\Euglenozoa

https://jicbio.nbi.ac.uk/euglena/blast.html

SacVCC_aNR
TSA: Scenedesmus acutus c10326_g8_i1 transcribed RNA sequence — Viridiplantae\Chlorophyta\Chlorophyceae
ACCESSION GFUP01003099

BarVBraNRa
TSA: Bryum argenteum CL2459.Contig1_TBA transcribed RNA sequence — Viridiplantae\Bryophyta
ACCESSION GCZP01007579

PglVDP_aNR
Picea glauca clone GQ03605_L14 mRNA sequence — Viridiplantae\Pinidae
ACCESSION BT114940

MpoVMa_aNR
hypothetical protein AXG93_2294s1060 [Marchantia polymorpha subsp. ruderalis] — Viridiplantae\Marchantiophyta
ACCESSION OAE31591.1

KniVKl_aNR
Mn2+ and Fe2+ transporters of the NRAMP family [Klebsormidium nitens] — Viridiplantae\Klebsormidiophyceae
ACCESSION GAQ86250.1

PpaVBraNR1
metal transporter Nramp4-like [Physcomitrella patens] — Viridiplantae\Bryophyta
ACCESSION XP_024367603

MA (used as outgroup):

cKorach_MA
divalent metal cation transporter [Candidatus Korarchaeota archaeon] — Archaea\TACK group\Candidatus Korarchaeota
ACCESSION PMB79842

HadAHalbMA
divalent metal cation transporter [Haladaptatus sp. R4] — Archaea\Euryarchaeota\Halobacteriales
ACCESSION WP_082837784

HacAHalbMA
Mn2+ uptake NRAMP transporter MntH [Halarchaeum acidiphilum] — Archaea\Euryarchaeota\Halobacteriales
ACCESSION WP_020221592

HmoAHalbMA
divalent metal cation transporter [Halococcus morrhuae] — Archaea\Euryarchaeota\Halobacteriales
ACCESSION WP_004054047

HafAHalfMA
MULTISPECIES: divalent metal cation transporter [Haloferax] — Archaea\Euryarchaeota\Haloferacales
ACCESSION WP_008576024

HabAHalfMA
divalent metal cation transporter [Halobellus rufus] — Archaea\Euryarchaeota\Haloferacales
ACCESSION WP_081926982

SapANat_MA
divalent metal cation transporter [Saliphagus sp. LR7] — Archaea\Euryarchaeota\Natrialbales
ACCESSION WP_114579652

Appendix 7: DETAILS OF SEQUENCES USED IN FIGURE 2: SLC 11 PHYLOGENY

Outgroup:

PhaGPBout
divalent metal cation transporter [Pseudoalteromonas distincta] Bacteria; Gammaproteobacteria; Alteromonadales
ACCESSION WP_002962995

HvoEuAout
divalent metal cation transporter [Haloferax volcanii] Archaea; Euryarchaeota; Haloferacales
ACCESSION WP_004040888

BclFRCout
manganese transporter [Bacillus clausii KSM-K16] Bacteria; Terrabacteria group; Firmicutes; Bacillales
ACCESSION BAD63718

CliGPBout
divalent metal cation transporter [Congregibacter litoralis] Bacteria; Gammaproteobacteria; Cellvibrionales
ACCESSION WP_008296302

RbaPLAout
divalent metal cation transporter [Rhodopirellula baltica] Bacteria; PVC group; Planctomycetes
ACCESSION WP_011121855

HbaAPBout
divalent metal cation transporter [Hirschia baltica] Bacteria; Alphaproteobacteria; Rhodobacterales
ACCESSION WP_012778034

Mameteout
hypothetical protein GOS_2418246 [marine metagenome] unclassified sequences; ecological metagenomes
ACCESSION ECY09679

MB:

SsmSPC_MB
hypothetical protein [Sediminispirochaeta smaragdinae] Bacteria; Spirochaetes; Spirochaetia; Spirochaetales
ACCESSION WP_013255983

CteGSB_MB
hypothetical protein [Chlorobaculum tepidum] Bacteria; FCB group; Chlorobi; Chlorobiales
ACCESSION WP_010932690

DthBAC_MB
divalent metal cation transporter [Dictyoglomus thermophilum] Bacteria; Dictyoglomi; Dictyoglomia; Dictyoglomales
ACCESSION WP_012547528

LbaFRC_MB
divalent metal cation transporter [Lachnospiraceae bacterium 5_1_63FAA] Bacteria; Terrabacteria group; Firmicutes; Clostridiales
ACCESSION WP_009265836

CbeFRC_MB
divalent metal cation transporter [Clostridium beijerinckii] Bacteria; Terrabacteria group; Firmicutes; Clostridiales
ACCESSION WP_011968833

PbiCFB_MB
divalent metal cation transporter [Prevotella bivia] Bacteria; FCB group; Bacteroidetes; Bacteroidales
ACCESSION WP_004338544

DvuDPB_MB
divalent metal cation transporter [Desulfovibrio vulgaris] Bacteria; Deltaproteobacteria; Desulfovibrionales

ACCESSION WP_012611530
GloDPB_MB
divalent metal cation transporter [Geobacter lovleyi] Bacteria; Deltaproteobacteria; Desulfuromonadales;
ACCESSION WP_012469641
CaeHGC_MB
divalent metal cation transporter [Collinsella aerofaciens] Bacteria; Terrabacteria group; Actinobacteria; Coriobacteriales
ACCESSION WP_006235328
MA:
SthCHFXMA
divalent metal cation transporter [Sphaerobacter thermophilus] Bacteria; Terrabacteria group; Chloroflexi; Thermomicrobia
ACCESSION WP_012873499
HthEUA_MA
divalent metal cation transporter [Halococcus thailandensis] Archaea; Euryarchaeota; Halobacteriales
ACCESSION WP_007740131
AceFRC_MA
divalent metal cation transporter [Hungateiclostridium cellulolyticum] Bacteria; Terrabacteria group; Firmicutes; Clostridiales
ACCESSION WP_010251906
CapFRC_MA
divalent metal cation transporter [Caldanaerobacter subterraneus] Bacteria; Terrabacteria group; Firmicutes; Thermoanaerobacterales
ACCESSION WP_009610311
AciBAC_MA
Mn(2+) uptake NRAMP transporter MntH [Granulicella mallensis] Bacteria; Acidobacteria; Acidobacteriales
ACCESSION WP_014267614
SfuDPB_MA
divalent metal cation transporter [Syntrophobacter fumaroxidans] Bacteria; Deltaproteobacteria; Syntrophobacterales;
ACCESSION WP_011697512
EcoGPB_MA
Mn(2+) uptake NRAMP transporter MntH [Escherichia coli] Bacteria; Gammaproteobacteria; Enterobacterales
ACCESSION WP_000186386
CsaGPB_MA
divalent metal cation transporter [Chromohalobacter salexigens] Bacteria; Gammaproteobacteria; Oceanospirillales
ACCESSION WP_110061481
DmaBAC_MA
divalent metal cation transporter [Deinococcus maricopensis] Bacteria; Terrabacteria group; Deinococci; Deinococcales
ACCESSION WP_013557700
BpsFRC_MA
divalent metal cation transporter [Bacillus pseudomycoides] Bacteria; Terrabacteria group; Firmicutes; Bacillales
ACCESSION WP_098149025
MpaHGC_MA
divalent metal cation transporter [Mycobacterium malmoense] Bacteria; Terrabacteria group; Actinobacteria; Corynebacteriales
ACCESSION WP_007168731
BanHGC_MA
Mn(2+) uptake NRAMP transporter MntH [Bifidobacterium animalis] Bacteria; Terrabacteria group; Actinobacteria; Bifidobacteriales
ACCESSION WP_004218333

MAV:

BglBPBMAV
divalent metal cation transporter [Burkholderia gladioli] Bacteria; Betaproteobacteria; Burkholderiaceae
ACCESSION WP_013689509
SprGPBMAV
divalent metal cation transporter [Serratia sp S4] Bacteria; Gammaproteobacteria; Yersiniaceae
ACCESSION WP_017892429
BmuBPBMAV
divalent metal cation transporter [Burkholderia multivorans] Bacteria; Betaproteobacteria; Burkholderiaceae
ACCESSION WP_125902520
RvaAPBMAV
divalent metal cation transporter [Rhodomicrobium vannielii] Bacteria; Alphaproteobacteria; Rhizobiales
ACCESSION WP_013418153

MH:

OprBAC_MH
divalent metal cation transporter [Oceanithermus profundus] Bacteria; Terrabacteria group; Deinococci; Thermales
ACCESSION WP_013456683
TarGPB_MH
divalent metal cation transporter [Thiomicrorhabdus arctica] Bacteria; Gammaproteobacteria; Piscirickettsiaceae
ACCESSION WP_033394402
TbrDeT_MH
Mn(2+) uptake NRAMP transporter MntH [Thermus brockianus] Bacteria; Terrabacteria group; Deinococci; Thermales
ACCESSION WP_071678166
TfiDeT_MH
Mn(2+) uptake NRAMP transporter MntH [Thermus filiformis] Bacteria; Terrabacteria group; Deinococci; Thermales
ACCESSION WP_038063849
NitucTHAMH
Nitrosopumilales archaeon UBA219 UBA219_contig_107, whole genome shotgun sequence Archaea; TACK group; Thaumarchaeota; Nitrosopumilales
ACCESSION DAGE01000055 1|:36410-37576
Archsc407H
hypothetical protein AUJ07_06515 [Crenarchaeota archaeon 13_1_40CM_3_53_5] Archaea; TACK group; unclassified Crenarchaeota
ACCESSION OLD03308
caThorArcH
divalent metal cation transporter [Candidatus Thorarchaeota archaeon] Archaea; Asgard group; Candidatus Thorarchaeota
ACCESSION RDE15971
ThaumFn1MH
[Uncultured Crenarchaeota (1st assembly) : DRAFT_contig_70_607_len_10577_read_count_184089 49] Archaea; unclassified Archaea
manganese transport protein https://img jgi doe gov/cgi-bin/m/main cgi?section=FindGenes (IMG Gene ID: 2558670977)
CrenAsc1MH
Crenarchaeota archaeon 13_1_40CM_3_52_17 13_1_40cm_3_scaffold_139, whole genome shotgun sequence Archaea; TACK group; unclassified Crenarchaeota
ACCESSION MNGN01000055 (complement 31990 33363)
cMicrAH_MH
Candidatus Micrarchaeota archaeon CG10_big_fil_rev_8_21_14_0_10_45_29 Archaea; DPANN group; Candidatus Micrarchaeota
CG10_8_21_14_0 10_scaffold_221_c, whole genome shotgun sequence

ACCESSION PFCD01000001 (45769 47046)

FervidCreH

hypothetical protein C0179_07910 [Fervidicoccus sp] Archaea; TACK group; Crenarchaeota; Fervidicoccales

ACCESSION PNV79073

MCg:

ApiBPB_Cg

manganese transporter [Achromobacter piechaudii] Bacteria; Betaproteobacteria; Alcaligenaceae

ACCESSION WP_006220259

PflGPBCgf

putative manganese transport-related membrane protein [Pseudomonas fluorescens Pf0-1] Bacteria; Gammaproteobacteria; Pseudomonadales

ACCESSION ABA73814

CseAPB_Cg

divalent metal cation transporter [Caulobacter segnis] Bacteria; Alphaproteobacteria; Caulobacterales

ACCESSION WP_013080432

HseBPB_Cg

divalent metal cation transporter [Herbaspirillum seropedicae] Bacteria; Betaproteobacteria; Oxalobacteraceae

ACCESSION WP_013234084

CviBPB_Cg

divalent metal cation transporter [Chromobacterium violaceum] Bacteria; Betaproteobacteria; Neisseriales

ACCESSION WP_011134131

BmuBPBCgf

divalent metal cation transporter [Burkholderia multivorans] Bacteria; Betaproteobacteria; Burkholderiaceae

ACCESSION WP_012217484

VpaBPB_Cg

divalent metal cation transporter [Variovorax paradoxus] Bacteria; Betaproteobacteria; Comamonadaceae

ACCESSION WP_013544313

BgaBPBCgs

Mn(2+) uptake NRAMP transporter MntH [Burkholderia gladioli] Bacteria; Betaproteobacteria; Burkholderiaceae

ACCESSION WP_013689976

BsyBPBCgs

divalent metal cation transporter [Burkholderia sp CCGE1002] Bacteria; Betaproteobacteria; Burkholderiaceae

ACCESSION WP_013089653

MCb:

LjoFRCCbf

divalent metal cation transporter [Lactobacillus johnsonii] Bacteria; Terrabacteria group; Firmicutes; Lactobacillales

ACCESSION WP_014567503

PfrHGCCbf

divalent metal cation transporter [Propionibacterium freudenreichii] Bacteria; Terrabacteria group; Actinobacteria; Propionibacteriales

ACCESSION WP_013161931

WpaFRCCbf

divalent metal cation transporter [Weissella paramesenteroides] Bacteria; Terrabacteria group; Firmicutes; Lactobacillales

ACCESSION WP_002827536

OoeFRCCbs

manganese transporter [Oenococcus oeni ATCC BAA-1163] Bacteria; Terrabacteria group; Firmicutes; Lactobacillales

ACCESSION EAV39596

WglGPB_Cb

divalent metal cation transporter [Wigglesworthia glossinidia] Bacteria; Gammaproteobacteria; Enterobacterales

ACCESSION WP_011069922

ShaFRC_Cb

divalent metal cation transporter [Staphylococcus haemolyticus] Bacteria; Terrabacteria group; Firmicutes; Bacillales

ACCESSION WP_059748471

LcoFRCCbt

Mn(2+) uptake NRAMP transporter MntH [Lactobacillus coleohominis] Bacteria; Terrabacteria group; Firmicutes; Lactobacillales

ACCESSION WP_006917066

MCa:

BspCFB_Ca

divalent metal cation transporter [Blattabacterium sp (Blattella germanica)] Bacteria; FCB group; Bacteroidetes; Flavobacteriales

ACCESSION WP_012840866

NpuCYA_Ca

divalent metal cation transporter [Nostoc punctiforme] Bacteria; Terrabacteria group; Cyanobacteria

ACCESSION WP_012409264

PpaVER_Ca

divalent metal cation transporter [Pedosphaera parvula] Bacteria; PVC group; Verrucomicrobia

ACCESSION WP_007413308

MspAPBCaf

divalent metal cation transporter [Chelativorans sp BNC1] Bacteria; Alphaproteobacteria; Rhizobiales

ACCESSION WP_011578586

RceAPBCaf

divalent metal cation transporter [Roseomonas cervicalis] Bacteria; Alphaproteobacteria; Rhodospirillales

ACCESSION WP_007003882

ApaAPB_Ca

divalent metal cation transporter [Acetobacter pasteurianus] Bacteria; Alphaproteobacteria; Rhodospirillales

ACCESSION WP_012813165

pN-I:

Cre_GRA_pNR

manganese transport protein [Chlamydomonas reinhardtii] Eukaryota; Viridiplantae; Chlorophyta; Chlorophyceae

ACCESSION XP_001691702

MpoLWT_pNR

hypothetical protein AXG93_2779s1250 [Marchantia polymorpha subsp ruderalis] Eukaryota; Viridiplantae; Embryophyta; Marchantiophyta

ACCESSION OAE27577

KflCHA_pNR

Mn2+ and Fe2+ transporter [Klebsormidium nitens] Eukaryota; Viridiplantae; Streptophyta; Klebsormidiophyceae

ACCESSION GAQ81386

GthCRYpNR1

hypothetical protein GUITHDRAFT_87626 [Guillardia theta CCMP2712] Eukaryota; Cryptophyta; Pyrenomonadales

ACCESSION XP_005831027

RalCRY_pNR

Natural resistance-associated macrophage like domain-containing protein [Rozella allomycis CSF55] Eukaryota; Opisthokonta; Fungi; Cryptomycota

ACCESSION EPZ33297
Bde_CHY_pNR
hypothetical protein BATDEDRAFT_29076 [Batrachochytrium dendrobatidis JAM81] Eukaryota; Opisthokonta; Fungi; Chytridiomycetes
ACCESSION XP_006675619
MveFMMopNR
manganese transporter [Mortierella verticillata NRRL 6337] Eukaryota; Opisthokonta; Fungi; Mortierellomycotina
ACCESSION KFH67047
PblFMMpNR1
hypothetical protein PHYBLDRAFT_18447 [Phycomyces blakesleeanus NRRL 1555(-)] Eukaryota; Opisthokonta; Fungi; Mucoromycotina
ACCESSION XP_018296937
Cne_BAS_pNR
conserved hypothetical protein [Cryptococcus neoformans var neoformans JEC21] Eukaryota; Opisthokonta; Fungi; Basidiomycota
ACCESSION XP_572566
DhaASC_pt
DEHA2G08536p [Debaryomyces hansenii CBS767] Eukaryota; Opisthokonta; Fungi; Ascomycota
ACCESSION XP_461918
VpoASC_pf
hypothetical protein Kpol_1051p17 [Vanderwaltozyma polyspora DSM 70294] Eukaryota; Opisthokonta; Fungi; Ascomycota
ACCESSION XP_001644226
MCaU:
PspCFB_CaU
iron/manganese transporter [Pedobacter sp BAL39] Bacteria; FCB group; Bacteroidetes; Sphingobacteriales
ACCESSION WP_008241390
AaqCFB_CaU
iron/manganese transporter [Adhaeribacter aquaticus] Bacteria; FCB group; Bacteroidetes; Cytophagales
ACCESSION WP_026464449
NauCFB_CaU
iron/manganese transporter [Niabella aurantiaca] Bacteria; FCB group; Bacteroidetes; Chitinophagales
ACCESSION WP_018627415
B08CFB_CaU
NRAMP (natural resistance-associated macrophage protein) metal ion transporters
[Bacteroidetes bacterium SCGC AAA027-G08 : D416DRAFT_NODE_2_len_123085_cov_205_178_ID_17599188 81] Bacteria; FCB group; unclassified Bacteroidetes
(https://img jgi doe gov/cgi-bin/m/main cgi?section=GeneDetail&page=geneDetail&gene_oid=2619626994)
SacPTM_CaU
iron/manganese transporter [Singulisphaera acidiphila] Bacteria; PVC group; Planctomycetes
ACCESSION WP_015244408
DtiDPB_CaU
iron/manganese transporter [Desulfomonile tiedjei] Bacteria; Deltaproteobacteria; Syntrophobacterales
ACCESSION WP_014809780
BRC1BacCaU
NRAMP (natural resistance-associated macrophage protein) metal ion transporters
[candidate division BRC1 bacterium SCGC AAA252-M09 (SAK_001_73) : A252M9DRAFT_contig_4_0 5] Bacteria; unclassified Bacteria; Candidatus Sumerlaeota
(https://img jgi doe gov/cgi-bin/m/main cgi?section=GeneDetail&page=genePageMainFaa&gene_oid=2264940135)
CteFRC_CaU

Mn(2+) uptake NRAMP transporter MntH [Ruminiclostridium cellobioparum]	Bacteria; Terrabacteria group; Firmicutes; Clostridia
ACCESSION WP_004624989	
pN-II:	
**Cpa_GLA_pNR**	
Glaucophyta-Cyanophora_paradoxa_ConsensusfromContig9442 (spliced)	Eukaryota; Glaucocystophyceae; Cyanophoraceae
(http://cyanophora rutgers edu/cyanophora/)	
**CmeRHO_pNR**	
manganese transport protein [Cyanidioschyzon merolae strain 10D]	Eukaryota; Rhodophyta; Bangiophyceae
ACCESSION XP_005539209	
**PkiEXC_pNR**	
TSA: Pharyngomonas kirbyi m 30820_3prime_partial_ORF transcribed RNA sequence	Eukaryota; Heterolobosea; Pharyngomonas
Sequence ID: GECH01009913 1	
**GokEXC_pNR**	
Gefionella okellyi (Malawimonas sp. strain 249)	Eukaryota; Malawimonadidae
full-length assembly obtained by walking after translation of 48 overlapping end fragment reads (100bp)	
ACCESSION SRX3152400	
**DdiAMB_pNR**	
natural resistance-associated macrophage protein [Dictyostelium discoideum AX4]	Eukaryota; Amoebozoa; Mycetozoa; Dictyostelids
ACCESSION XP_643409	
**PpoAMB_pNR**	
TSA: Physarum polycephalum Phypoly_transcript_04218 transcribed RNA sequence	Eukaryota; Amoebozoa; Mycetozoa; Myxogastria
ACCESSION GDRG01004217	
aN-I:	
**PbrRHI_eNR**	
hypothetical protein PBRA_005937 [Plasmodiophora brassicae]	Eukaryota; Rhizaria; Cercozoa
ACCESSION CEO97823	
**Cpa_GLA_aNR**	
ConsensusfromContig6955-snap-gene-0 0	Eukaryota; Glaucocystophyceae; Cyanophoraceae
(http://cyanophora rutgers edu/cyanophora/)	
**Ota_GRA_aNR**	
Natural resistance-associated macrophage like [Ostreococcus tauri]	Eukaryota; Viridiplantae; Chlorophyta; Mamiellophyceae
ACCESSION XP_003083269	
**KflCHA_eNR**	
natural resistance-associated macrophage protein 1 [Klebsormidium nitens]	Eukaryota; Viridiplantae; Streptophyta; Klebsormidiophyceae
ACCESSION GAQ80539	
**VviEUD_aw**	
PREDICTED: metal transporter Nramp5 [Vitis vinifera]	Eukaryota; Viridiplantae; Embryophyta; eudicotyledons
ACCESSION XP_002267072	
**OsJMON_af**	
metal transporter Nramp3 [Oryza sativa Japonica Group]	Eukaryota; Viridiplantae; Embryophyta; Liliopsida
ACCESSION XP_015644306	
aN-II:	
**ChoCentaNR**	
TSA: Choanocystis sp FB-2015 c45378_g1_i1 transcribed RNA sequence	Eukaryota; Centroheliozoa; Acanthocystidae

ACCESSION   GDKX01056379
CoxRHO_aNR
TSA: Camontagnea oxyclada GWS016449_comp112235_c0_seq1 transcribed RNA sequence   Eukaryota; Rhodophyta; Florideophyceae
ACCESSION   GFTM01058804
Ddi_AMB_aNR
solute carrier family 11 member 1 [Dictyostelium discoideum AX4]   Eukaryota; Amoebozoa; Mycetozoa; Dictyostelids
ACCESSION   XP_642974
PpoAMB_aNR
TSA: Physarum polycephalum Phypoly_transcript_06045 transcribed RNA sequence   Eukaryota; Amoebozoa; Mycetozoa; Myxogastria
ACCESSION   GDRG01006044
Cco_ENT_aNR
solute carrier family 11 member 1 [Conidiobolus coronatus NRRL 28638]   Eukaryota; Opisthokonta; Fungi; Zoopagomycota
ACCESSION   KXN72436
Rir_GLO_aNR
metal transporter nramp1-like [Rhizophagus irregularis DAOM 181602=DAOM 197198]   Eukaryota; Opisthokonta; Fungi; Glomeromycotina
ACCESSION   XP_025179950
BciFMMaNR5
Backusella circina FSU 941 v1 0   Eukaryota; Opisthokonta; Fungi; Mucoromycotina
jgi|Bacci1|218203|estExt_Genewise1 C_620064 (https://genome jgi doe gov/mycocosm/home)
AthEUD_at
NRAMP metal ion transporter family protein [Arabidopsis thaliana]   Eukaryota; Viridiplantae; Embryophyta; eudicotyledons
ACCESSION   NP_193614
AthEUD_ay
metal transporter Nramp4 [Arabidopsis thaliana]   Eukaryota; Viridiplantae; Embryophyta; eudicotyledons
ACCESSION   AAF13279
KflCHA_aNR
Mn2+ and Fe2+ transporters of the NRAMP family [Klebsormidium nitens]   Eukaryota; Viridiplantae; Streptophyta; Klebsormidiophyceae
ACCESSION   GAQ86250
SmaFLA_aN
putative divalent metal transporter DMT1B [Schistosoma mansoni]   Eukaryota; Opisthokonta; Metazoa; Platyhelminthes
ACCESSION   XP_018649306
CreNEM_as
hypothetical protein FL81_02613 [Caenorhabditis remanei]   Eukaryota; Opisthokonta; Metazoa; Nematoda
ACCESSION   POM52092
DmeFLI_aN
MIP14640p, partial [Drosophila melanogaster]   Eukaryota; Opisthokonta; Metazoa; Insecta
ACCESSION   ACZ98469
CinTUN_aN
natural resistance-associated macrophage protein 2 isoform X1 [Ciona intestinalis]   Eukaryota; Opisthokonta; Metazoa; Tunicata
ACCESSION   XP_026694679
PmaBON_aN
natural resistance-associated macrophage protein [Pagrus major]   Eukaryota; Opisthokonta; Metazoa; Teleostei
ACCESSION   AAR83912
XtrFRO_a2

natural resistance-associated macrophage protein 2 [Xenopus tropicalis] Eukaryota; Opisthokonta; Metazoa; Amphibia
ACCESSION NP_001116938

AcaLIZ_a2

PREDICTED: natural resistance-associated macrophage protein 2 isoform X3 [Anolis carolinensis] Eukaryota; Opisthokonta; Metazoa; Sauropsida
ACCESSION XP_008101734

MmuROD_a2

solute carrier family 11, member 2, isoform CRA_b, partial [Mus musculus] Eukaryota; Opisthokonta; Metazoa; Mammalia
ACCESSION EDL04089 (proton-coupled divalent metal ion transporters)

XtrFRO_a1

PREDICTED: natural resistance-associated macrophage protein 1 [Xenopus tropicalis] Eukaryota; Opisthokonta; Metazoa; Amphibia
ACCESSION XP_002933999

AcaLIZ_a1

PREDICTED: natural resistance-associated macrophage protein 1 isoform X1 [Anolis carolinensis] Eukaryota; Opisthokonta; Metazoa; Sauropsida
ACCESSION XP_008108433

MmuROD_a1

natural resistance-associated macrophage protein 1 [Mus musculus] Eukaryota; Opisthokonta; Metazoa; Mammalia
ACCESSION NP_038640

Appendix 8: DETAILS OF PROTOTYPE NRAMP SEQUENCES USED IN FIGURE S3, S4, S5, S6A, S7A:

pN-I:

HveFMCpNR1
natural resistance-associated macrophage protein [Hesseltinella vesiculosa] Fungi\Mucoromycota\Cunninghamellaceae
ACCESSION ORX48876

HveFMCpNR2
natural resistance-associated macrophage protein [Hesseltinella vesiculosa] Fungi\Mucoromycota\Cunninghamellaceae
ACCESSION ORX57495

BciMUCpNR1
jgi|Bacci1|237824|estExt_Genewise1Plus C_60434 [Backusella circina FSU 941 v1 0] Fungi\Mucoromycota\Mucorineae
https://genome.jgi.doe.gov/mycocosm/home

RmiFMM_pNR
natural resistance-associated macrophage protein [Rhizopus microsporus ATCC 52813] Fungi\Mucoromycota\Mucorineae
ACCESSION XP_023468363

UraMUC_pNR
jgi|Umbra1|230632|fgenesh1_kg 13_#_154_#_combest_scaffold_13_17261 [Umbelopsis ramanniana AG # v1 0] Fungi\Mucoromycota\Umbelopsidaceae
https://genome.jgi.doe.gov/mycocosm/home

RirFMG_pNR
hypothetical protein GLOIN_2v1590441 [Rhizophagus irregularis DAOM 181602=DAOM 197198] Fungi\Mucoromycota\Glomeraceae
ACCESSION XP_025179937

PbrRHI_pNR
hypothetical protein PBRA_006660 [Plasmodiophora brassicae] Eukaryota\Rhizaria
ACCESSION CEO98546

GthCRYpNR1
hypothetical protein GUITHDRAFT_87626 [Guillardia theta CCMP2712] Eukaryota\Cryptophyta
ACCESSION XP_005831027

GthCRYpNR2
hypothetical protein GUITHDRAFT_77099 [Guillardia theta CCMP2712] Eukaryota\Cryptophyta
ACCESSION XP_005825572

GthCRYpNR3
hypothetical protein GUITHDRAFT_80183 [Guillardia theta CCMP2712] Eukaryota\Cryptophyta
ACCESSION XP_005821958

NgaSEucpNR
manganese transport protein [Nannochloropsis gaditana] Eukaryota\Stramenopiles\Eustigmatophyceae
ACCESSION EWM26474

SacVCC_pNR
TSA: Scenedesmus acutus c10390_g38_i1 transcribed RNA sequence Viridiplantae\Chlorophyta\Chlorophyceae
ACCESSION GFUP01004201

SspVCC_pNR
mntH [Scenedesmus sp. PABB004] Viridiplantae\Chlorophyta\Chlorophyceae
ACCESSION KAF8069422.1

EdeVCC_pNR
hypothetical protein HYH03_014457 [Edaphochlamys debaryana] Viridiplantae\Chlorophyta\Chlorophyceae
ACCESSION KAG2486960.1

EugEgr_pNR
m.82023 g.82023 ORF g.82023 m.82023 comp103831_c0_seq1:1-1539+ [Euglena gracilis]   Eukaryota\Euglenozoa
https://jicbio.nbi.ac.uk/euglena/blast.html
TspVCT_pNR
MAG: manganese transporter [Trebouxia sp. A1-2]   Viridiplantae\Chlorophyta\Trebouxiophyceae
ACCESSION   KAA6427692.1
KniVKl_pNR
Mn2+ and Fe2+ transporter [Klebsormidium nitens]   Viridiplantae\Klebsormidiophyceae
ACCESSION   GAQ81386
BarVBr_pNR
TSA: Bryum argenteum CL2102.Contig1_TBA transcribed RNA sequence   Viridiplantae\Bryophyta
ACCESSION   GCZP01006763
MpoVMapNR2
hypothetical protein AXG93_138s1000 [Marchantia polymorpha subsp. ruderalis]   Viridiplantae\Marchantiophyta
ACCESSION   OAE21829

pN-II:
Cpa_GLA_pNR
Glaucophyta-Cyanophora_paradoxa_ConsensusfromContig9442 (spliced)   Eukaryota; Glaucocystophyceae; Cyanophoraceae
(http://cyanophora rutgers edu/cyanophora/)
Ddi_AMB_pNR
natural resistance-associated macrophage protein [Dictyostelium discoideum AX4]   Eukaryota; Amoebozoa; Mycetozoa; Dictyostelids
ACCESSION   XP_643409
PpoAMB_pNR
TSA: Physarum polycephalum Phypoly_transcript_04218 transcribed RNA sequence   Eukaryota; Amoebozoa; Mycetozoa; Myxogastria
ACCESSION   GDRG01004217
GliRHOFpNR
TSA: Grateloupia livida scaffold-IKIZ-2013992-Grateloupia_livida transcribed RNA sequence   Eukaryota; Rhodophyta; Florideophyceae
ACCESSION   GFKR01005500
CmeRHO_pNR
manganese transport protein [Cyanidioschyzon merolae strain 10D]   Eukaryota; Rhodophyta; Bangiophyceae
ACCESSION   XP_005539209
GsuRHO_pNR
metal ion (Mn2+-iron) transporter, Nramp family   Eukaryota; Rhodophyta; Bangiophyceae
ACCESSION
NispSTBpNR
TSA: Nitzschia sp. ChengR-2013 comp39201_c0_seq1 transcribed RNA sequence   Eukaryota; Stramenopiles; Bacillariophyta
ACCESSION
SvuSPXcpNR
TSA: Sargassum vulgare Sample_MAC_c78784.graph_c0_seq1 transcribed RNA sequence   Eukaryota; Stramenopiles; PX clade
ACCESSION
CglGLA_pNR
Cyanoptyche gloeocystis   Eukaryota; Glaucocystophyceae; Cyanoptyche
full-length assembly obtained by walking after translation of 61 overlapping end fragment reads (100bp)

ACCESSION SRX554018

GnoGLA_pNR

Glaucocystis cf. nostochinearum — Eukaryota; Glaucocystophyceae; Glaucocystaceae

full-length assembly obtained by walking after translation of 98 overlapping end fragment reads (100bp)

ACCESSION ERX3508887

GwiGLA_pNR

Gloeochaete wittrockiana — Eukaryota; Glaucocystophyceae; Gloeochaetaceae

full-length assembly obtained by walking after translation of 101 overlapping end fragment reads (100bp)

ACCESSION ERX2100179

PkiEXC_pNR

TSA: Pharyngomonas kirbyi m 30820_3prime_partial_ORF transcribed RNA sequence — Eukaryota; Heterolobosea; Pharyngomonas

Sequence ID: GECH01009913 1

PcoEXCpNRa

Percolomonas cosmopolitus AE-1 — Eukaryota; Heterolobosea; Percolomonadidae

full-length assembly obtained by walking after translation of 313 overlapping end fragment reads (100bp)

ACCESSION SRX551163

PcoEXCpNRb

Percolomonas cosmopolitus AE-1 — Eukaryota; Heterolobosea; Percolomonadidae

full-length assembly obtained by walking after translation of 286 overlapping end fragment reads (100bp)

ACCESSION SRX551163

AncyANCpNR

Ancyromonas sigmoides CCAP 1958/3 — Eukaryota; Ancyromonadidae

full-length assembly obtained by walking after translation of 61 overlapping end fragment reads (100bp)

ACCESSION SRX3153021

GokEXC_pNR

Gefionella okellyi (Malawimonas sp. strain 249) — Eukaryota; Malawimonadidae

full-length assembly obtained by walking after translation of 48 overlapping end fragment reads (100bp)

ACCESSION SRX3152400
