# Supplementary material for: Nramp: Deprive and conquer?
Source: Front Cell Dev Biol. 2022 Oct 13;10:988866. doi: 10.3389/fcell.2022.988866 (PMC9606685; doi:10.3389/fcell.2022.988866)
Supplement: Supplementary file 2 [file DataSheet1.PDF]

Transferred gene	Eukaryotic donor	Prokaryotic recipient	HGT event*	Reference
"$\alpha\beta$-Tubulin"	primitive eukaryote	*Prosthecobacter*	ancient	Martin-Galiano, Oliva, et al., 2011<br>PMID: 21467045
glycoside-hydrolase	archaeplastida	Bacteroidetes<br>γ-Proteobacterium	ancient*	Arias, Danchin et al., 2012<br>PMID: 22934241
bisphosphate aldolase	red algae	Cyanobacteria	recent*	Rogers, Patron & Keeling, 2007<br>PMID: 17584924
GALA LRR	plant lineage	*Ralstonia solanacearum*	ancient	Kajava, Anisimova & Peeters, 2008<br>PMID: 18301771
PS-LRR	plants	Proteobacteria, Cyanobacteria<br>Bacteroidetes	recent*	Miyashita, Kuroki et al., 2013<br>doi: 10.4236/ns.2013.55074
pathogenesis related 1	plants (e.g., soybean)	*Streptomyces* spp.	recent	Armijos-Jaramillo, Santander-Gordon et al., 2017<br>PMID: 27530704
GAPDH	opisthokont	*Clostridium* spp.	ancient	Takishita & Inagaki, 2008<br>PMID: 18420358
44 different genes	protozoan host	*Legionella* spp.	ancient	de Felipe, Pampou et al., 2005<br>PMID: 16267296
chitin synthase (division 1)	mycota	α-,γ-Proteobacteria	recent*	Gonçalves, Brouillet et al., 2016<br>PMID: 27881071
Actin and profilin	marine invertebrate	*Microcystis aeruginosa*	recent*	Guljamow, Jenke-Kodama et al., 2007<br>PMID: 17803924
zincin-like protease	insects	Proteobacteria<br>Chloroflexi, Euryarchaeota	recent*	Lenart, Dudkiewicz et al., 2013<br>PMID: 23671590
α-amylase	insects	γ-Proteobacteria,<br>Actinobacteria, Firmicute	recent*	Da Lage, Feller & Janecek, 2004<br>PMID: 14704857
Karilysin	insects	*Tannerella forsythia*	recent	Cerda-Costa, Guevara et al., 2011<br>PMID: 21166898
Ubiquitin (Uba52)	*Homo sapiens*	*Bacteroides* spp.	recent*	Patrick & Blakely, 2012<br>PMID: 23061022
LINE L1 (fragment)	*Homo sapiens*	*Neisseria gonorrhoeae*	recent	Anderson & Seifert, 2011<br>PMID: 21325040
Qa-, Qb-, Qc- & R-SNARES	eukaryotic host cell	*Legionella* spp.<br>GPB bacteria<br>*Berbellia cookevillensis*<br>*Fluoribacter gormanii*	ancient	Neveu, Khalifeh et al., 2020<br>PMID: 32442459

Table S1. *Examples of HGT from eukaryote to prokaryote*

*possible secondary inter-bacteria HGTs

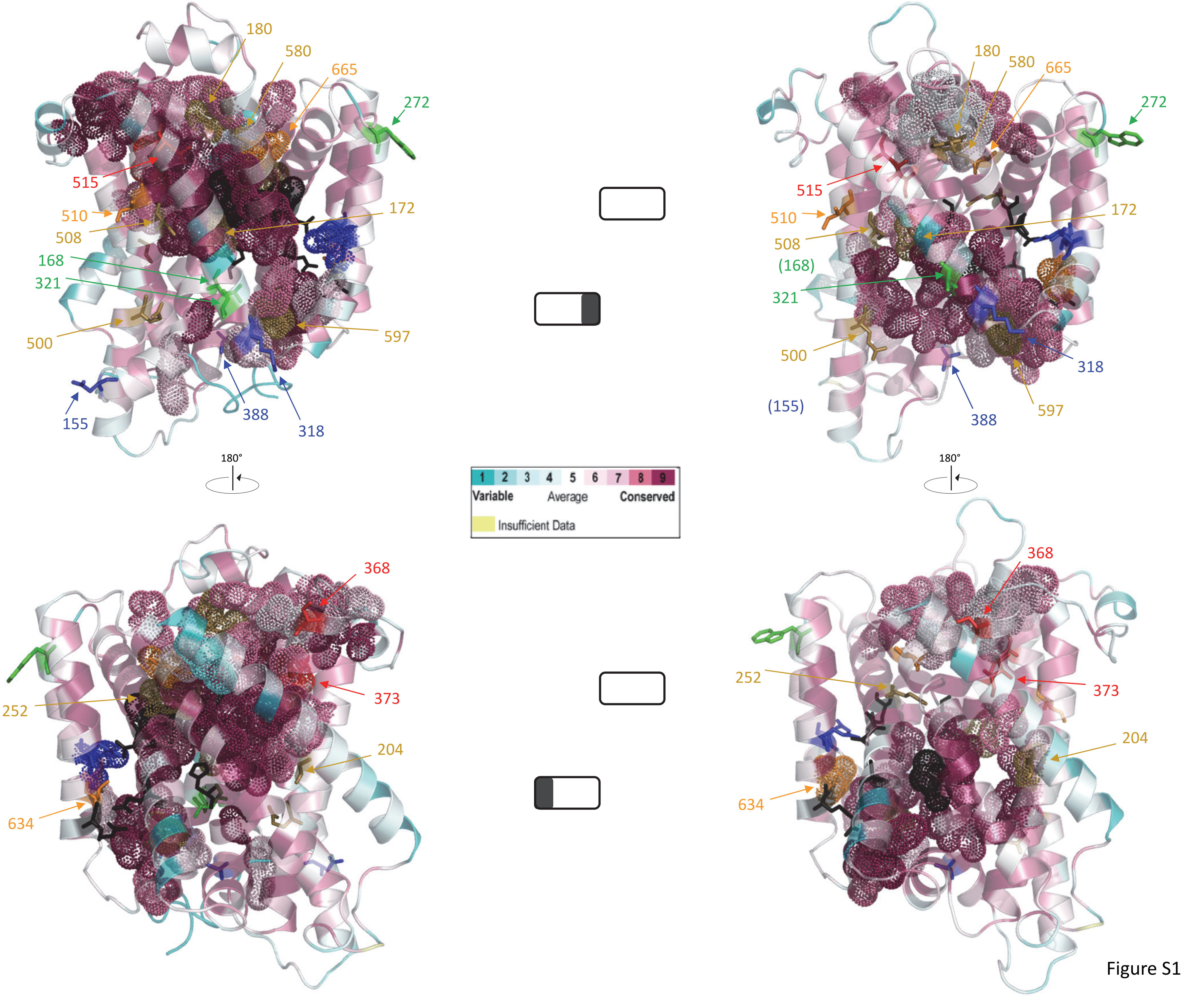
180
580
665
272
515
510
508
172
168
321
500
597
155
388
318
(168)
(155)
180°
1 2 3 4 5 6 7 8 9
Variable
Average
Conserved
Insufficient Data
368
252
373
204
634

Figure S1

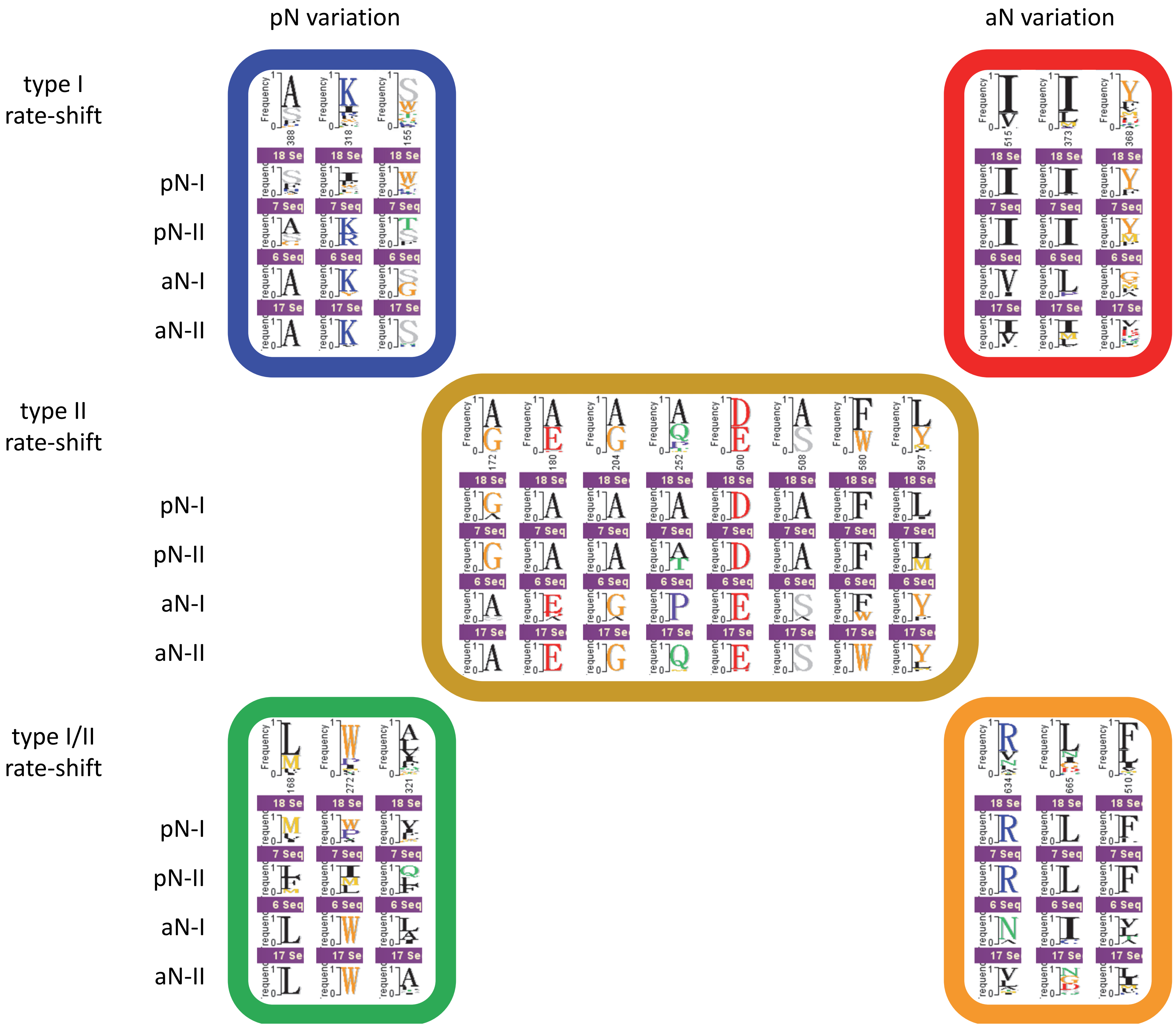

Figure S1 cntn'd

LOGO#1 (TMS1)		1	2	3	4	5	6	7	8	9	10	11	12	13	14	15	16	17	18	19
OutG	(4)	[GKY]	[PSA]	[GAS]	[ATPVLIFMYSW]	[LIMVFWY]	[VILT]	[ATSVM]	A	[ASG]	[FI]	[IVT]	G	P	G	[TS]	[VIL]	[TIVASM]	[TVALISMK]	[CASY]
MB	(10)	G	[PL]	G	[LIFM]	[LIFV]	[VI]	[TA]	[VILM]	G	F	[IV]	D	P	G	N	W	[AVG]	[STA]	N
MAV	(7)	G	P	A	[VIML]	[IVMT]	[AVSIT]	[SA]	[IV]	[AG]	Y	[MVIT]	D	P	G	[ND]	[FYIL]	[AV]	[TA]	[NG]
MA	(3)	[GA]	[PAS]	[AGS]	[FLVMIC]	[VILA]	[AVITS]	[ASGC]	[VILAMS]	[AG]	[YF]	[VILMA]	D	[PS]	G	N	[VFYWIL]	[AGSVIT]	[TASV]	[NSADG]
MH	(6)	G	[PLST]	[AGST]	[LFTVMI]	[ILVM]	[VIT]	[SAG]	[VIML]	[AG]	Y	[MIVFL]	D	P	G	N	[WFY]	[GA]	[TSA]	[DNS]
MCa	(6)	[GA]	[PR]	[GA]	[YLFASWMV]	[LMIV]	[VI]	[ASG]	[VTI]	G	[YF]	[MVIL]	D	P	G	N	W	[AGSE]	[TS]	[DSAGN]
MCb	(9)	G	P	[GA]	[ALIVSMTEF]	L	[VI]	[AS]	V	G	Y	[MI]	[DG]	[PS]	G	N	W	[ISVLA]	[TA]	[SA]
MCg	(10)	G	[PA]	G	[LAVSFM]	[LM]	[VI]	[SA]	[VIA]	[GC]	Y	[MI]	D	P	G	N	W	A	T	[DASG]
MCaU	(12)	G	P	A	[YFLV]	[LMI]	[VI]	[SAGC]	[VI]	G	Y	[ML]	D	P	G	N	W	A	T	[DG]

LOGO#4 (TMS6)		1	2	3	4	5	6	7	8	9	10	11	12	13	14	15
OutG	(3)	[LIV]	[IVLM]	G	T	[TQS]	[VIT]	[VP]	[PGT]	Y	[NSTA]	[LFIV]	[FY]	[LIM]	[HYQ]	[ASTV]
MB	(9)	[VIMAL]	[LMI]	G	A	[VL]	[VIL]	M	P	H	N	[LIMV]	[FY]	L	H	S
MAV	(8)	[IVML]	[IVLF]	G	A	T	[VIL]	M	P	H	[ATVS]	[ILV]	[YFL]	L	H	[SA]
MA	(6)	[IMVL]	[LIVMFT]	G	A	T	[VILA]	M	[PS]	H	[AVSNT]	[IVL]	[YFHW]	[LAVMI]	H	[SGP]
MH	(7)	[IVM]	[ILVM]	G	A	T	[VI]	M	P	H	[ANVT]	[LVIF]	[FYILVMAW]	[LV]	H	[SGTP]
MCa	(9)	[IMT]	[LIVM]	G	A	T	[VI]	M	P	H	N	[LI]	[YF]	L	H	[STA]
MCb	(7)	I	[IVML]	G	A	T	[VIM]	[MT]	[PL]	H	[ND]	[LIFV]	[YF]	L	[HG]	S
MCg	(10)	[IVM]	[LVIF]	G	[AV]	T	[VI]	M	P	H	N	L	Y	L	H	[STA]
MCaU	(11)	[IM]	[IL]	G	A	T	[VI]	M	P	H	N	[LIM]	Y	L	H	S

LOGO#6 (TMS8)		1	2	3	4	5	6	7	8	9
OutG	(2)	[AGST]	G	[ILFVMT]	[TS]	S	[ASTVCGM]	[IMLTVAS]	[TAS]	[ASGT]
MB	(1)	[AS]	G	[ILVFA]	[SAG]	[SA]	[TSCA]	[IVMTALSF]	[TS]	[ASC]
MAV	(4)	[SA]	G	[IVLFM]	S	[SA]	S	[VATIMS]	V	[GA]
MA	(0)	[SA]	[GSA]	[LFIVAH]	[ASGVT]	[SAG]	[TSA]	[SVATGILC]	[VITAGL]	[GAS]
MH	(0)	[SA]	[GS]	[ILVT]	[SAT]	[SA]	[ST]	[TAVILM]	[TVLASM]	[GSA]
MCa	(2)	[SCA]	G	[QLITVMHF]	[NSCA]	[SA]	[TSAV]	[VLIF]	T	[GAT]
MCb	(1)	[STA]	[GS]	[QLMH]	[NSI]	[SAF]	[TS]	I	[TA]	[GSA]
MCg	(4)	[SCA]	G	[QLIM]	[SN]	[SA]	T	[FVL]	T	G
MCaU	(5)	[AS]	G	Q	[SA]	S	T	[VIL]	[TS]	G

LOGO#2 (TMS3)		1	2	3	4	5	6	7	8	9	10
OutG	(2)	[ASTIGCV]	[ASVL]	[YFT]	[EQIMA]	[AGTSM]	G	[ND]	[ILTMV]	[STLAGVIMNR]	G
MB	(4)	[IVALQ]	[SALGFM]	T	[SADTGVMQY]	[LMVFITA]	A	E	[ILVFY]	[LIVM]	G
MAV	(4)	[MI]	A	T	[DEG]	[LI]	A	[EAQ]	[FLTS]	[LVMI]	G
MA	(3)	[MIAVGL]	[AMSFGITV]	[TC]	[DNEGY]	[LVIMAF]	A	E	[VFILM]	[IVLMAT]	G
MH	(4)	[ALVIM]	[ASMT]	[TV]	D	[LMI]	A	E	[FYILV]	[LMVIT]	G
MCa	(3)	[IACVTSMFL]	[AS]	[CTMA]	[DN]	[LIVF]	A	E	[VLI]	[ILV]	G
MCb	(2)	[MIVC]	[ACS]	T	[DE]	[ILVMFT]	[AT]	[EGQS]	[VI]	[IVLG]	G
MCg	(4)	[IVL]	[AS]	[CT]	D	[LVIM]	[AS]	E	V	[LVI]	G
MCaU	(4)	[AIVTG]	[AS]	[CT]	D	[LIM]	A	E	[VILA]	[LIV]	G

LOGO#3 (TMS4)		1	2	3	4	5
OutG	(0)	[ILVFACSTGMP]	[ASIVCGTL]	[FAGLISVWMCTY]	[LAIVGFSTCMWPRY]	[LIVAFMT]
MB	(0)	[FVLAIGTMS]	[VICSTAEKD]	[LIVFSMTAGC]	[IWFVLYAGTCS]	[MLCFIVAT]
MAV	(0)	[VIALFGT]	[TSAV]	[YCFWIGLMNSTV]	[AGLVFCIT]	[ILVFM]
MA	(0)	[VAIGLFTCSM]	[STAVGM]	[FMLIYTVWASCG]	[ALVGIFMTWSC]	[LIVMAST]
MH	(1)	D	[VIT]	[LIFVA]	[LIFVA]	[ILF]
MCa	(0)	[DE]	[VATILS]	[FLIMVA]	[LVIAMFG]	[IVLMA]
MCb	(0)	[DN]	[VI]	[LFIVM]	[LIVMG]	[LVI]
MCg	(0)	[DGACSV]	[TMSV]	[LFVMI]	[ILAVG]	[VIML]
MCaU	(1)	D	[TSVA]	[FLVIM]	[LIV]	[LIFMV]

LOGO#5 (TMS7)		1	2	3	4	5	6
OutG	(0)	[GAI]	[LIVFMATG]	[IVMT]	[STAM]	[MILATVS]	[ASCLTGFVIM]
MB	(1)	[WFL]	[AGLVCIMT]	[IV]	N	[SAC]	[ASG]
MAV	(1)	[GA]	[LMFAVCIGT]	[VI]	N	[MILVA]	[AG]
MA	(0)	[GFWASMTL]	[ALTFSVGIMC]	[VILAMG]	[NGT]	[LIAMVSGTCEW]	[ASGCVTLIN]
MH	(1)	[WGSA]	[LMVAFITCG]	[VIMAL]	N	[ASVGT]	[ASGIT]
MCa	(1)	[LFMITC]	[FLTVICGMA]	[IVL]	N	[ASGCTV]	[ASGML]
MCb	(1)	[FLMCTW]	[VIFLM]	[VI]	N	[SACTVM]	[LMFI]
MCg	(0)	[LFMTAV]	[LVFTMCIAGP]	[VIL]	[NQ]	[AGMVCS]	[ASGT]
MCaU	(1)	[FLM]	[FLVI]	[VI]	N	[ASTV]	[AGSFV]

Figure S2. Individual logos (#1-6) were combined into patterns that were used in PHI-Blast analyses.

			OutG	MntH B	MntH A	MntH AV	MntH H	MntH Cg	MntH Cb	MntH Ca	MntH CaU
Archaea			13.0	0.0	0.4	0.0	65.6	0.0	0.0	0.0	0.0
	Euryarchaeota		13.0								
	Halobacteria										
		Natrialbales	5.7								
		Halobacteriales	4.0		0.1						
		Haloferacales	3.3		0.3						
	TACK						54.7				
		Crenarchaeota					18.8				
		Thaumarchaeota					28.1				
Bacteria			87.0	100.0	99.6	99.9	34.4	99.9	100.0	99.9	99.8
	Proteobacteria		31.8	4.9	75.3	99.0	18.8	99.2	0.6	37.0	2.3
		AlphaPB	5.6			32.3	6.3	3.7		27.0	
		BetaPB				49.0		40.0		2.4	
		GammaPB	26.2		75.1	17.8	12.5	55.4	0.5	6.9	
		DeltaPB		4.8	0.2					0.5	2.3
	FCB group		33.6	55.5	0.1					20.8	89.4
		Bacteroidetes	32.1	53.0	0.1					19.4	83.1
		Ignavibacteriae								0.2	1.9
		Gemmatimonadetes								1.3	
	Terrabacteria		18.0	35.5	23.8	0.5	15.6		99.4	35.4	2.1
		Chloroflexi			0.6					0.3	0.6
		Actinobacteria	6.1	2.3	9.5	0.3			0.8		1.5
		Firmicutes	11.7	32.9	10.9				98.6	27.6	
		Cyanobacteria								7.2	
		Deinococcus-Thermus			0.5		15.6				
		Armatimonadetes			0.1					0.4	
	PVC group		1.4	0.2	0.2					2.3	1.3
		Planctomycetes	1.4								1.3
		Verrucomicrobia			0.2					1.9	
		Chlamydiae			0.02					0.1	
		Lentisphaerae		0.2							
	Acidobacteria				0.2					1.9	2.5
		Acidobacteriales			0.2					0.7	
		Solibacterales									1.0
	Balneolaeota		0.4								
	Fusobacteria		1.4								0.4
	Spirochaetes			0.6	0.2						
	Caldiserica			0.2							
	Dictyoglomi			0.6							
	Deferribacteres			0.2							
	Elusimicrobia			1.1							
	Nitrospirae			0.2							
	Patescibacteria									1.6	

Table S2. Summary of MntH group-specific taxonomic distribution (%) produced by PHI-Blast analyses.

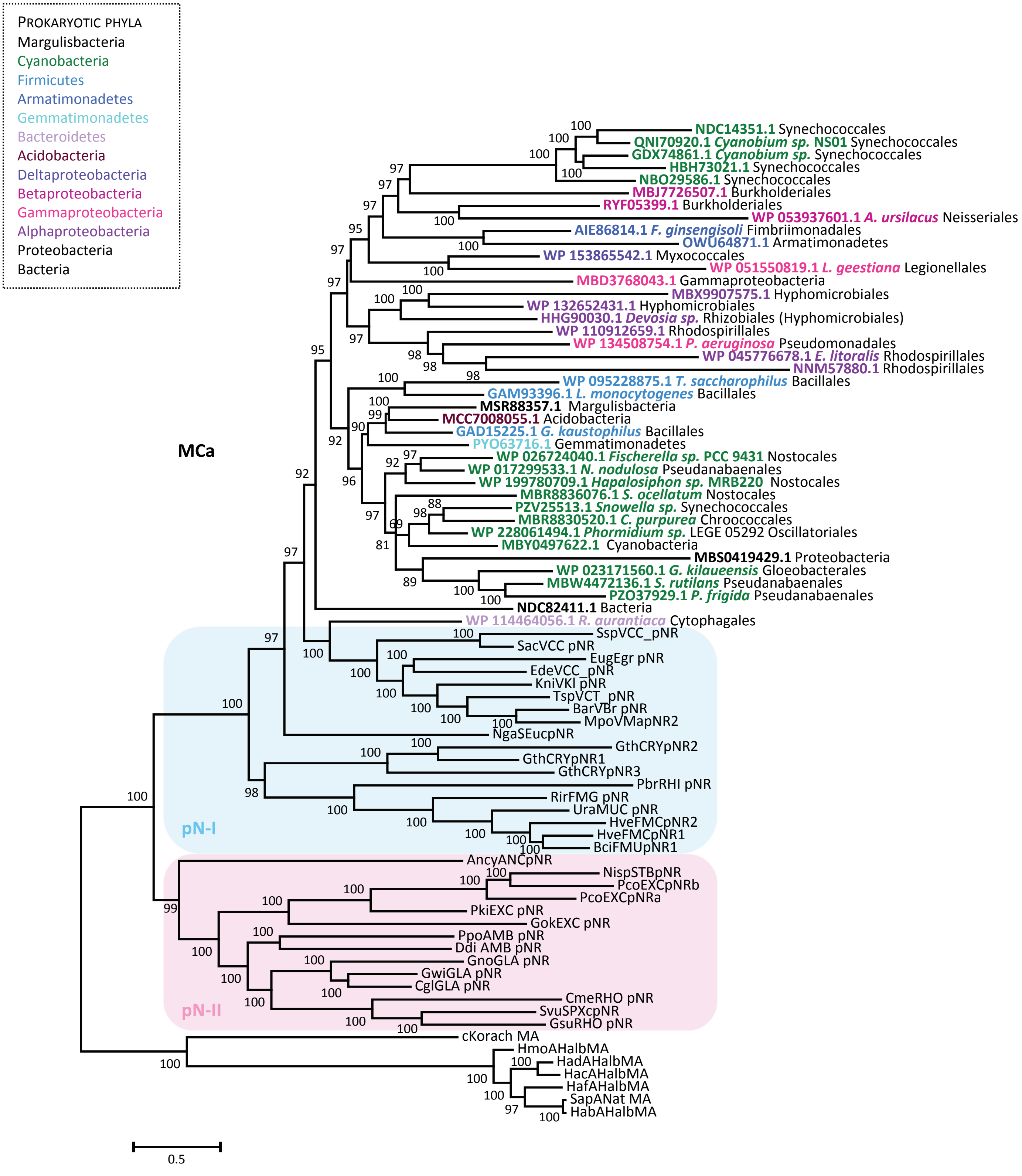
PROKARYOTIC PHYLA
Margulisbacteria
Cyanobacteria
Firmicutes
Armatimonadetes
Gemmatimonadetes
Bacteroidetes
Acidobacteria
Deltaproteobacteria
Betaproteobacteria
Gammaproteobacteria
Alphaproteobacteria
Proteobacteria
Bacteria
MCa
pN-I
pN-II
NDC14351.1 Synechococcales
QNI70920.1 Cyanobium sp. NS01 Synechococcales
GDX74861.1 Cyanobium sp. Synechococcales
HBH73021.1 Synechococcales
NBO29586.1 Synechococcales
MBJ7726507.1 Burkholderiales
RYF05399.1 Burkholderiales
WP 053937601.1 A. ursilacus Neisseriales
AIE86814.1 F. ginsengisoli Fimbriimonadales
OWU64871.1 Armatimonadetes
WP 153865542.1 Myxococcales
WP 051550819.1 L. geestiana Legionellales
MBD3768043.1 Gammaproteobacteria
MBX9907575.1 Hyphomicrobiales
WP 132652431.1 Hyphomicrobiales
HHG90030.1 Devosia sp. Rhizobiales (Hyphomicrobiales)
WP 110912659.1 Rhodospirillales
WP 134508754.1 P. aeruginosa Pseudomonadales
WP 045776678.1 E. litoralis Rhodospirillales
NNM57880.1 Rhodospirillales
WP 095228875.1 T. saccharophilus Bacillales
GAM93396.1 L. monocytogenes Bacillales
MSR88357.1 Margulisbacteria
MCC7008055.1 Acidobacteria
GAD15225.1 G. kaustophilus Bacillales
PYO63716.1 Gemmatimonadetes
WP 026724040.1 Fischerella sp. PCC 9431 Nostocales
WP 017299533.1 N. nodulosa Pseudanabaenales
WP 199780709.1 Hapalosiphon sp. MRB220 Nostocales
MBR8836076.1 S. ocellatum Nostocales
PZV25513.1 Snowella sp. Synechococcales
MBR8830520.1 C. purpurea Chroococcales
WP 228061494.1 Phormidium sp. LEGE 05292 Oscillatoriales
MBY0497622.1 Cyanobacteria
MBS0419429.1 Proteobacteria
WP 023171560.1 G. kilaueensis Gloeobacterales
MBW4472136.1 S. rutilans Pseudanabaenales
PZO37929.1 P. frigida Pseudanabaenales
NDC82411.1 Bacteria
WP 114464056.1 R. aurantiaca Cytophagales
SspVCC_pNR
SacVCC pNR
EugEgr pNR
EdeVCC_pNR
KniVKl pNR
TspVCT_pNR
BarVBr pNR
MpoVMapNR2
NgaSEucpNR
GthCRYpNR2
GthCRYpNR1
GthCRYpNR3
PbrRHI pNR
RirFMG pNR
UraMUC pNR
HveFMCpNR2
HveFMCpNR1
BciFMUpNR1
AncyANCpNR
NispSTBpNR
PcoEXCpNRb
PcoEXCpNRa
PkiEXC pNR
GokEXC pNR
PpoAMB pNR
Ddi AMB pNR
GnoGLA pNR
GwiGLA pNR
CglGLA pNR
CmeRHO pNR
SvuSPXcpNR
GsuRHO pNR
cKorach MA
HmoAHalbMA
HadAHalbMA
HacAHalbMA
HafAHalbMA
SapANat MA
HabAHalbMA
0.5

Figure S3

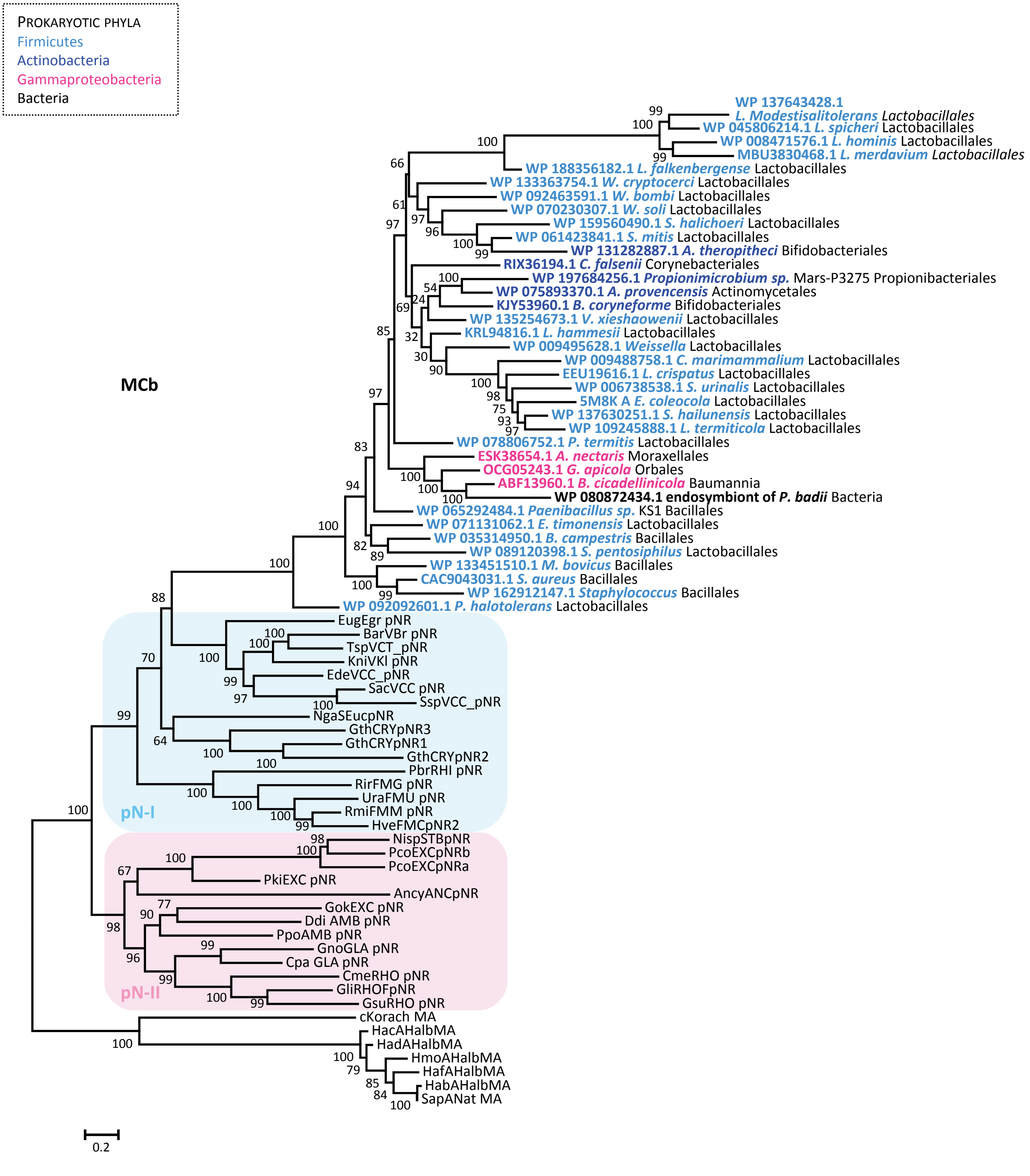
PROKARYOTIC PHYLA
Firmicutes
Actinobacteria
Gammaproteobacteria
Bacteria
MCb
WP 137643428.1 L. Modestisalitolerans Lactobacillales
WP 045806214.1 L. spicheri Lactobacillales
WP 008471576.1 L. hominis Lactobacillales
MBU3830468.1 L. merdavium Lactobacillales
WP 188356182.1 L. falkenbergense Lactobacillales
WP 133363754.1 W. cryptocerci Lactobacillales
WP 092463591.1 W. bombi Lactobacillales
WP 070230307.1 W. soli Lactobacillales
WP 159560490.1 S. halichoeri Lactobacillales
WP 061423841.1 S. mitis Lactobacillales
WP 131282887.1 A. theropitheci Bifidobacteriales
RIX36194.1 C. falsenii Corynebacteriales
WP 197684256.1 Propionimicrobium sp. Mars-P3275 Propionibacteriales
WP 075893370.1 A. provencensis Actinomycetales
KJY53960.1 B. coryneforme Bifidobacteriales
WP 135254673.1 V. xieshaowenii Lactobacillales
KRL94816.1 L. hammesii Lactobacillales
WP 009495628.1 Weissella Lactobacillales
WP 009488758.1 C. marimammalium Lactobacillales
EEU19616.1 L. crispatus Lactobacillales
WP 006738538.1 S. urinalis Lactobacillales
5M8K A E. coleocola Lactobacillales
WP 137630251.1 S. hailunensis Lactobacillales
WP 109245888.1 L. termiticola Lactobacillales
WP 078806752.1 P. termitis Lactobacillales
ESK38654.1 A. nectaris Moraxellales
OCG05243.1 G. apicola Orbales
ABF13960.1 B. cicadellinicola Baumannia
WP 080872434.1 endosymbiont of P. badii Bacteria
WP 065292484.1 Paenibacillus sp. KS1 Bacillales
WP 071131062.1 E. timonensis Lactobacillales
WP 035314950.1 B. campestris Bacillales
WP 089120398.1 S. pentosiphilus Lactobacillales
WP 133451510.1 M. bovicus Bacillales
CAC9043031.1 S. aureus Bacillales
WP 162912147.1 Staphylococcus Bacillales
WP 092092601.1 P. halotolerans Lactobacillales
EugEgr pNR
BarVBr pNR
TspVCT_pNR
KniVKI pNR
EdeVCC_pNR
SacVCC pNR
SspVCC_pNR
NgaSEucpNR
GthCRYpNR3
GthCRYpNR1
GthCRYpNR2
PbrRHI pNR
RirFMG pNR
UraFMU pNR
RmiFMM pNR
HveFMCpNR2
pN-I
NispSTBpNR
PcoEXCpNRb
PcoEXCpNRa
PkiEXC pNR
AncyANCpNR
GokEXC pNR
Ddi AMB pNR
PpoAMB pNR
GnoGLA pNR
Cpa GLA pNR
CmeRHO pNR
GliRHOFpNR
GsuRHO pNR
pN-II
cKorach MA
HacAHalbMA
HadAHalbMA
HmoAHalbMA
HafAHalbMA
HabAHalbMA
SapANat MA
0.2

Figure S4

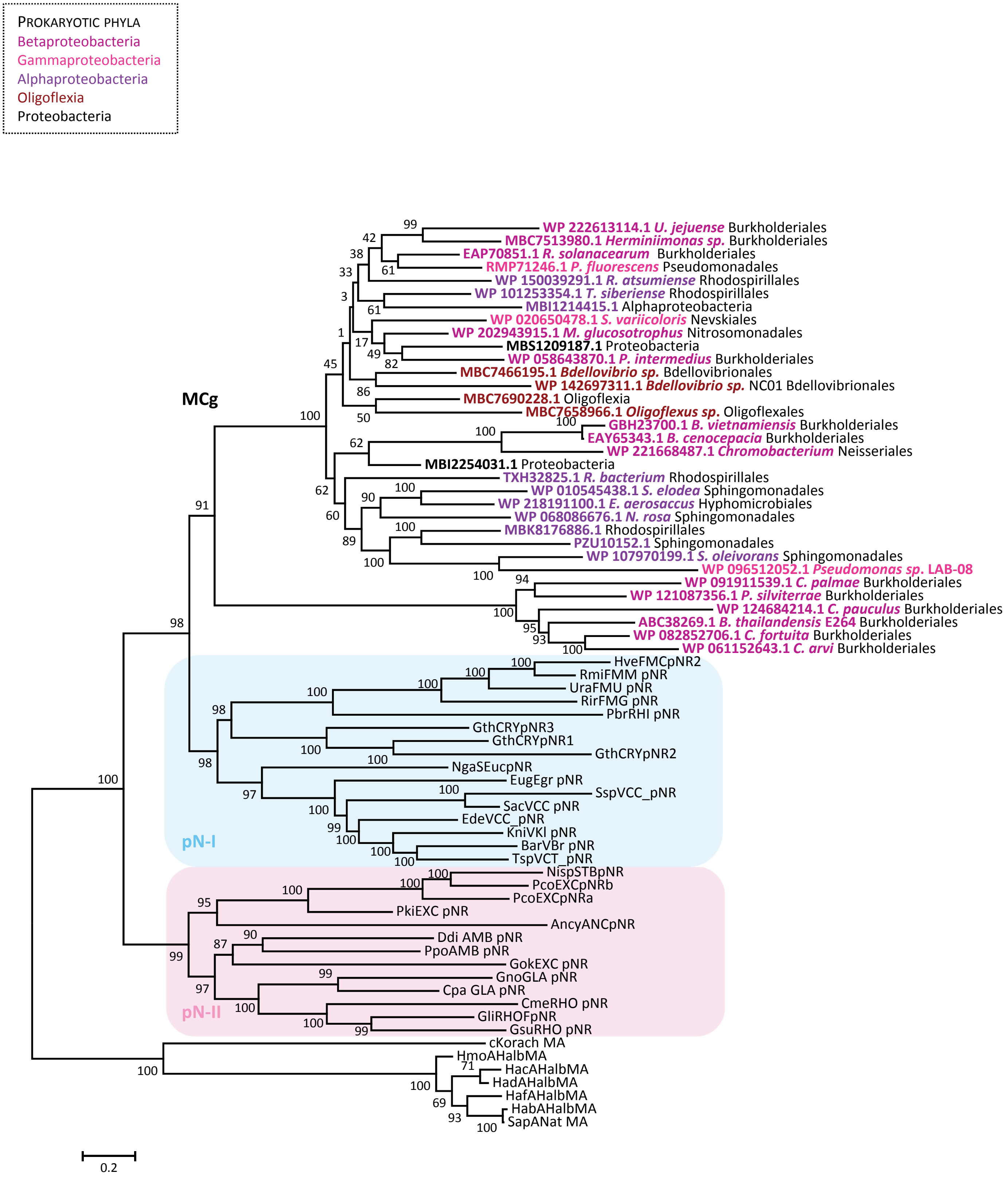
PROKARYOTIC PHYLA
Betaproteobacteria
Gammaproteobacteria
Alphaproteobacteria
Oligoflexia
Proteobacteria
MCg
pN-I
pN-II
WP 222613114.1 U. jejuense Burkholderiales
MBC7513980.1 Herminiimonas sp. Burkholderiales
EAP70851.1 R. solanacearum Burkholderiales
RMP71246.1 P. fluorescens Pseudomonadales
WP 150039291.1 R. atsumiense Rhodospirillales
WP 101253354.1 T. siberiense Rhodospirillales
MBI1214415.1 Alphaproteobacteria
WP 020650478.1 S. variicoloris Nevskiales
WP 202943915.1 M. glucosotrophus Nitrosomonadales
MBS1209187.1 Proteobacteria
WP 058643870.1 P. intermedius Burkholderiales
MBC7466195.1 Bdellovibrio sp. Bdellovibrionales
WP 142697311.1 Bdellovibrio sp. NC01 Bdellovibrionales
MBC7690228.1 Oligoflexia
MBC7658966.1 Oligoflexus sp. Oligoflexales
GBH23700.1 B. vietnamiensis Burkholderiales
EAY65343.1 B. cenocepacia Burkholderiales
WP 221668487.1 Chromobacterium Neisseriales
MBI2254031.1 Proteobacteria
TXH32825.1 R. bacterium Rhodospirillales
WP 010545438.1 S. elodea Sphingomonadales
WP 218191100.1 E. aerosaccus Hyphomicrobiales
WP 068086676.1 N. rosa Sphingomonadales
MBK8176886.1 Rhodospirillales
PZU10152.1 Sphingomonadales
WP 107970199.1 S. oleivorans Sphingomonadales
WP 096512052.1 Pseudomonas sp. LAB-08
WP 091911539.1 C. palmae Burkholderiales
WP 121087356.1 P. silviterrae Burkholderiales
WP 124684214.1 C. pauculus Burkholderiales
ABC38269.1 B. thailandensis E264 Burkholderiales
WP 082852706.1 C. fortuita Burkholderiales
WP 061152643.1 C. arvi Burkholderiales
HveFMCpNR2
RmiFMM pNR
UraFMU pNR
RirFMG pNR
PbrRHI pNR
GthCRYpNR3
GthCRYpNR1
GthCRYpNR2
NgaSEucpNR
EugEgr pNR
SspVCC_pNR
SacVCC pNR
EdeVCC_pNR
KniVKl pNR
BarVBr pNR
TspVCT_pNR
NispSTBpNR
PcoEXCpNRb
PcoEXCpNRa
PkiEXC pNR
AncyANCpNR
Ddi AMB pNR
PpoAMB pNR
GokEXC pNR
GnoGLA pNR
Cpa GLA pNR
CmeRHO pNR
GliRHOFpNR
GsuRHO pNR
cKorach MA
HmoAHalbMA
HacAHalbMA
HadAHalbMA
HafAHalbMA
HabAHalbMA
SapANat MA
0.2

Figure S5

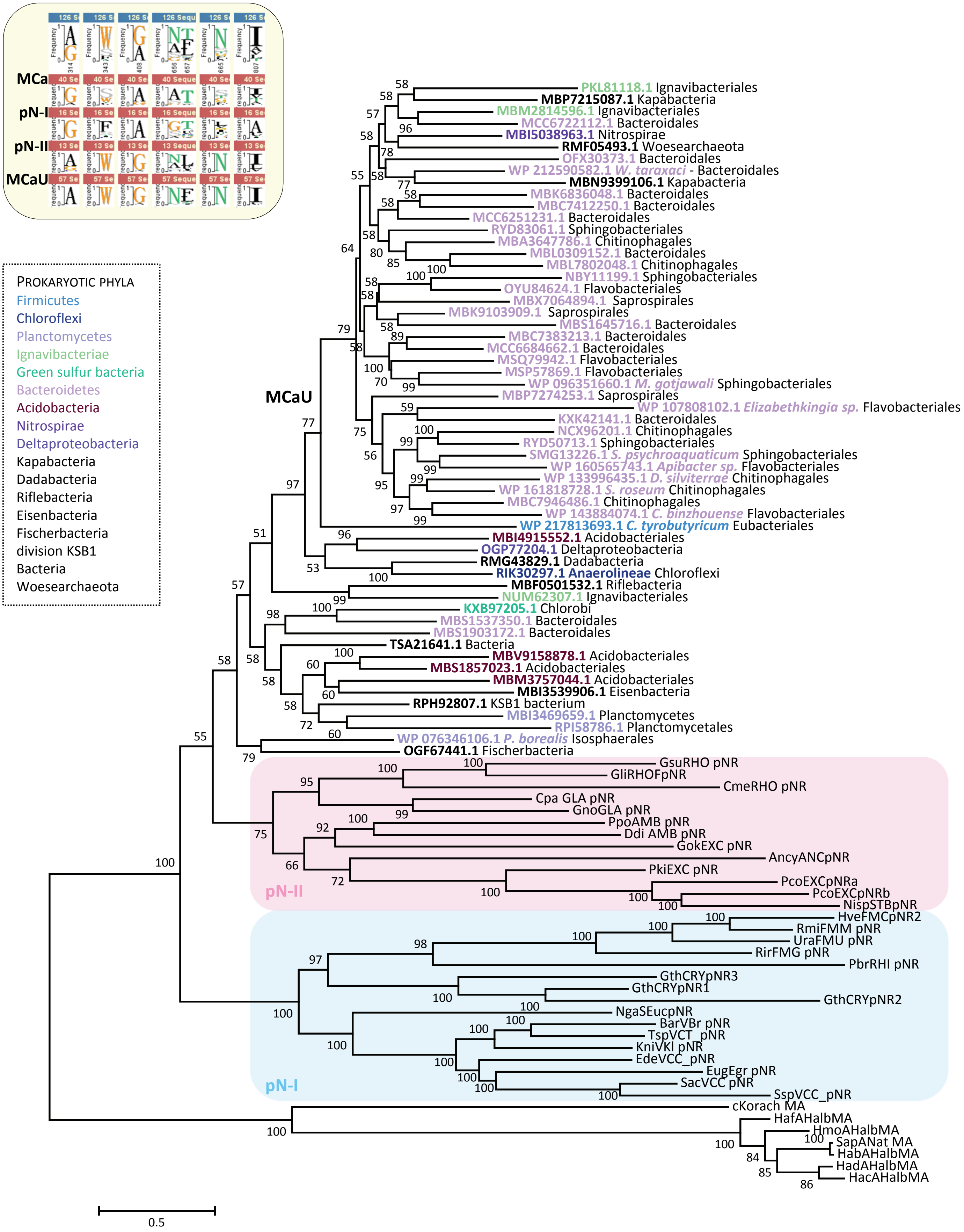
MCa
pN-I
pN-II
MCaU
PROKARYOTIC PHYLA
Firmicutes
Chloroflexi
Planctomycetes
Ignavibacteriae
Green sulfur bacteria
Bacteroidetes
Acidobacteria
Nitrospirae
Deltaproteobacteria
Kapabacteria
Dadabacteria
Riflebacteria
Eisenbacteria
Fischerbacteria
division KSB1
Bacteria
Woesearchaeota
PKL81118.1 Ignavibacteriales
MBP7215087.1 Kapabacteria
MBM2814596.1 Ignavibacteriales
MCC6722112.1 Bacteroidales
MBI5038963.1 Nitrospirae
RMF05493.1 Woesearchaeota
OFX30373.1 Bacteroidales
WP 212590582.1 W. taraxaci - Bacteroidales
MBN9399106.1 Kapabacteria
MBK6836048.1 Bacteroidales
MBC7412250.1 Bacteroidales
MCC6251231.1 Bacteroidales
RYD83061.1 Sphingobacteriales
MBA3647786.1 Chitinophagales
MBL0309152.1 Bacteroidales
MBL7802048.1 Chitinophagales
NBY11199.1 Sphingobacteriales
OYU84624.1 Flavobacteriales
MBX7064894.1 Saprospirales
MBK9103909.1 Saprospirales
MBS1645716.1 Bacteroidales
MBC7383213.1 Bacteroidales
MCC6684662.1 Bacteroidales
MSQ79942.1 Flavobacteriales
MSP57869.1 Flavobacteriales
WP 096351660.1 M. gotjawali Sphingobacteriales
MBP7274253.1 Saprospirales
WP 107808102.1 Elizabethkingia sp. Flavobacteriales
KXK42141.1 Bacteroidales
NCX96201.1 Chitinophagales
RYD50713.1 Sphingobacteriales
SMG13226.1 S. psychroaquaticum Sphingobacteriales
WP 160565743.1 Apibacter sp. Flavobacteriales
WP 133996435.1 D. silviterrae Chitinophagales
WP 161818728.1 S. roseum Chitinophagales
MBC7946486.1 Chitinophagales
WP 143884074.1 C. binzhouense Flavobacteriales
WP 217813693.1 C. tyrobutyricum Eubacteriales
MBI4915552.1 Acidobacteriales
OGP77204.1 Deltaproteobacteria
RMG43829.1 Dadabacteria
RIK30297.1 Anaerolineae Chloroflexi
MBF0501532.1 Riflebacteria
NUM62307.1 Ignavibacteriales
KXB97205.1 Chlorobi
MBS1537350.1 Bacteroidales
MBS1903172.1 Bacteroidales
TSA21641.1 Bacteria
MBV9158878.1 Acidobacteriales
MBS1857023.1 Acidobacteriales
MBM3757044.1 Acidobacteriales
MBI3539906.1 Eisenbacteria
RPH92807.1 KSB1 bacterium
MBI3469659.1 Planctomycetes
RPI58786.1 Planctomycetales
WP 076346106.1 P. borealis Isosphaerales
OGF67441.1 Fischerbacteria
GsuRHO pNR
GliRHOFpNR
CmeRHO pNR
Cpa GLA pNR
GnoGLA pNR
PpoAMB pNR
Ddi AMB pNR
GokEXC pNR
AncyANCpNR
PkiEXC pNR
PcoEXCpNRa
PcoEXCpNRb
NispSTBpNR
pN-II
HveFMCpNR2
RmiFMM pNR
UraFMU pNR
RirFMG pNR
PbrRHI pNR
GthCRYpNR3
GthCRYpNR1
GthCRYpNR2
NgaSEucpNR
BarVBr pNR
TspVCT_pNR
KniVKI pNR
EdeVCC_pNR
EugEgr pNR
SacVCC pNR
SspVCC_pNR
pN-I
cKorach MA
HafAHalbMA
HmoAHalbMA
SapANat MA
HabAHalbMA
HadAHalbMA
HacAHalbMA
0.5

Figure S6A

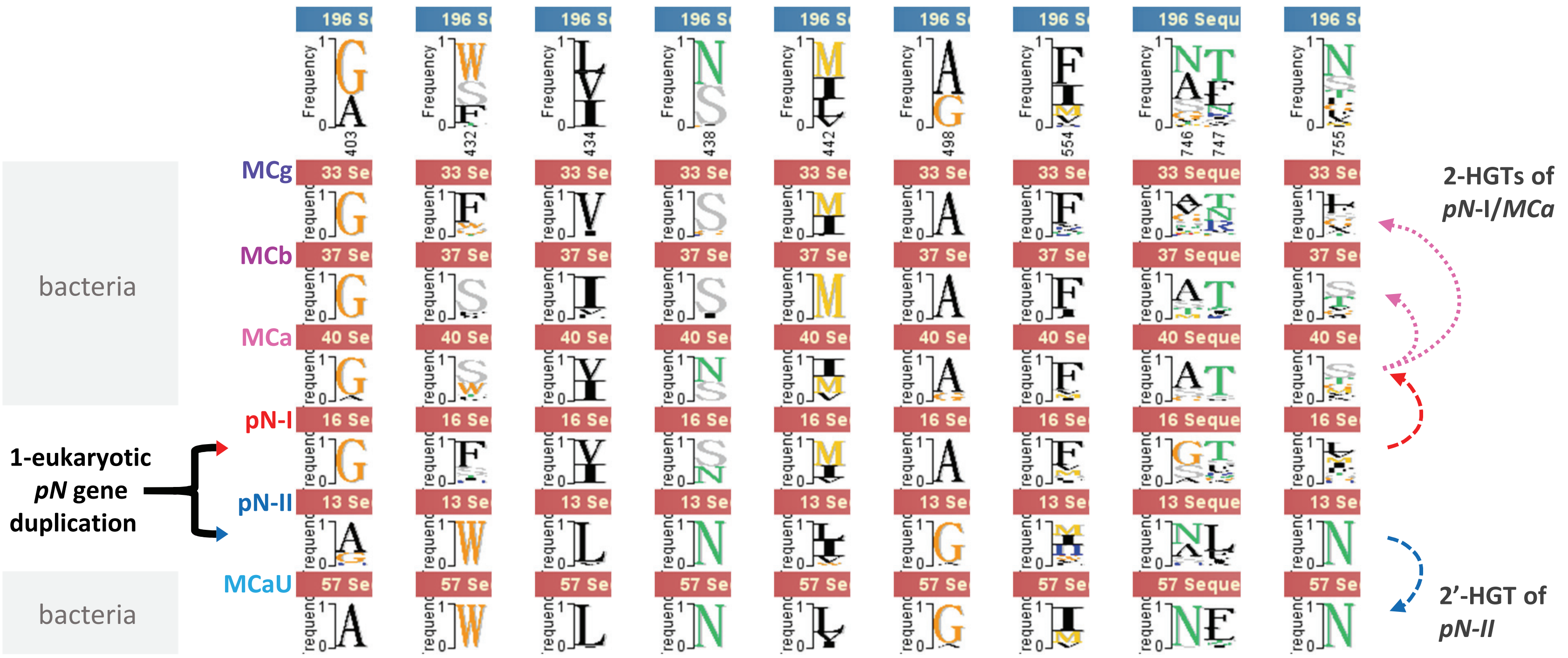
bacteria
1-eukaryotic pN gene duplication
bacteria
MCg
MCb
MCa
pN-I
pN-II
MCaU
196 Se
33 Se
37 Se
40 Se
16 Se
13 Se
57 Se
Frequency
403
432
434
438
442
498
554
746
747
755
2-HGTs of pN-I/MCa
2'-HGT of pN-II

Figure S6B

			MCg	MCb	MCa	MCaU
**Bacteria**			**1973**	**1587**	**7103**	**1060**
**Terrabacteria**			**4**	**1521**	**900**	**27**
	Cyanobacteria				**240**	**2**
	Firmicutes			**1437**	**597**	**15**
		Bacillales		**402**	**561**	
		Lactobacillales		**1029**	**17**	
		Clostridia		**3**	**16**	**14**
	Actinobacteria			**83**	**9**	**2**
	Chloroflexi				**25**	**6**
	Deinococcales				**16**	
	Armatimonadetes				**10**	
	Abitibibacteriaceae				**2**	
**PVC**					**74**	**18**
	Planctomycetes				**21**	**17**
	Verrucomicrobia				**43**	
	Chlamydiae				**3**	
**FCB**			**13**		**1475**	**955**
	Bacteroidetes		**12**		**1443**	**930**
		Cytophagales	**5**		**275**	**36**
		Flavobacteriales	**4**		**668**	**394**
		Chitinophagales	**2**		**162**	**174**
		Bacteroidales			**8**	**7**
		Sphingobacteriales			**285**	**293**
		Saprospirales			**5**	**6**
	Gemmatimonadetes				**25**	
	Ignavibacteriae				**15**	**13**
	Chlorobi				**2**	**3**
	Zixibacteria				**4**	**4**
	Kapabacteria				**2**	**2**
**Acidobacteria**					**67**	**20**
**Proteobacteria (PB)**			**1952**	**60**	**4505**	**20**
	AlphaPB (APB)		**98**		**2604**	**2**
		Sphingomonadales	**66**		**244**	
		Rhodospirillales	**7**		**323**	
		Rhizobiales (Hyphomicrobiales)			**1901**	
		Caulobacterales	**23**		**27**	
		Rhodobacterales			**94**	
	BetaPB (BPB)		**734**	**2**	**388**	**4**
		Burkholderiales	**701**		**370**	**3**
		Neisseriales	**23**		**6**	
		Nitrosomonadales	**8**		**8**	
		Rhodocyclales			**2**	
	GammaPB (GPB)		**1114**	**57**	**1485**	**2**
		Xanthomonadales	**13**		**338**	
		Pseudomonadales	**1092**		**1130**	
		Enterobacterales		**22**	**4**	
		Orbales		**25**		
		Moraxellales		**7**		
		Oceanospirillales			**10**	
	DeltaPB (DPB)				**15**	**10**
**Oligoflexa**			**3**		**6**	**1**

Table S3. Counts of organisms (2+) identified in PHI-Blast searches for MC subgroups (expect threshold 1e-30)

1659 sequences selected

Taxonomy	Number of hits	Number of Organisms	Description
⊟cellular organisms	10314	1185	
. ⊟Bacteria	10311	1184	
. . ⊟FCB group	4941	526	
. . . ⊞Bacteroidetes/Chlorobi group	4938	525	
. . . Candidatus Kryptonia bacterium	3	1	Candidatus Kryptonia bacterium hits
. . ⊞PVC group	12	4	
. . ⊞Bacteria candidate phyla	30	8	
. . ⊟Proteobacteria	378	61	
. . . Proteobacteria bacterium	18	1	Proteobacteria bacterium hits
. . . ⊞Deltaproteobacteria	333	53	
. . . ⊞Gammaproteobacteria	18	5	
. . . Alphaproteobacteria bacterium	3	1	Alphaproteobacteria bacterium hits
. . . Formivibrio citricus	6	1	Formivibrio citricus hits
. . ⊞Acidobacteria	30	5	
. . ⊞unclassified Bacteria	21	4	
. . ⊞Spirochaetes	39	8	
. . Calditrichaeota bacterium	3	1	Calditrichaeota bacterium hits
. . ⊞Synergistetes	9	3	
. . ⊟Terrabacteria group	4641	532	
. . . ⊟Firmicutes	3801	415	
. . . . ⊞Bacilli	435	28	
. . . . ⊞Negativicutes	915	95	
. . . . ⊞Clostridia	2397	283	
. . . . ⊞unclassified Firmicutes sensu stricto	30	3	
. . . . Culicoidibacter larvae	6	1	Culicoidibacter larvae hits
. . . . ⊞environmental samples	6	2	
. . . . Hydrogenispora ethanolica	6	1	Hydrogenispora ethanolica hits
. . . . ⊞Erysipelotrichales	6	2	
. . . ⊞Chloroflexi	12	3	
. . . Armatimonadetes bacterium	15	1	Armatimonadetes bacterium hits
. . . ⊟Actinobacteria	813	113	
. . . . Actinobacteria bacterium RBG_16_64_13	3	1	Actinobacteria bacterium RBG_16_64_13 hits
. . . . ⊞Coriobacteriia	810	112	
. . ⊞Dictyoglomus	36	6	
. . ⊞Caldiserica/Cryosericota group	42	6	
. . ⊞Nitrospirae	51	4	
. . ⊞unclassified Elusimicrobia	42	10	
. . ⊞Coprothermobacter	6	2	
. . ⊞Fusobacteriales	21	2	
. . ⊞Calditerrivibrio	9	2	
. Methermicoccus sp.	3	1	Methermicoccus sp. hits

1659 sequences selected

Taxonomy	Number of hits	Number of Organisms	Description
cellular organisms	10314	1185	
. Bacteria	10311	1184	
. . FCB group	4941	526	
. . . Bacteroidetes/Chlorobi group	4938	525	
. . . . Chlorobi	90	13	
. . . . . Chlorobiaceae	87	12	
. . . . . . Chlorobaculum	18	4	
. . . . . . Chlorobiaceae bacterium	30	1	Chlorobiaceae bacterium hits
. . . . . . Chlorobium/Pelodictyon group	39	7	
. . . . Chlorobium	30	5	
. . . . Pelodictyon	9	2	
. . . . . Chlorobi bacterium	3	1	Chlorobi bacterium hits
. . . . Bacteroidetes	4845	511	
. . . . . Bacteroidia	4740	494	
. . . . . . Bacteroidales	4650	482	
. . . . Prevotellaceae	732	84	
. . . . Prevotellamassilia	12	2	
. . . . Prevotella	6	77	Prevotella hits
. . . . Candidatus Caccomonas pullistercoris	3	1	Candidatus Caccomonas pullistercoris hits
. . . . Candidatus Paraprevotella stercoravium	3	1	Candidatus Paraprevotella stercoravium hits
. . . . Prevotellaceae bacterium	6	1	Prevotellaceae bacterium hits
. . . . Alloprevotella	24	2	
. . . . Tannerellaceae	540	66	
. . . . Parabacteroides	33	59	Parabacteroides hits
. . . . Tannerella	21	6	
. . . . Tannerellaceae bacterium	12	1	Tannerellaceae bacterium hits
. . . . Barnesiellaceae	39	6	
. . . . Coprobacter	27	5	
. . . . Barnesiella sp. GGCC_0306	12	1	Barnesiella sp. GGCC_0306 hits
. . . . Bacteroidaceae	6	233	Bacteroidaceae hits
. . . . Bacteroides	108	199	Bacteroides hits
. . . . unclassified Bacteroidaceae	93	2	
. . . . Phocaeicola	6	27	Phocaeicola hits
. . . . Mediterranea	27	2	
. . . . Caecibacteroides pullorum	6	1	Caecibacteroides pullorum hits
. . . . Candidatus Avibacteroides faecavium	3	1	Candidatus Avibacteroides faecavium hits
. . . . Paludibacteraceae	42	6	
. . . . unclassified Bacteroidales	111	5	
. . . . Porphyromonadaceae	33	4	
. . . . Rikenellaceae	183	19	
. . . . Alistipes	12	16	Alistipes hits
. . . . Rikenellaceae bacterium	3	1	Rikenellaceae bacterium hits
. . . . Gallalistipes aquisgranensis	6	1	Gallalistipes aquisgranensis hits
. . . . Acetobacteroides hydrogenigenes	6	1	Acetobacteroides hydrogenigenes hits
. . . . Dysgonomonadaceae	141	26	
. . . . Dysgonamonadaceae bacterium	6	1	Dysgonamonadaceae bacterium hits
. . . . Seramator thermalis	3	1	Seramator thermalis hits
. . . . Dysgonomonadaceae bacterium	3	1	Dysgonomonadaceae bacterium hits
. . . . Dysgonomonas	129	23	
. . . . Odoribacteraceae	267	30	
. . . . Odoribacter	153	14	
. . . . Culturomica	6	2	
. . . . Butyricimonas	6	13	Butyricimonas hits
. . . . Odoribacteraceae bacterium	12	1	Odoribacteraceae bacterium hits
. . . . Bacteroidales incertae sedis	6	2	
. . . . Sodaliphilus pleomorphus	6	1	Sodaliphilus pleomorphus hits
. . . . . . Marinilabiliales	75	11	
. . . . Marinilabiliales bacterium	3	1	Marinilabiliales bacterium hits
. . . . Prolixibacteraceae	57	7	
. . . . Prolixibacteraceae bacterium	24	1	Prolixibacteraceae bacterium hits
. . . . Mangrovibacterium	12	2	
. . . . Maribellus	18	3	
. . . . Aquipluma nitroreducens	3	1	Aquipluma nitroreducens hits
. . . . Marinifilaceae	15	3	
. . . . . . Bacteroidia bacterium	15	1	Bacteroidia bacterium hits
. . . . . unclassified Bacteroidetes	102	16	
. . . . . Sphingobacteriia bacterium	3	1	Sphingobacteriia bacterium hits
. . . . Ignavibacteriae bacterium	3	1	Ignavibacteriae bacterium hits
. . . Candidatus Kryptonia bacterium	3	1	Candidatus Kryptonia bacterium hits
. . PVC group	12	4	
. . . Kiritimatiellaeota bacterium	3	1	Kiritimatiellaeota bacterium hits
. . . Lentisphaerae	9	3	
. . Bacteria candidate phyla	30	8	
. . . Candidatus Eisenbacteria bacterium	9	1	Candidatus Eisenbacteria bacterium hits
. . . candidate division KSB1 bacterium	3	1	candidate division KSB1 bacterium hits
. . . Candidatus Wallbacteria	6	2	
. . . . Candidatus Wallbacteria bacterium GWC2_49_35	3	1	Candidatus Wallbacteria bacterium GWC2_49_35 hits
. . . . Candidatus Wallbacteria bacterium	3	1	Candidatus Wallbacteria bacterium hits
. . . Candidatus Poribacteria bacterium	3	1	Candidatus Poribacteria bacterium hits
. . . Candidatus Riflebacteria bacterium	3	1	Candidatus Riflebacteria bacterium hits
. . . Candidatus Aminicenantes bacterium	3	1	Candidatus Aminicenantes bacterium hits
. . . Candidatus Wirthbacteria bacterium CG2_30_54_11	3	1	Candidatus Wirthbacteria bacterium CG2_30_54_11 hits
. . Proteobacteria	378	61	
. . . Proteobacteria bacterium	18	1	Proteobacteria bacterium hits
. . . Deltaproteobacteria	333	53	
. . . . Desulfobacterales	12	4	
. . . . unclassified Deltaproteobacteria	72	5	
. . . . Desulfuromonadales	81	12	
. . . . . Desulfuromonadales bacterium	12	1	Desulfuromonadales bacterium hits
. . . . . Geobacteraceae	69	11	
. . . . . . unclassified Geobacteraceae	24	4	
. . . . . . Geobacter	30	4	
. . . . . . Trichlorobacter	15	3	
. . . . Desulfovibrionales	147	25	
. . . . . Desulfovibrionaceae	144	24	
. . . . . . Desulfovibrio	93	10	
. . . . . . Fundidesulfovibrio putealis	3	1	Fundidesulfovibrio putealis hits
. . . . . . Solidesulfovibrio	30	9	
. . . . . . Desulfovibrionaceae bacterium	3	1	Desulfovibrionaceae bacterium hits
. . . . . . Cupidesulfovibrio	15	3	
. . . . . Desulfovibrionales bacterium GWA2_65_9	3	1	Desulfovibrionales bacterium GWA2_65_9 hits
. . . . Syntrophaceae	9	3	
. . . . SAR324 cluster bacterium	3	1	SAR324 cluster bacterium hits
. . . . uncultured delta proteobacterium	3	1	uncultured delta proteobacterium hits
. . . . unclassified Syntrophobacterales	6	2	
. . . Gammaproteobacteria	18	5	
. . . . Salmonella enterica subsp. enterica serovar Enteritidis	3	1	Salmonella enterica subsp. enterica serovar Enteritidis hits
. . . . Alteromonadales	15	4	
. . . . . Pseudoalteromonas sp. Z9A5	3	1	Pseudoalteromonas sp. Z9A5 hits
. . . . . Shewanella	9	2	
. . . . . Psychromonas sp. L1A2	3	1	Psychromonas sp. L1A2 hits
. . . Alphaproteobacteria bacterium	3	1	Alphaproteobacteria bacterium hits
. . . Formivibrio citricus	6	1	Formivibrio citricus hits
. . Acidobacteria	30	5	
. . . unclassified Acidobacteria	15	2	
. . . Holophagae	15	3	
. . unclassified Bacteria	21	4	
. . Spirochaetes	39	8	
. . Calditrichaeota bacterium	3	1	Calditrichaeota bacterium hits
. . Synergistetes	9	3	
. . . unclassified Synergistetes	6	2	
. . . Synergistaceae bacterium	3	1	Synergistaceae bacterium hits
. . Terrabacteria group	4641	532	
. . . Firmicutes	3801	415	
. . . . Bacilli	435	28	
. . . . . Bacillales	24	3	
. . . . . . Tumebacillus	9	2	
. . . . . . Paenibacillaceae bacterium	15	1	Paenibacillaceae bacterium hits
. . . . . Streptococcaceae	411	25	
. . . . . . Lactococcus	15	24	Lactococcus hits
. . . . . . Lactovum miscens	6	1	Lactovum miscens hits
. . . . Negativicutes	915	95	
. . . . . Veillonellaceae	606	49	
. . . . . . Veillonella	21	42	Veillonella hits
. . . . . . Megasphaera	27	5	
. . . . . . Lucifera butyrica	6	1	Lucifera butyrica hits
. . . . . . Massilibacillus massiliensis	3	1	Massilibacillus massiliensis hits
. . . . . Acidaminococcaceae	66	5	
. . . . . . Phascolarctobacterium	60	3	
. . . . . . Acidaminococcaceae bacterium	3	1	Acidaminococcaceae bacterium hits
. . . . . . Succinispira mobilis	3	1	Succinispira mobilis hits
. . . . . Negativicutes bacterium	18	1	Negativicutes bacterium hits
. . . . . Selenomonadales	225	40	
. . . . . . Sporomusaceae	99	24	
. . . . Anaeromusa acidaminophila	3	1	Anaeromusa acidaminophila hits
. . . . Anaeroarcus burkinensis	3	1	Anaeroarcus burkinensis hits
. . . . Propionispora	18	4	
. . . . unclassified Sporomusaceae	9	2	
. . . . Anaerospora hongkongensis	6	1	Anaerospora hongkongensis hits
. . . . Pelosinus	3	10	Pelosinus hits
. . . . Methylomusa anaerophila	6	1	Methylomusa anaerophila hits
. . . . Sporolituus	6	2	
. . . . Thermosinus	6	2	
. . . . . . Selenomonadaceae	126	16	
. . . . Megamonas	6	8	Megamonas hits
. . . . Pectinatus	27	5	
. . . . Selenomonas sp.	3	1	Selenomonas sp. hits
. . . . Propionispira	9	2	
. . . . Clostridia	2397	283	
. . . . . Eubacteriales	9	218	Eubacteriales hits
. . . . . . Clostridiaceae	978	99	
. . . . Clostridium	24	90	Clostridium hits
. . . . Desnuesiella massiliensis	3	1	Desnuesiella massiliensis hits
. . . . Hungatella	15	2	
. . . . Caloramator	18	4	
. . . . Clostridiaceae bacterium	6	1	Clostridiaceae bacterium hits
. . . . Fonticella tunisiensis	6	1	Fonticella tunisiensis hits
. . . . . . Oscillospiraceae	66	14	
. . . . Sporobacter	6	2	
. . . . unclassified Caproiciproducens	12	2	
. . . . Faecalibacterium prausnitzii	3	1	Faecalibacterium prausnitzii hits
. . . . Ethanoligenens	18	2	
. . . . Ruminococcus	15	5	
. . . . Caproicibacter fermentans	9	1	Caproicibacter fermentans hits
. . . . Oscillospiraceae bacterium N12	3	1	Oscillospiraceae bacterium N12 hits
. . . . . . unclassified Eubacteriales	30	5	
. . . . . . Lachnospiraceae	9	79	Lachnospiraceae hits
. . . . Lacrimispora	12	3	
. . . . Dorea	144	6	
. . . . Lachnoclostridium	9	2	
. . . . Butyrivibrio sp.	3	1	Butyrivibrio sp. hits
. . . . Roseburia	3	6	Roseburia hits
. . . . unclassified Lachnospiraceae	51	13	
. . . . Blautia	132	30	
. . . . Agathobacter sp.	15	1	Agathobacter sp. hits
. . . . Anaerocolumna	18	5	
. . . . Fusicatenibacter	96	2	
. . . . Faecalicatena fissicatena	27	1	Faecalicatena fissicatena hits
. . . . [Ruminococcus] lactaris	3	1	[Ruminococcus] lactaris hits
. . . . Anaerostipes	3	6	Anaerostipes hits
. . . . Lachnospiraceae bacterium CAG:25	3	1	Lachnospiraceae bacterium CAG:25 hits
. . . . . . Eubacteriaceae	126	11	
. . . . Acetobacterium	15	3	
. . . . Eubacterium	3	8	Eubacterium hits
. . . . . . Peptostreptococcaceae	24	2	
. . . . . . Thermohydrogenium kirishiense	3	1	Thermohydrogenium kirishiense hits
. . . . . . Eubacteriales incertae sedis	18	3	
. . . . . . Heliobacteriaceae	21	3	
. . . . . Thermoanaerobacterales	333	64	
. . . . . . Thermoanaerobacterales Family III. Incertae Sedis	168	33	
. . . . Caldicellulosiruptor	87	20	
. . . . Thermoanaerobacterium	3	13	Thermoanaerobacterium hits
. . . . . . Thermodesulfobium	12	3	
. . . . . . Thermoanaerobacteraceae	153	28	
. . . . Caldanaerobacter	42	4	
. . . . Calorimonas adulescens	6	1	Calorimonas adulescens hits
. . . . Thermoanaerobacter	6	20	Thermoanaerobacter hits
. . . . Caldanaerobius	9	3	
. . . . . Clostridia bacterium	3	1	Clostridia bacterium hits
. . . . unclassified Firmicutes sensu stricto	30	3	
. . . . Culicoidibacter larvae	6	1	Culicoidibacter larvae hits
. . . . environmental samples	6	2	
. . . . Hydrogenispora ethanolica	6	1	Hydrogenispora ethanolica hits
. . . . Erysipelotrichales	6	2	
. . . Chloroflexi	12	3	
. . . Armatimonadetes bacterium	15	1	Armatimonadetes bacterium hits
. . . Actinobacteria	813	113	
. . . . Actinobacteria bacterium RBG_16_64_13	3	1	Actinobacteria bacterium RBG_16_64_13 hits
. . . . Coriobacteriia	810	112	
. . . . . Coriobacteriales	807	111	
. . . . . . Atopobiaceae	93	24	
. . . . . . Coriobacteriaceae	714	87	
. . . . . Coriobacteriia bacterium	3	1	Coriobacteriia bacterium hits
. . Dictyoglomus	36	6	
. . Caldiserica/Cryosericota group	42	6	
. . Nitrospirae	51	4	
. . unclassified Elusimicrobia	42	10	
. . Coprothermobacter	6	2	
. . Fusobacteriales	21	2	
. . Calditerrivibrio	9	2	
. Methermicoccus sp.	3	1	Methermicoccus sp. hits

Table S4 MntH B is restricted to anaerobic bacteria

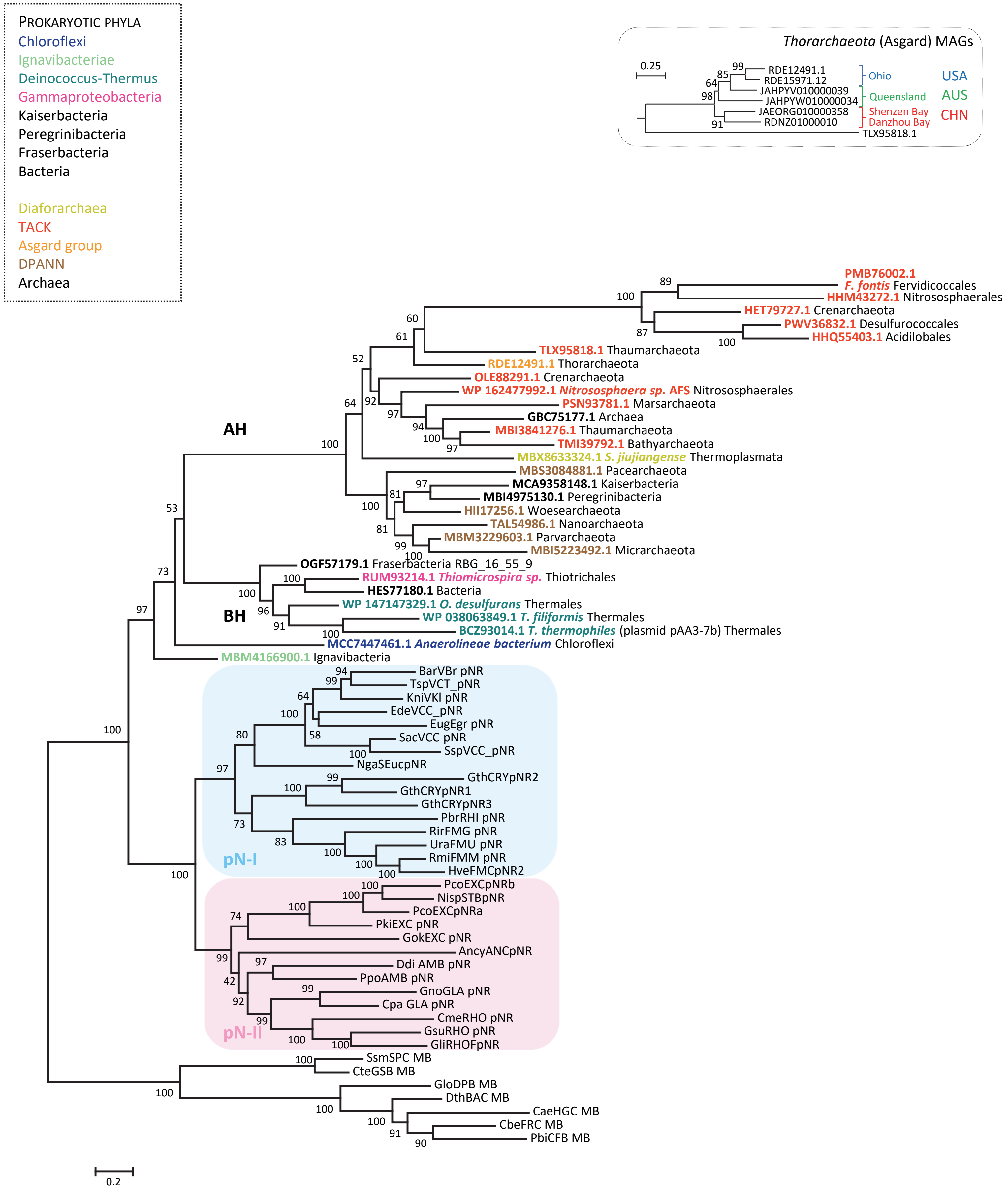
PROKARYOTIC PHYLA
Chloroflexi
Ignavibacteriae
Deinococcus-Thermus
Gammaproteobacteria
Kaiserbacteria
Peregrinibacteria
Fraserbacteria
Bacteria
Diaforarchaea
TACK
Asgard group
DPANN
Archaea
Thorarchaeota (Asgard) MAGs
0.25
RDE12491.1
RDE15971.12
JAHPYV010000039
JAHPYW010000034
JAEORG010000358
RDNZ01000010
TLX95818.1
Ohio
Queensland
Shenzen Bay
Danzhou Bay
USA
AUS
CHN
AH
BH
PMB76002.1 F. fontis Fervidicoccales
HHM43272.1 Nitrososphaerales
HET79727.1 Crenarchaeota
PWV36832.1 Desulfurococcales
HHQ55403.1 Acidilobales
TLX95818.1 Thaumarchaeota
RDE12491.1 Thorarchaeota
OLE88291.1 Crenarchaeota
WP 162477992.1 Nitrososphaera sp. AFS Nitrososphaerales
PSN93781.1 Marsarchaeota
GBC75177.1 Archaea
MBI3841276.1 Thaumarchaeota
TMI39792.1 Bathyarchaeota
MBX8633324.1 S. jiujiangense Thermoplasmata
MBS3084881.1 Pacearchaeota
MCA9358148.1 Kaiserbacteria
MBI4975130.1 Peregrinibacteria
HII17256.1 Woesearchaeota
TAL54986.1 Nanoarchaeota
MBM3229603.1 Parvarchaeota
MBI5223492.1 Micrarchaeota
OGF57179.1 Fraserbacteria RBG_16_55_9
RUM93214.1 Thiomicrospira sp. Thiotrichales
HES77180.1 Bacteria
WP 147147329.1 O. desulfurans Thermales
WP 038063849.1 T. filiformis Thermales
BCZ93014.1 T. thermophiles (plasmid pAA3-7b) Thermales
MCC7447461.1 Anaerolineae bacterium Chloroflexi
MBM4166900.1 Ignavibacteria
BarVBr pNR
TspVCT_pNR
KniVKl pNR
EdeVCC_pNR
EugEgr pNR
SacVCC pNR
SspVCC_pNR
NgaSEucpNR
GthCRYpNR2
GthCRYpNR1
GthCRYpNR3
PbrRHI pNR
RirFMG pNR
UraFMU pNR
RmiFMM pNR
HveFMCpNR2
pN-I
PcoEXCpNRb
NispSTBpNR
PcoEXCpNRa
PkiEXC pNR
GokEXC pNR
AncyANCpNR
Ddi AMB pNR
PpoAMB pNR
GnoGLA pNR
Cpa GLA pNR
CmeRHO pNR
GsuRHO pNR
GliRHOFpNR
pN-II
SsmSPC MB
CteGSB MB
GloDPB MB
DthBAC MB
CaeHGC MB
CbeFRC MB
PbiCFB MB
0.2

Figure S7A

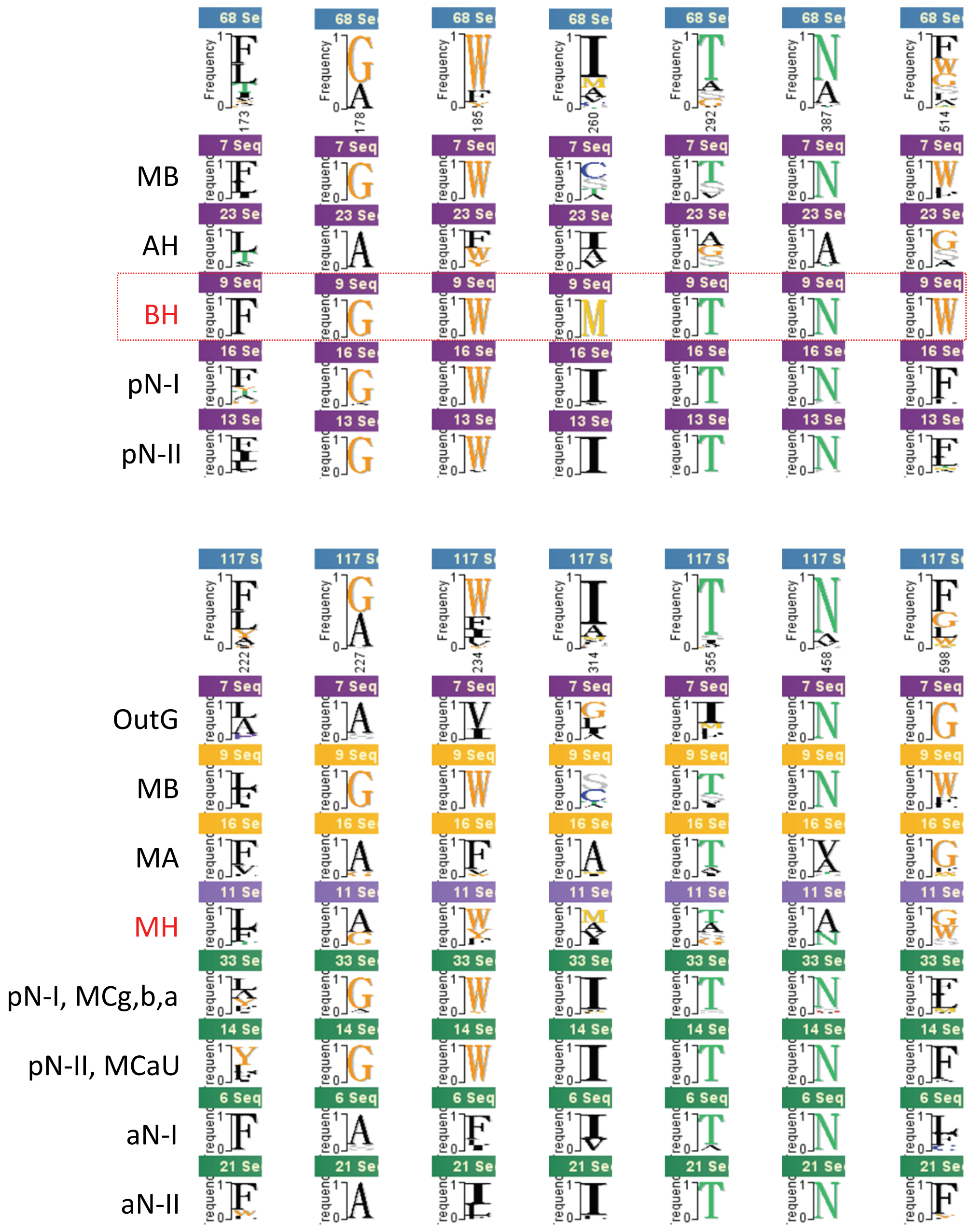

Figure S7B

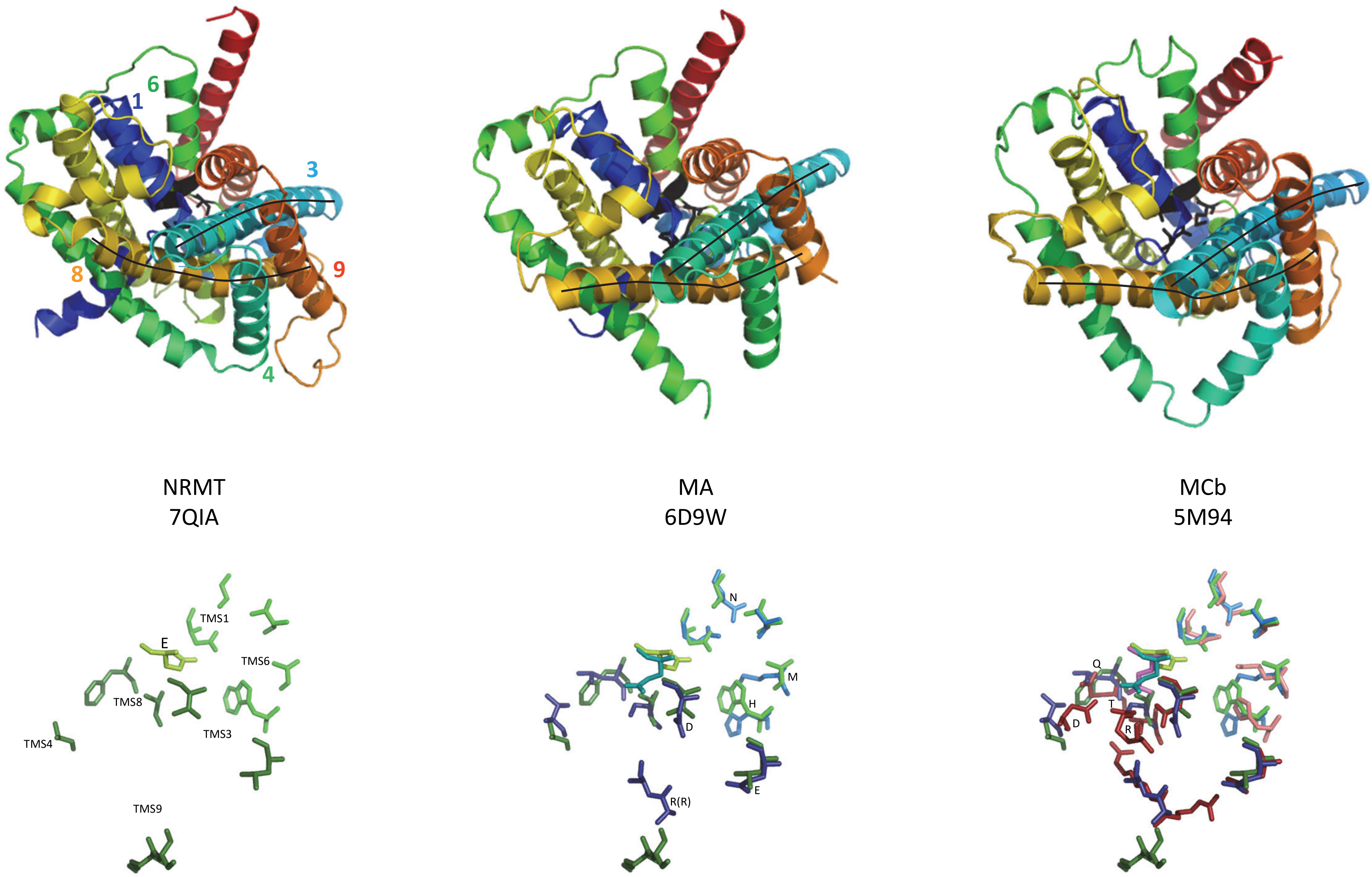
1
6
3
9
8
4
NRMT
7QIA
MA
6D9W
MCb
5M94
TMS1
E
TMS6
TMS8
TMS3
TMS4
TMS9
N
M
H
D
R(R)
E
Q
T
D
R

Figure S8

	Res#							OutG	MB	MA	MH	pN-I_MCabg	pN-II_MCaU	aN-I	aN-II
228	TYR49	0.76	-16.59	0.59	71	0	16.58	F	F	Y	Y	Y	Y	Yf	Yf
230	ASP51	0.82	-25.25	1.00	25	0	30.13	G	D	D	D	D	D	D	D
233	ASN54	0.82	-19.38	1.00	25	0	30.13	T	N	N	N	N	N	N	N
235	SER56	0.52	-17.05	0.60	85	10	14.82	Ta	Av	A	AG	Alvis	A	Ead	Ev
237	SER58	0.55	-18.85	0.69	85	7	14.43	Ac	N	N	DSan	SDa	D	D	Dag
259	ALA80	0.54	-26.62	0.63	89	7	16.47	Ta	L	A	Ag	Asg	A	AG	Ga
276	ASP97	0.51	-16.95	0.58	96	3	10.07	Gdn	Cs	Ngts	DSgnv	Dgs	Dvh	NHd	Hgd
307	TRP115	0.66	-12.49	0.38	78	0	11.14	AILTVF	Litg	Wf	FWl	Wy	Wfy	W	W
311	GLU119	0.65	-15.33	0.52	64	14	15.58	ASv	MVfy	E	FEav	Eq	Eq	E	Eq
314	ILE122	0.54	-15.81	0.52	96	3	12.18	LGai	CSat	Aim	IVAM	Ilmv	I	VI	Il
317	THR125	0.62	-15.33	0.50	75	3	10.71	SNt	T	T	T	CT	Tc	ASc	Sa
318	ASP126	0.77	-13.71	0.58	46	0	22.06	Ast	Asd	D	D	Dae	D	D	D
320	ALA128	0.48	-29.30	0.98	64	0	31.09	Y	A	A	A	At	A	P	Ql
322	VAL130	0.54	-16.40	0.45	92	3	8.13	Ast	Liv	Fvi	Fvy	Vil	Vi	V	Vi
324	GLY132	0.82	-18.78	0.93	25	0	18.51	Nd	G	G	G	G	G	G	G
347	PRO144	0.80	-7.86	0.33	42	39	6.89	Sdghk	Ps	Ps	P	Pns	P	Pq	P
349	PHE146	0.51	-9.17	0.36	96	0	6.99	Npqrt	GLNPRKW	Liptvfm	ITVL	LWPIesaftv	FLMIev	W	Wl
351	ALA148	0.73	-10.89	0.49	64	14	10.07	FWlv	G	AG	GAs	Gas	G	G	G
358	ASP155	0.51	-20.54	0.54	85	3	13.51	AFILTV	LVafi	AIVgl	D	Dcg	D	Sg	D
386	ARG168	0.77	-7.93	0.49	53	10	15.51	Kqr	HKqrst	Rhkq	Rg	Rdms	R	R	Rq
406	GLN188	0.74	-7.38	0.27	67	32	4.13	Tads	E	Eaq	Enq	Edlq	Eq	Ev	Elqm
448	GLY227	0.76	-14.37	0.47	75	14	10.31	Ag	Sga	Gas	Gs	Ga	G	Sa	Gsa
449	ILE228	0.67	-17.41	0.59	78	17	12.98	Li	Vim	Ivm	Iv	Iv	Im	L	Ilvm
452	ALA231	0.79	-16.57	0.43	60	14	15.15	T	A	A	A	As	A	As	Ac
455	MET234	0.81	-18.70	0.99	25	21	32.75	V	M	M	M	M	M	M	Mt
457	HIS236	0.82	-21.09	1.00	25	0	30.85	Y	H	H	H	Hq	H	H	H
463	SER242	0.88	-8.31	0.20	42	21	3.75	F	S	Sp	S	St	Sg	S	S
594	LEU273	0.70	-14.37	0.52	75	14	13.20	VIl	Ml	LM	Lf	Lf	L	L	Lm
597	ALA276	0.59	-25.37	0.78	75	0	22.18	G	G	A	A	As	A	AS	Sa
598	MET277	0.55	-13.21	0.55	89	3	10.06	G	Wlf	Gmwf	WGs	LFvm	Fa	FLc	Fly
601	ASN280	0.82	-22.86	0.86	25	0	16.34	Ts	N	N	N	N	N	N	N
604	LEU283	0.61	-18.15	0.64	85	0	11.00	Iv	M	Ml	I	Il	I	IV	Vli
607	MET286	0.70	-9.14	0.40	89	10	6.55	Ta	MLVi	VMlst	Mlsv	LVimt	VLmt	Vm	V
689	GLN341	0.65	-15.32	0.51	85	10	10.33	FLi	VILf	Lfvi	IL	Qtil	Q	Q	Qs
692	THR344	0.70	-13.45	0.56	78	17	11.92	TAs	St	St	St	Tgs	Ta	Tc	T
695	GLY347	0.76	-14.66	0.51	82	0	10.50	As	SA	Ga	Gas	Gsca	G	G	G
696	THR348	0.63	-18.50	0.93	46	42	26.59	P	Gas	Tav	Tv	Tv	T	T	Ta
697	ILE349	0.63	-12.46	0.62	85	10	9.51	Lm	M	Myl	Li	Lvmi	Lim	Yf	Yfl
699	GLY351	0.86	-9.63	0.25	57	3	5.35	GTA	Gae	G	Ga	G	G	G	G
700	GLN352	0.64	-17.58	0.72	57	32	23.48	A	Gav	Qads	Qmv	Qer	Q	Q	Q
701	VAL353	0.47	-13.92	0.55	96	0	10.32	Yi	NTS	Veimt	AViks	VIafm	Ivy	FY	Fiy
703	MET355	0.64	-13.63	0.43	82	7	10.87	AVt	IFtwy	Mi	FMlv	Mlvs	M	M	Mit
704	GLU356	0.56	-17.56	0.60	78	21	14.02	ACst	Ast	Qdek	Ed	Erstd	E	EQd	Eags
706	PHE358	0.69	-11.68	0.22	85	10	3.41	MACI	FILM	Fl	LFmy	Fy	YFh	F	F
733	ARG368	0.63	-17.23	0.59	67	28	17.95	IVt	Gs	Rct	Rgivl	Rlq	Rq	R	Rs
734	ARG369	0.46	-18.54	0.55	89	0	18.73	SAFW	Vlti	R	R	Ra	R	AN	Vmtail
738	ARG373	0.68	-25.17	1.00	53	0	23.85	-	-	-	R	R	R	R	R
741	ALA376	0.55	-21.05	0.88	67	10	22.64	-	-	-	TNav	Agts	A	A	Ag
744	PRO379	0.74	-13.42	0.31	46	0	7.70	TVF	GPaiv	P	P	Pa	P	P	P
745	VAL380	0.56	-12.19	0.58	100	0	15.08	G	As	Agps	TAfl	Acsv	As	S	Tgas
771	SER406	0.64	-15.86	0.67	67	10	15.57	A	S	Sg	St	Sat	S	AS	Lv
772	GLN407	0.69	-21.86	0.90	46	0	30.52	Q	Q	Q	Q	Q	Q	Sq	N
775	LEU410	0.65	-20.06	0.96	46	0	36.07	N	L	L	L	L	L	L	Qm
776	ALA411	0.79	-13.96	0.96	25	25	35.16	G	Sa	S	S	Sa	S	Sa	Samn
785	PRO420	0.71	-12.09	0.36	64	0	10.31	Fimvy	LTPsv	Pa	P	P	P	P	P
796	MET431	0.85	-12.36	0.45	28	17	8.20	Lm	M	M	M	Ml	M	Mv	M
879	ASN456	0.81	-12.33	0.75	25	39	25.01	Gs	Nd	N	N	Nds	N	N	Nd

Table S5. Multi-RELIEF prediction of Slc11 specificity residues based on eight defined phylogroups (cf Figure 2). The negative SH Z-score column, left, and positive mR Z-score column, right, list per site standard deviations below or above the mean of 100 randomizations for non-overlapping group residue compositions and type II rate-shift detection, respectively [10].

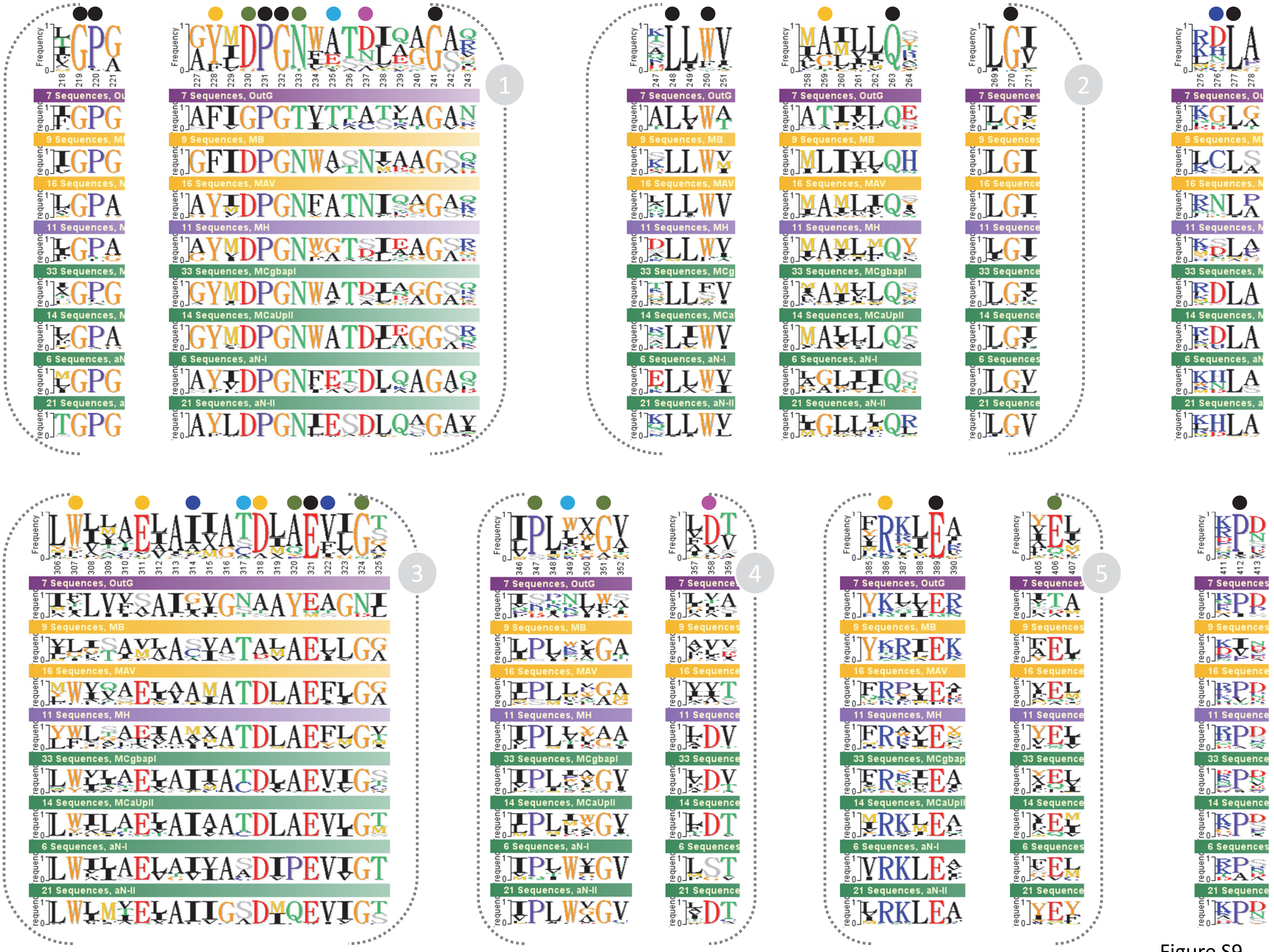

1
2
3
4
5
7 Sequences, OutG
9 Sequences, MB
16 Sequences, MAV
11 Sequences, MH
33 Sequences, MCgbapl
14 Sequences, MCaUpII
6 Sequences, aN-I
21 Sequences, aN-II
Frequency

Figure S9

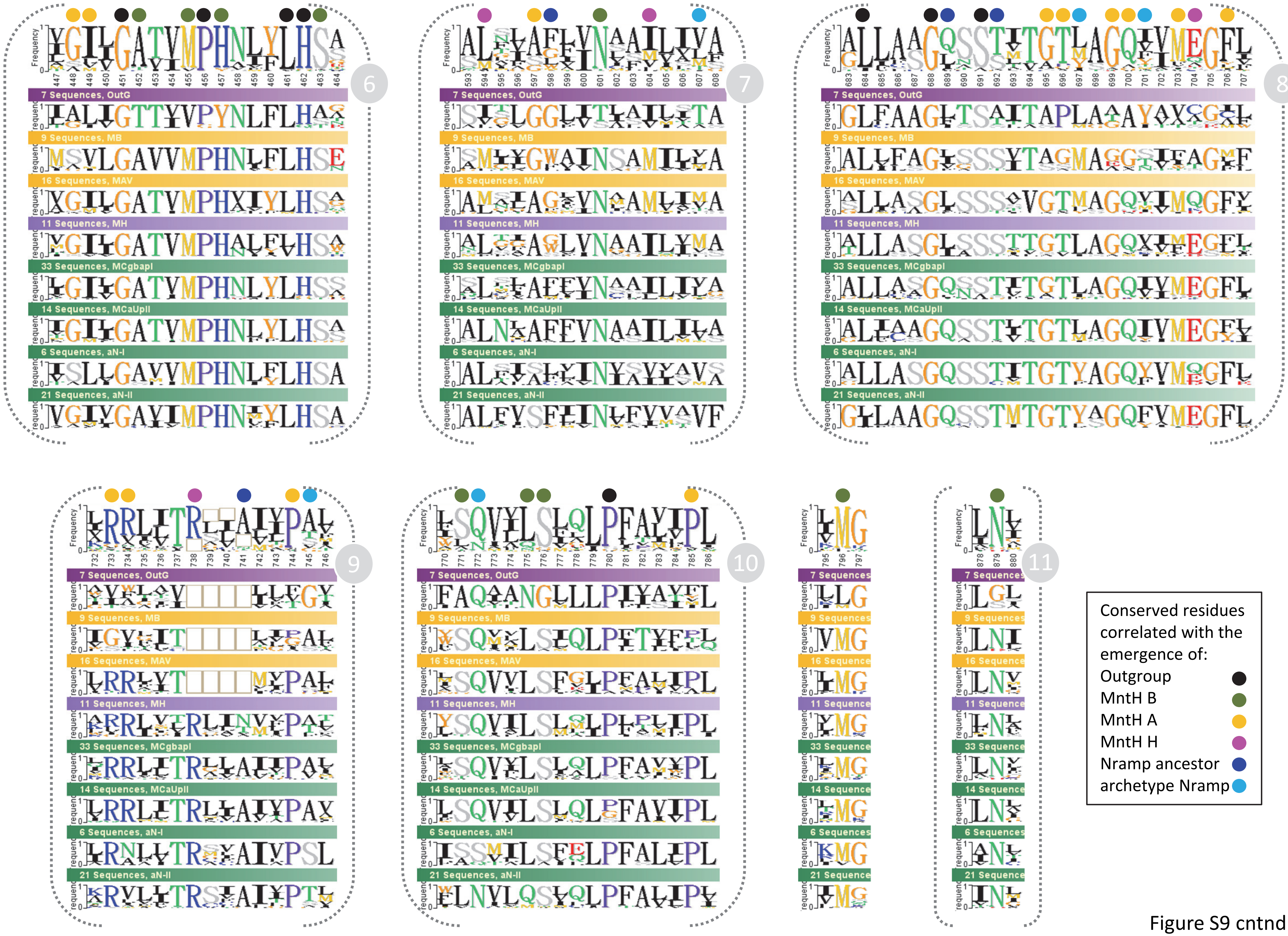

Figure S9 cntnd
